# Bayesian causal graphical model for joint Mendelian randomization analysis of multiple exposures and outcomes

**DOI:** 10.1016/j.ajhg.2025.03.005

**Published:** 2025-04-02

**Authors:** Verena Zuber, Toinét Cronjé, Na Cai, Dipender Gill, Leonardo Bottolo

**Affiliations:** 1 Department of Epidemiology and Biostatistics, School of Public Health, Imperial College London, London, UK; 2 MRC Centre for Environment and Health, School of Public Health, Imperial College London, London, UK; 3 UK Dementia Research Institute, Imperial College London, London, UK; 4 Department of Public Health, University of Copenhagen, Copenhagen, Denmark; 5 Helmholtz Pioneer Campus, Helmholtz Munich, Neuherberg, Germany; 6 Computational Health Centre, Helmholtz Munich, Neuherberg, Germany; 7 School of Medicine and Health, Technical University of Munich, Munich, Germany; 8 Department of Genomic Medicine, School of Clinical Medicine, University of Cambridge, Cambridge, UK; 9 MRC Biostatistics Unit, School of Clinical Medicine, University of Cambridge, Cambridge, UK

**Keywords:** Mendelian randomization, causal graphical models, causal inference, interventional calculus, mental health, psychiatric genetics

## Abstract

Current Mendelian randomization (MR) methods do not reflect complex relationships among multiple exposures and outcomes as is typical for real-life applications. We introduce MrDAG, a Bayesian causal graphical model for summary-level MR analysis to detect dependency relations within the exposures, the outcomes, and between them to improve causal effects estimation. MrDAG combines three causal inference strategies. It uses genetic variation as instrumental variables to account for unobserved confounders. It performs structure learning to detect and orientate the direction of the dependencies within the exposures and the outcomes. Finally, interventional calculus is employed to derive principled causal effect estimates. In MrDAG the directionality of the causal effects between the exposures and the outcomes is assumed known, i.e., the exposures can only be potential causes of the outcomes, and no reverse causation is allowed. In the simulation study, MrDAG outperforms recently proposed one-outcome-at-a-time and multi-response multi-variable Bayesian MR methods as well as causal graphical models under the constraint on edges’ orientation from the exposures to the outcomes. MrDAG was motivated to unravel how lifestyle and behavioral exposures impact mental health. It highlights first, education and second, smoking as effective points of intervention given their important downstream effects on mental health. It also enables the identification of a novel path between smoking and the genetic liability to schizophrenia and cognition, demonstrating the complex pathways toward mental health. These insights would have been impossible to delineate without modeling the paths between multiple exposures and outcomes at once.

## Teaser

In real-life applications, exposures and outcomes are interconnected in complex relationships that cannot be modeled using available Mendelian randomization methods. We introduce MrDAG, a Bayesian causal graphical model to detect dependency relations between exposures and between outcomes to better estimate causal effects and shed light on complex pathways toward disease.

## Introduction

Genetic evidence is increasingly used to infer causal relationships between human traits in Mendelian randomization (MR) analysis. The standard MR paradigm, one exposure and one outcome, can be biased by unmeasured pleiotropy. It occurs when the genetic variants used as instruments in the MR analysis act via separate pathways to the exposure under investigation. Extensions to consider multiple exposures^1^ along with multi-response^2^ of standard MR allow to model pleiotropy, acting via any of the exposures or any of the outcomes or both, respectively.

These and similar methods suffer an important limitation, since they are not designed to account for the dependency relations within the exposures and the outcomes to enhance the detection of causal effects between them and improve their accuracy. As we show in our motivating data application on mental health phenotypes, it is a common problem in practical applications that the effect of an exposure on an outcome can be confounded or (partially or completely) mediated by another exposure^3^ or mediated by another outcome, or both. However, this structure is latent and not known and consequently needs to be learned from the data.

The first attempt to provide a solution to this problem is a Bayesian network algorithm presented by Howey et al.^4^ Based on individual-level data, they apply a score-based method to determine the dependency structure among the variables (genetic variants and traits) under the constraint of directionality between the genetic variants used as instrumental variables (IVs) (called genetic anchors) and the traits. This is the only assumption regarding the directionality, so their method can be used in a “bidirectional” or “reciprocal” fashion to determine the direction of causation between two traits. However, unobserved confounding that operates between traits that are not directly linked with the genetic anchors might bias the results. Moreover, the directed acyclic graph (DAG) that they identify might not be unique, since other DAGs can hold the same conditional independencies and, thus, the same score. A similar approach for individual-level data is proposed in Badsha and Fu^5^ with a constraint-based method to detect the dependency relations among the variables. Besides the problem of unobserved confounders, since the data could be of mixed type (discrete and continuous variables), the specification of a unique type of conditional independence test for the entire dataset is also problematic.

Other solutions to this problem have been proposed recently from the same lab.^6^^,^^7^^,^^8^^,^^9^^,^^10^ They are bidirectional MR models that consider the problem of invalid and weak IVs with inference performed by using a frequentist approach. Some of these methods have been developed for individual-level data,^8^^,^^10^ while others work with summary-level statistics^6^^,^^7^^,^^9^ Lin et al.^6^^,^^7^^,^^9^ present a two-step approach that first utilizes bidirectional MR on every pair of traits to construct a total effect causal graph and then applies network deconvolution to the estimated (total) causal network to estimate the direct causal effect graph between two traits conditionally on the mediating effects of other traits in the graph. In the first step, the MRcML method^11^ for invalid IV screening is used to infer both causal directions. In the second step, a graph deconvolution algorithm^12^ is employed on perturbed datasets^13^ to perform accurate inference in finite samples and mitigate the effect of weak instruments. However, to construct the network of direct effects, Lin et al.^6^ rely on a critical assumption regarding the spectral radius for network deconvolution that might be violated in practice. Moreover, the total effects are decomposed into the direct effects of all possible trait pairs, including the non-significant ones. This might lead to the “dilution” of the causal effect size of significant trait pairs. Chen et al.^7^ present one- and two-sample summary-level approaches for causal network inference based on structure equation modeling that accounts for the possible presence of some invalid IVs and consider the possibility of bidirectional relationships between traits. In the two-sample approach, the methodology requires complete sample overlap of the exposures (for instance, when molecular traits from the same study are considered), but limits its applicability to more general cases of exposures derived from cohorts of different sizes. In addition, it does not incorporate underlying graph uncertainty, and causal graphical model selection is performed by thresholding adjusted *p* values. Li et al.^8^ solve the problem of identifiability of Gaussian DAGs for individual-level and Zilinskas et al.^9^ for summary-level data, respectively. They distinguish between the primary variables (traits) and intervention variables (genetic variants). To make their model identifiable, they assume (among others) that any intervention variables, called instruments, cannot intervene on multiple primary variables (exclusion restriction) and that each primary variable is intervened by at least one instrument. However, their exclusion-restriction definition does not include unobserved confounders differently from a similar condition for valid IVs in MR. This limitation has been removed in Chen et al.^10^ for Gaussian individual-level data.

Here, we contribute to the solution of this problem by taking a different “unidirectional” approach. We propose the MrDAG model, an MR method with essential graphs (EGs) learning and causal effects estimation. MrDAG uses summary-level genetic associations from genome-wide association studies (GWASs) to learn how inter-related exposures affect multiple outcomes which, in turn, are interconnected in a complex fashion. The estimated relationships within the exposures and the outcomes are then used for the estimation of the direct causal effects. MrDAG combines three causal inference strategies, the first of which is the MR paradigm, which uses genetic variation as IVs^14^^,^^15^ to ensure unconfoundedness. As such, the directionality of the causal effects between the exposures and the outcomes is assumed known, in the sense that exposures can only be potential causes of the outcomes and no reverse causation from the outcomes to the exposures is allowed. This reflects a hypothesis-driven research question that aims at understanding how certain exposures affect a set of related outcomes, and it is a plausible assumption when only the designed exposures are modifiable, i.e., an intervention can be carried out. The second strategy is structure learning,^16^ i.e., graphical models selection to define the graphs that best describe the dependency structure in a given dataset, thus accounting for graph uncertainty, under the constraint on edges’ orientation from the exposures to the outcomes. The third strategy is interventional calculus to derive principled causal effects estimates^17^ given the identified graphical models, thus shrinking to zero the causal effects of unimportant dependency relations.

Our motivating real data application considers the impact of six common modifiable lifestyle and behavioral exposures on seven mental health phenotypes. Mental health describes patterns of cognitive, emotional, and behavioral dysregulations that limit daily functioning and cause distress. One in eight individuals suffers from one or more mental health phenotypes worldwide, most commonly anxiety-, attention-deficit hyperactivity-, autism-spectrum-, bipolar-, eating-, personality-, or schizophrenia-related diseases.^18^ Collectively, they contribute to more than 15% of total years lived with disability.^19^ Clinically, mental health phenotypes are notoriously difficult to disentangle and diagnose due to the lack of objective biological biomarkers and distinct disease impressions.^20^ No symptom can be uniquely ascribed to one disease, and each disease comprises experiencing a group of inter-related traits. In research, this complexity is reinforced by the multi-faceted mechanisms that cause and sustain mental health.^20^^,^^21^ In addition to genetic liability, numerous behavioral and lifestyle factors such as alcohol consumption, smoking, sleep hygiene, physical activity, and education contribute to the risk of developing a mental health trait.^21^^,^^22^ Notably, these factors are also affected by existing disease and treatment.^23^ It is essential to appreciate these complexities when attempting to identify underlying mechanisms of mental health. While MR studies have been effective in circumventing some of the limitations of traditional epidemiology such as environmental confounding and reverse causation, MR remains largely unable to fully disentangle the interplay between traits that cause or result from mental health.^24^

**Figure 1:**
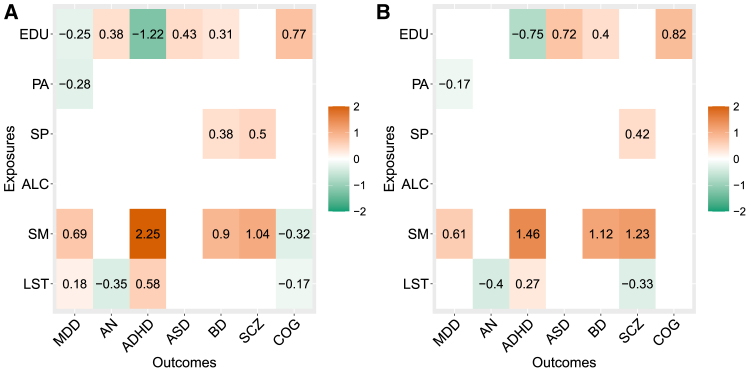
Results of standard and multi-variable MR (MVMR) methods regarding how lifestyle and behavioral exposures impact mental health outcomes (A) Total causal effects estimated by standard MR^25^ of one exposure and one outcome at a time at 5% Benjamini-Hochberg (BH) false discovery rate (FDR)^29^ across all exposures and outcomes. (B) Direct causal effects estimated by a Bayesian MVMR method (MR-BMA)^1^ after adjusting for multiple testing^30^ at 5% BH FDR across all exposures and outcomes.

To illustrate these ideas, Figure 1 shows the estimated total causal effects obtained by standard MR of one exposure and one outcome at a time^25^ and the direct effects from a multi-variable Bayesian MR model (MVMR)^1^ that accounts for the other exposures considered in the real data application. Irrespective of the method, three lifestyle and behavioral exposures seem important when considering the number of associated mental health phenotypes and the size of the causal effects: lifetime smoking index (SM), education (in years) (EDU), and leisure screen time (LST). There are a few differences between the two MR methods, in particular the role of the genetically predicted level of SM and LST on cognition (COG). However, these methods do not consider the dependency relations that might exist between lifestyle and behavioral exposures and have been already reported in MR literature, for instance, between EDU and SM and EDU and LST (EDU has a positive effect on reducing SM and LST^26^^,^^27^). Similarly, they do not model the relationships that might be present and have been detected among mental health phenotypes. For instance, bipolar disorder (BD) and anorexia nervosa (AN) might be consequences of genetic liability to major depressive disorder (MDD).^28^

In this study we show that, if these dependencies within multiple related exposures and multiple related outcomes are not considered, the results are severely biased by falsely detected causal effects (despite false discovery rate [FDR] control) and inflated effect sizes (see Tables S2 and S3). In contrast, by estimating the relationships within modifiable lifestyle and behavioral exposures and within mental health phenotypes, MrDAG provides more interpretable results regarding the direct and indirect, i.e., partially or completely meditated, effects of each exposure on the outcomes with fewer false positives and false negatives, and thus informs precise strategies for the prevention and therapeutic intervention of mental health (see the results of real data application obtained by the proposed MrDAG model in Figure 7). A detailed discussion of these results is provided in “real data application: the impact of lifestyle and behavioral traits on mental health.”

## Methods

### Causal inferential strategies in MrDAG

MrDAG combines three causal inference strategies.

First, MR has pioneered the ability to use genetic data as IVs to derive causal statements from observational data despite the presence of unobserved confounders.^31^^,^^32^

**Figure 2:**
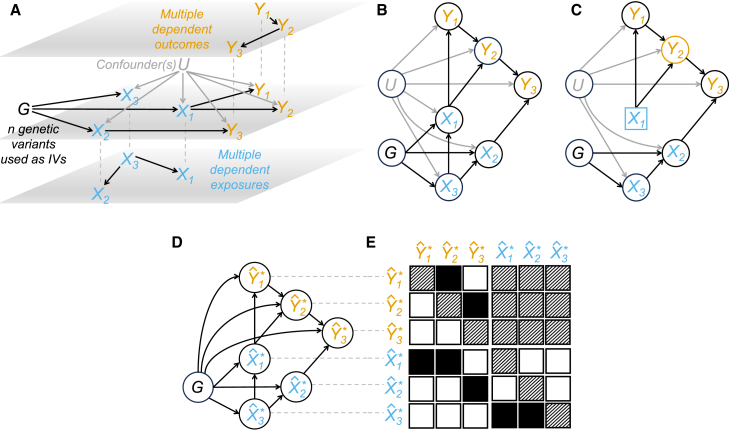
Representation of the proposed multiple exposures and multiple outcomes Mendelian randomization model and causal effects estimation where reverse causation is not allowed (A) (Middle) Multi-variable Mendelian randomization for multiple responses with $G={\left(G_{1},\ldots ,G_{n}\right)}^{⊤}$: genetic variants (black) or instrumental variables (IVs); $X={\left(X_{1},X_{2},X_{3}\right)}^{⊤}$: exposures (blue); $Y={\left(Y_{1},Y_{2},Y_{3}\right)}^{⊤}$: responses (orange); U: unobserved confounder(s) (gray). True (unconfounded by U) exposure-outcome dependency relations are depicted in the middle panel. (Bottom) True fork structure within the exposures with $X_{3}$ regarded as the common cause of $X_{1}$ and $X_{2}$. (Top) True chain structure within the outcomes, where $Y_{1}$ affects $Y_{3}$ through $Y_{2}$. (B) Directed acyclic graph (DAG) obtained by combining the panels in (A). (C) Estimation of the causal effect under intervention in $X_{1}$ on $Y_{2}$, highlighted in blue and orange, respectively. The representation of $X_{1}$ has changed to emphasize that, under intervention, it is no longer a random variable. Intervention affects only the conditional distribution of $X_{1}$, i.e., $X_{1}|\left(X_{3},G,U\right)$, and it leaves unaltered all the others. After removing the effect of $Y_{1}$ by marginalization (see Appendix A), it would be sufficient to condition on $X_{3},G$, and U (graphically, the directed edges to $X_{1}$ from $X_{3},G$, and U are removed) to guarantee that the association between $X_{1}$ and $Y_{2}$ is purely causative (see Figure S1). However, since U is unobserved, the estimation of the causal effects cannot be obtained only by conditioning. (D) Genetically predicted exposures ${\hat{X}}^{*}={\left({\hat{X}}_{1}^{*},{\hat{X}}_{2}^{*},{\hat{X}}_{3}^{*}\right)}^{⊤}$ and outcomes ${\hat{Y}}^{*}={\left({\hat{Y}}_{1}^{*},{\hat{Y}}_{2}^{*},{\hat{Y}}_{3}^{*}\right)}^{⊤}$ depend only on G, which are chosen to be associated with X and not with Y. Graphically, no directed edges to ${\hat{X}}^{*}$ and ${\hat{Y}}^{*}$ from U are pictured. True (unconfounded by U) dependency relations between the traits in the original (individual-level) data shown in (B) are obtained by using ${\hat{X}}^{*}$ and ${\hat{Y}}^{*}$. (E) Adjacency matrix describing the Markov properties of the DAG involving the genetically predicted exposures and outcomes (the variables in the x axis are dependent on the variables in the y axis) that are function of the IVs and the inverse-variance weighting (IVW) (depicted with an asterisk) summary-level statistics ${\hat{B}}_{X}^{*}={\left({\hat{\beta }}_{X_{1}}^{*},{\hat{\beta }}_{X_{2}}^{*},{\hat{\beta }}_{X_{3}}^{*}\right)}^{⊤}$ and ${\hat{B}}_{Y}^{*}={\left({\hat{\beta }}_{Y_{1}}^{*},{\hat{\beta }}_{Y_{2}}^{*},{\hat{\beta }}_{Y_{3}}^{*}\right)}^{⊤}$. Neither reverse causation (top-right submatrix) nor phenotypic traits feedback loops (main diagonal) are allowed. Color code: black, directed edge between variables; white, no causal relationship between variables; black-white strips, directed edge not allowed (feedback loop and reverse causation between exposures and outcomes).

Second, in its standard formulation of one exposure and one outcome, the conditional dependencies between the outcome Y, the exposure X, the IV G, and the unobserved confounder U are all given as well as their graphical representation.^15^ When multiple exposures X^1^ and multiple outcomes Y^2^ are considered along with multiple IVs G, (partial) correlation between X and conditional dependencies between Y are included in the models to perform the selection of important exposures whose causal effects can be shared or are distinct across the responses. However, no dependency relations within the exposures and the outcomes are estimated by these methods, although, in practical applications, the effect of an exposure on an outcome can be confounded or (partially or completely) mediated by another exposure or mediated by another outcome, or both (see Figures 2A and 2B for an illustration).

In real data applications, complex dependency relations between the traits are generally not known in advance and they need to be learned from the data. To detect them, we rely on EGs and structure learning. Graphical models are multi-variate distributions associated with a graph and are very effective for encoding conditional dependencies^33^ between random variables. They are represented in a graph as nodes (vertices), while edges denote conditional dependence relationships between the corresponding random variables. A DAG is a directed graph, where each edge has an orientation with no directed cycles. Structure learning is a model selection problem^16^ to estimate the graph (or competing graphs) that best describes the dependency structure in a given dataset. However, without identifiability conditions,^8^^,^^9^^,^^10^^,^^34^ it is not possible to estimate uniquely the underlying DAG, since its conditional independencies can be associated with several alternative DAGs. The set of DAGs that hold the same conditional independencies is known as Markov equivalent class (MEC), and the best that can be done from observational data is to estimate this class (or competing classes). Moreover, all DAGs with the same conditional independencies can be represented by an EG.^35^ Thus, this study aims to illustrate how to perform EG learning (and thus the exploration of distinct DAGs in the identified EGs whose importance will be apparent in the next paragraph) that best fit the data under the constraint on the orientation of the edges, known as partial ordering,^36^ from the exposures to the outcomes implied by the MR paradigm.

Third, along with the identification of the exposure-outcome relations as well as the dependency patterns within the exposures and the outcomes, we are also interested in causal effects estimation under intervention.^17^ Intervention has to be interpreted as a manipulation of an exposure to be forced to take a particular value (“doing”) in contrast to the natural value that can be observed (“seeing”).^37^ This objective is possible, since graphical models based on DAGs are suited for causal reasoning based on the notion of interventional distribution^17^ (see Appendix A for details). An intervention on the exposures can be made explicit by a suitable modification of the multi-variate distribution associated with the DAG, under the assumption that the intervention does not affect any other variable in the joint distribution besides the conditional distribution of the exposure under intervention.^38^ DAGs in which it is possible to perform an intervention on any arbitrary node are called causal DAGs.^37^ After intervention, it is possible to use graphical rules to convert the conditioning on “doing” (intervention) into conditioning on “seeing” (observation), derive the interventional distribution, and finally estimate causal effects.^17^ Figure 2C presents an example of the intervention on an exposure and the estimation of the causal effect on an outcome.

In the formulation described above, all confounders should be measurable to perform structure learning and causal effects estimation (causal sufficiency assumption^39^). This condition is (explicitly or implicitly) assumed^4^^,^^5^^,^^8^^,^^9^ and is usually not met in real data applications where, instead, unobserved confounders are ubiquitous and affect exposures and responses at the same time. To solve this problem, we demonstrate (see Appendix A) and show in an extensive simulation study (see Results) that, under partial ordering, we can estimate the dependency structure that exists between the traits in the original (individual-level) data unconfounded by U by using their genetically predicted values. Since the genetically predicted traits depend only on the selected IVs, the confounders do not mask the true dependency relations required in causal effects estimation. See Figure 2D, where the graphical model estimated by using genetically predicted exposures and outcomes approximates the corresponding graph in the individual-level data not affected by U. Our proposed approach shares similarities with methods based on the genetic correlation and developed to analyze the joint genetic architecture of complex traits,^40^ where the genetically predicted exposures and outcomes can be seen as the estimated genetic components of the traits. We use this analogy to show the unconfoundedness of the estimated dependency structure that exists between the traits. Finally, for a given DAG in the identified EGs, the genetically predicted values of the exposures and the outcomes are used to derive the causal effect estimator that complies with Pearl’s back-door criterion,^17^ which indicates the variables that must be added to the regression equation to eliminate what is known as “omitted variable bias.”

The MrDAG model can be summarized as follows:

(Equation 1) $${\left[g^{⊤}{\hat{B}}_{Y}^{*}g^{⊤}{\hat{B}}_{X}^{*}\right]}^{⊤}∼N_{q+p}\left({\left[g^{⊤}B_{Y}^{*}g^{⊤}B_{X}^{*}\right]}^{⊤},\Sigma^{*}\right)\text{,}$$

where g are the IVs after pruning or clumping, ${\hat{B}}_{Y}^{*}$ and ${\hat{B}}_{X}^{*}$ are the inverse-variance weighted (IVW)^41^ estimated genetic associations with the outcomes and the exposures, ${\hat{Y}}^{*}=g^{⊤}{\hat{B}}_{Y}^{*}$ and ${\hat{X}}^{*}=g^{⊤}{\hat{B}}_{X}^{*}$ are the genetically predicted values of the outcomes and exposures based on the IVs (see Figure 2D), which are normally distributed for large sample sizes, and $\Sigma^{*}$ is the covariance matrix that can be partitioned into $\Sigma_{YY}^{*},\Sigma_{XX}^{*}$, and $\Sigma_{XY}^{*}$, the genetic covariances within the outcomes, the exposures, and between them. Note that the genetically predicted values of the outcomes and exposures do not need to be available/calculated, since the proposed causal graphical model uses as input data the sufficient statistic for $\Sigma^{*}$, which is a function of the summary-level data and the linkage disequilibrium (LD) structure between the genetic variants selected as IVs. Thus, the only additional information that is required from individual-level data is the LD matrix V. However, this information is not necessary when independent genetic variants are considered after pruning or clumping, as we have done in the simulation study and the real data application, and, thus, $V=I_{n}$ (see Appendix A).

By using a summary-level MR design, the MrDAG model allows us to find a solution to the two problems highlighted before. First, we perform structure learning under partial ordering by using $\Omega^{*}=\Sigma^{{*}^{-1}}$ to learn the unconfounded dependency relations within the exposures, the outcomes, and between them and to understand the genetic paths that link exposures and outcomes. Second, we estimate the causal effects of the intervention on the exposures as a function of trait-specific elements of the genetic associations ${\hat{B}}_{Y}^{*}$ and ${\hat{B}}_{X}^{*}$ informed by distinct DAGs in the identified EGs, unconfounded by any measured and unmeasured pleiotropic effects^42^ within the exposures and the outcomes, respectively, and any unobserved confounder.

Finally, the uncertainty regarding which EGs best describe the data is fully accounted for in the Bayesian implementation of the proposed model (see Appendix A). Posterior inference allows us to rank the identified graphical models according to their importance and to obtain causal effects by Bayesian model averaging, which shows advantages compared to frequentist approaches.^43^ Sparsity to detect important causal effects is obtained by specifying the *a priori* number of edges (or its probability) in the graphical model, which is easier to elicit than Lasso-type penalization on the space of causal effects used in frequentist approaches.^44^

### Selection of instrumental variables

MrDAG uses the same instrument selection procedure employed in MVMR.^1^ A genetic variant is considered a valid instrument for MVMR when three core conditions hold.^3^ (IV1) Independence: the variant is independent of all confounders of each of the exposure-outcome associations. (IV2) Relevance: the variant must not be conditional independent of each exposure given the other exposures. (IV3) Exclusion restriction: The variant is independent of the outcome conditional on the exposures and confounders. We revise these core conditions for multiple exposures and extend them for multiple outcomes in the Appendix A. In practice, only IV2 can be computationally evaluated from the available data. Tests for weak IV bias that arises because some genetic variants are weakly associated, with some exposures conditional on the other exposures are available.^45^ A recent solution to mitigate the effects of weak IVs in MVMR is presented in Wu et al.^46^

There is an important distinction between IV selection in MVMR, as used by MrDAG, and bidirectional MR. Let us consider two traits A and B. In bidirectional MR, two MR analyses are conducted, one for trait A on trait B and vice versa. First, specific IVs are selected for trait A and the first MR model is fit. Another set of specific IVs is then selected for trait B, and the second MR model tests the opposite effects direction. In contrast, in MVMR, IVs are chosen to be the union of genome-wide significant genetic variants for any exposure. By combining MVMR IV selection approach with EG learning, MrDAG can infer the bidirectionality of the relationships within exposures based on $\Omega_{XX}^{*}=\Sigma_{XX}^{{*}^{-1}}$ without repeated IV selection and subsequent analyses. A similar comment can be made for the estimation of the bidirectionality of the relationships within the outcomes based on $\Omega_{YY}^{*}={\left(\Sigma_{YY}^{*}-\Sigma_{YX}^{*}\Sigma_{XX}^{{*}^{-1}}\Sigma_{XY}^{*}\right)}^{-1}$ (see Appendix A). The dependencies within the outcomes can be interpreted as an indication of a violation of condition IV3, i.e., pleiotropy not explained by the estimated causal effects from the exposures to the outcomes.^2^ The detected relationships within the exposures also suggest the existence of pleiotropy, which, in the proposed framework, comprises confounding, mediation and independent pleiotropic pathways.^3^ In this study, measured pleiotropy is accounted for via any of the exposures included in the model.^3^ Unmeasured pleiotropy, in contrast, is accounted for when an unmeasured pathway impacts more than one outcome jointly and introduces unidirectional and/or bidirectional effects among the genetic associations of the outcomes.^2^

While MrDAG can account for the impact of these pleiotropic effects, other direct effects of some IVs on the outcomes might exist and bias the results. Indeed, another unmeasured pleiotropy might be present in which some genetic variants are directly associated with a single outcome at a time and not via the exposures. To deal with this scenario, a possible extension of MrDAG model in Equation 1 is in analogy with MR-Egger for MVMR,^42^ where an intercept is added. However, the InSIDE assumption must be imposed, and its violation can cause further bias.^47^ Instead of extending the MrDAG model in this direction, an alternative strategy is to check whether any of the genetic variants used as IVs do not follow the proposed model. This is equivalent to detecting outliers as proposed for univariable^48^ and multi-variable MR models^3^ to identify specific IVs that might be invalid due to a direct unmeasured pleiotropic pathway impacting one outcome at a time. This approach, called conditional predictive ordinate (CPO), has already been pursued in a Bayesian multi-response MR model^2^ and is also included in MrDAG. Details are presented in the Appendix A.

Overall, only the direction from exposures to outcomes is fixed in MrDAG, and no reverse causation is allowed, reflecting the standard MR paradigm. Thus, one of the key design decisions for MrDAG is which variables are considered exposures and, consequently, which instruments are selected for these exposures.

## Results

### Simulation study

We compare MrDAG in a comprehensive simulation study where four different *in silico* scenarios have been generated on individual-level data for N=100,000 individuals with $N_{Y}=N_{X}=50,000$. The simulated datasets include n=100 independent genetic variants G, an unobserved confounder U, 15 exposures X, and 5 outcomes Y. All exposures X were measured on the same individuals in the first sample and have complete overlap, and all outcomes Y were measured on the same individuals in the second sample independent of the first sample. In all simulations, the unconfounded dependency relations between the traits are simulated at the individual level, while the algorithms use as input data the corresponding IVW summary-level statistics.

The four simulation scenarios are built by combining two different strategies we used to simulate the dependency patterns within the exposures and the responses.

**Figure 3:**
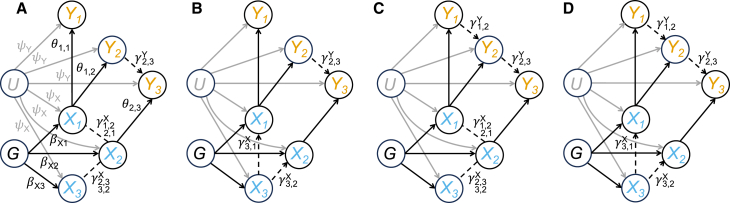
Schematic illustration of different dependency structures simulated between the traits at the individual-level data and the parameters employed in the simulation study Directed edges indicate dependency relations, while undirected edges denote partial correlations. Dashed lines depict the true (unconfounded by U) dependency structure within the exposures and the outcomes, while solid lines indicate true causal effects between them. Parameters $\psi_{Y}$ and $\psi_{X}$ indicate the simulated effects of the unobserved confounder U on the exposures and the outcomes, respectively, and $B_{X}=\left(\beta_{X_{1}},\beta_{X_{2}},\beta_{X_{3}}\right)$ are the simulated genetic effects on the exposures. For simplicity, they are shown only on the left panel. $\Theta =\left(\theta_{1,1},\theta_{1,2},\theta_{2,3}\right)$ are the simulated causal effects from the exposures to the outcomes, while $\Gamma_{X}=\left(\gamma_{3,1}^{X},\gamma_{3,2}^{X}\right)$ and $\Gamma_{Y}=\left(\gamma_{1,2}^{Y},\gamma_{2,3}^{Y}\right)$ are the mediation parameters within the exposures and the outcomes, respectively, where the subscripts denote their directionality. When partial correlations are simulated within the exposures, bidirectional effects are depicted with double subscripts, i.e., $\Gamma_{X}=\left(\gamma_{1,2//2,1}^{X},\gamma_{2,3//3,2}^{X}\right)$. (A) Simulated scenario “${\text{UndG}}_{X}$-${\text{Med}}_{Y}$,” where an undirected graph (“${\text{UndG}}_{X}$”) encodes the dependency pattern within X and, within the responses, an outcome $\left(Y_{3}\right)$ is completed mediated (“${\text{Med}}_{Y}$”) by another response $\left(Y_{2}\right)$, which, in turn, is affected by a different exposure $\left(X_{1}\right)$. Although there is another partial mediation between $X_{1}$ and $Y_{3}$ through $X_{2}$, this mediation happens within X, so it does not affect the definition of complete mediation within Y. (B) Simulated scenario “${\text{DAG}}_{X}$-${\text{Med}}_{Y}$,” where a topologically ordered DAG within the exposures (“${\text{DAG}}_{X}$”) is simulated. Specifically, in the example depicted, a fork structure is simulated, i.e., $X_{3}$ affects both $X_{1}$ and $X_{2}$. A complete mediation is still considered within the responses. (C) Simulated scenario “${\text{UndG}}_{X}$-${\text{DAG}}_{Y}$.” Here, the dependency structure between the individual-level responses is obtained by simulating a topologically ordered DAG (“${\text{DAG}}_{Y}$”). Specifically, a chain structure is considered, i.e., $Y_{1}$ affects $Y_{2}$, which, in turn, affects $Y_{3}$, whereas an undirected graph encodes the dependency pattern within X. (D) Simulated scenario “${\text{DAG}}_{X}$-${\text{DAG}}_{Y}$,” where two topologically ordered DAGs are simulated within the exposures (fork structure) and outcomes (chain structure), respectively.
(2) “${\text{DAG}}_{X}$-${\text{Med}}_{Y}$.” The dependency relations within the exposures are more complex than in scenario (1), since a topologically ordered DAG within the exposures (“${\text{DAG}}_{X}$”) is simulated.^50^ A complete mediation is still considered within the responses. This second scenario is illustrated in Figure 3B.
(3) “${\text{UndG}}_{X}$-${\text{DAG}}_{Y}$.” Here, a more complex dependency structure within the individual-level responses (“${\text{DAG}}_{Y}$”) is simulated. This scenario is represented in Figure 3C. An example of the complex dependency patterns generated in the simulation study between the traits for one replicate of scenario ${\text{UndG}}_{X}$-${\text{DAG}}_{Y}$ is shown in Figure 4A.

(1) “${\text{UndG}}_{X}$-${\text{Med}}_{Y}$.” A sparse undirected graphical model (“${\text{UndG}}_{X}$”) encodes the dependency pattern within the exposures $X=\left(X_{1},\ldots ,X_{15}\right)$. Regarding the responses $Y=\left(Y_{1},\ldots ,Y_{5}\right)$, one outcome is completed mediated by another one (“${\text{Med}}_{Y}$”). This simulated scenario aims to assess the ability of MrDAG to detect the most common type of relationship within the exposures assumed in MVMR methods.^1^^,^^3^^,^^49^ For a visual representation of this scenario, see Figure 3A.

**Figure 4:**
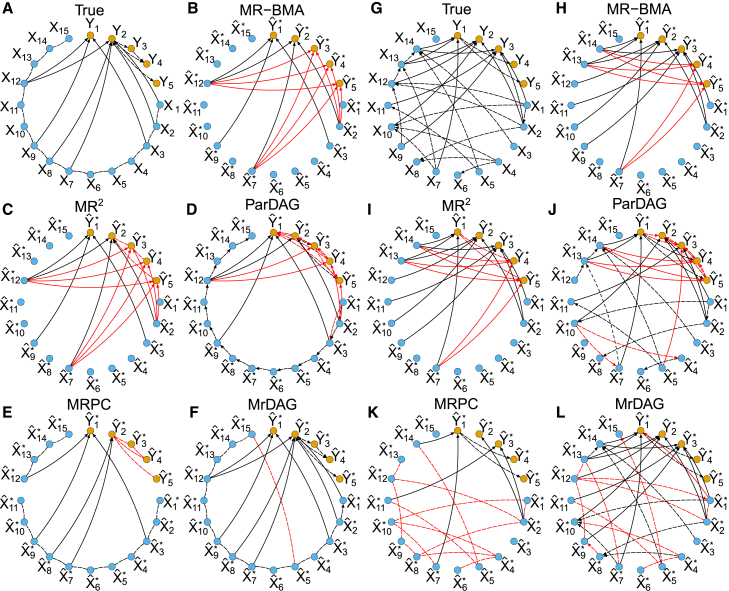
Examples of unconfounded dependency structure simulated at the individual-level data and estimated by using summary-level statistics within the exposures, the outcomes, and between them in two different scenarios In each panel, individual-level outcomes $Y=\left(Y_{1},\ldots ,Y_{5}\right)$ and exposures $X=\left(X_{1},\ldots ,X_{15}\right)$ as well as genetically predicted outcomes ${\hat{Y}}^{*}=\left({\hat{Y}}_{1}^{*},\ldots ,{\hat{Y}}_{5}^{*}\right)$ and exposures ${\hat{X}}^{*}=\left({\hat{X}}_{1}^{*},\ldots ,{\hat{X}}_{15}^{*}\right)$ are represented with orange and blue nodes, respectively. Directed edges indicate dependency relations, while undirected edges denote partial correlation. Dashed lines depict the true (unconfounded by U) and estimated dependency structure within the exposures and the outcomes, while solid lines indicate true and estimated causal effects between them. Red color denotes false positives, either falsely detected effects (regardless of the directionality) or wrong directionality of the edges. Besides the proposed model, alternative methods considered Mendelian randomization with Bayesian model averaging (MR-BMA),^1^ multi-response Mendelian randomization (${\text{MR}}^{2}$),^2^ Mendelian randomization with PC algorithm (MRPC),^51^ and partition-DAG (ParDAG).^44^ We report the results of MR-BMA and ${\text{MR}}^{2}$ obtained by thresholding the marginal posterior probability of inclusion (mPPI) >0.5, which correspond to the median models.^52^ No threshold is applied to MrDAG posterior probability of edge inclusion (PPEI). MRPC partially directed acyclic graphs (PDAGs) are obtained by specifying the type I error rate for the conditional independence test at α=0.01. ParDAG results are the solutions of causal effects estimation with Lasso penalization set at λ=0.9. (A–F) Single replicate of the simulated scenario ${\text{UndG}}_{X}$-${\text{DAG}}_{Y}$, where an undirected graph encodes the dependency pattern within X and a DAG represents the dependency relations within Y along with the simulated causal effects from the exposures to the outcomes, resulting in an overall partially oriented DAG. In this scenario, the strength of correlation between consecutive X is set at $r_{X}=0.6$ and then decreases exponentially for non-consecutive exposures, and the average level of the mediation parameters within Y is set at $m_{Y}=1$. (G–L) Single replicate of the simulated scenario ${\text{DAG}}_{X}$-${\text{DAG}}_{Y}$, where two topologically ordered DAGs have been independently simulated within X and Y along with the simulated causal effects from the exposures to the responses, resulting in an overall fully oriented DAG. In this scenario, the average level of mediation parameters for X and Y are set at $r_{X}=0.6$ and $m_{Y}=1$, respectively.
(4) “${\text{DAG}}_{X}$-${\text{DAG}}_{Y}$.” This is the most complex simulated scenario, where two independent topologically ordered DAGs have been simulated within the exposures and outcomes. Figure 3D presents a schematic illustration of this scenario, while Figure 4G shows the intricate dependency structure simulated between the traits for one replicate of ${\text{DAG}}_{X}$-${\text{DAG}}_{Y}$ scenario.

Taken together, in scenarios (2) and (4), the overall individual-level DAGs obtained by combining two different simulation strategies for X and Y are fully oriented, while in scenarios (1) and (3) the overall DAGs are partially oriented. Details regarding the parameters $\psi_{X}$ and $\psi_{Y}$, the simulated levels of the effects of the unobserved confounder U on the responses and the outcomes, $B_{X}$, the simulated levels of the genetic effects on the exposures, and $\Gamma_{X}$ and $\Gamma_{Y}$, the simulated levels of the mediation parameters within the exposures and the outcomes and their average value $r_{X}$ and $m_{Y}$, are presented in the Appendix A. Finally, all simulations are replicated 25 times and initialized with a different random seed.

We compare MrDAG with published MVMR methods and their software implementations, excluding the comparisons from naive one-exposure one-outcome MR models, since it has been shown that they are outperformed by MVMR methods when there is measured pleiotropy among exposures.^3^ Specifically, we consider MR with Bayesian model averaging (MR-BMA),^1^ an MVMR algorithm that allows for many exposures to be included but does not model explicitly the dependency relations within the exposures.^3^ MR-BMA estimates the sparse direct causal effects between the exposures and one outcome, providing the marginal posterior probability of inclusion (mPPI) along with the (Bayesian model-averaged) direct causal effects. We treat MR-BMA as the baseline algorithm for the comparisons, since it analyzes one outcome at a time. Second, we present the results of a sparse multi-variable Bayesian summary-level MR model for the joint analysis of multiple responses (${\text{MR}}^{2}$).^2^ ${\text{MR}}^{2}$ estimates mPPIs, the (posterior mean of) direct causal effects between the exposures and the outcomes as well as the residual covariation between the outcomes not explained by the exposures. Similarly to MR-BMA, it allows for correlation among the exposures, while unmeasured pleiotropy between responses is accounted for by a Gaussian decomposable graphical model. Third, we include an in-house modified version for summary-level data of the PC algorithm^53^ with the principle of MR (MRPC),^51^ which uses the PC algorithm for the estimation of the causal graphical model among the variables (in the original implementation: genetic variants and traits; in our modified version: exposures and outcomes) under partial ordering. At a specified type I error rate for the Gaussian conditional independence test, MRPC returns the estimated partially directed acyclic graphs (PDAGs)^36^ (see Appendix A) in which some undirected edges are present along with the directed ones (recall that an undirected edge z−v is equivalent to z→v and v→z) as well as the *p* values of all conditional independence tests. For a given PDAG detected by MRPC in each replicate and scenario, we utilize Kalisch et al.^50^ to estimate the causal effects between the exposures and outcomes. Fourth, Partition-DAG (ParDAG)^44^ provides a solution to the structure learning problem once the summary-level statistics have been partitioned into two groups and the orientation of the edges from the exposures to the outcomes has been enforced. ParDAG computes the causal effects estimates under Lasso regularization. It has not been combined with instrumental variable estimation and applied to genetic data to date. Finally, we consider Graph-MRcML,^6^ which is based on a bidirectional MR framework and does not distinguish between exposures and outcomes. Among the causal graphical models considered, ParDAG is the only one that returns the estimation of a fully oriented DAG, while MRPC, Graph-MRcML, and MrDAG return PDAGs according to the designed level of type I error rate, Bonferroni-adjusted significance level, and posterior probability of edge inclusion (PPEI), respectively. However, since in ParDAG no identifiability conditions are assumed, the reported DAG is the sparsest DAG in the MEC. All methods use summary-level statistics as input data after IVW. For each method and algorithmic implementation, details of the parameter settings are provided in supplemental text. Finally, all algorithms were run on the same Cambridge high-performance computer (HPC), taking for each replicate (and based on the designed parameters settings) on average 1 min for MR-BMA and MRPC, 2 min for ${\text{MR}}^{2}$, <10 s for ParDAG, >2 h for Graph-MRcML, and 10 min for MrDAG.

Regarding the evaluation criteria, we use a precision-recall curve (PRC) that shows the relationship between precision (i.e., positive predictive value, on the y axis) and recall (i.e., sensitivity, on the x axis) for every possible cutoff and is not impacted by the over-representation of null effects. In drawing PRCs, for MR-BMA, ${\text{MR}}^{2}$, and MrDAG, we rank the estimated mPPIs and PPEIs, respectively. For Graph-MRcML, we rank p values obtained by the perturbation scheme. This allows us to represent the PRC as a smooth step line, where each step corresponds to a different cutoff on mPPIs, PPEIs, and p values. As ParDAG is based on a Lasso-type penalization, unimportant causal effects are forced to zero and excluded from the model. Consequently, ParDAG does not provide a full ranking of important dependency relations but rather a single cutoff. Thus, it is presented as a single point along with its standard error for each specified value of the penalization parameter λ instead of a continuous line. Similarly, we report as a single cutoff the PDAG estimated by MRPC in two steps (graph-skeleton selection at a specified α followed by edges-orientation step with further conditional independence tests at the same type I error rate) for different values of α. See supplemental text for a detailed discussion regarding how we implemented a fair comparison between the methods considered.

Finally, to evaluate the quality of the causal effects estimation, we calculate the sum of squared errors (SSE), defined as the sum of the squared differences between the estimated and the simulated causal effect. In contrast to the evaluation of the recovery obtained by each method of the simulated dependencies within the exposures, the outcomes, and between them, we do not report the SSE of the mediation parameters $\Gamma_{X}$ and $\Gamma_{Y}$, since they are considered nuisance parameters in the proposed model (see supplemental text).

### MrDAG more accurately detects unconfounded dependency relations within the exposures and the outcomes and between them

Figure 4 presents the results of MrDAG and alternative methods for one replicate of the simulated scenario ${\text{UndG}}_{X}$-${\text{DAG}}_{Y}$ (Figures 4A–4F) and ${\text{DAG}}_{X}$-${\text{DAG}}_{Y}$ (Figures 4G–4L) for a particular choice of the parameters $r_{X}=0.6$ and $m_{Y}=1$ used in the simulation study to control the average value of the mediation parameters $\Gamma_{X}$ within the exposures and $\Gamma_{Y}$ within the outcomes, and $\psi_{X}=2$ and $\psi_{Y}=1$ for the level of confounding on the exposures and the outcomes, respectively (see Appendix A).

For its applicability, Graph-MRcML requires the assumption that the spectral radius of the direct causal graph is less than 1,^6^^,^^7^^,^^9^ which is violated not only in these two replicates but in most of the simulated scenarios’ replicates. Therefore, although originally considered, we omit Graph-MRcML from the simulation study. The general performance of the other competing algorithms is already apparent from it. In scenario ${\text{UndG}}_{X}$-${\text{DAG}}_{Y}$, if a causal effect is simulated from an exposure to an outcome and there are dependency relations from this outcome to other responses (Figure 4A), MR-BMA adds erroneously causal effects to all linked responses with severe false positive (FP) inflation (Figure 4B, FPs between ${\hat{X}}_{12}^{*}$ and ${\hat{Y}}_{3}^{*},{\hat{Y}}_{4}^{*},{\hat{Y}}_{5}^{*}$ depicted in red). On the other hand, MR-BMA estimates neither the dependency pattern within X, since the (partial) correlation between summary-level exposures is assumed in the model^3^ but not estimated, nor the dependencies within Y, since it considers one response at a time. MR^2^ detects bidirectional (as assumed by the model) relationships between the outcomes, although this is not sufficient to prevent FPs, similarly to MR-BMA (Figure 4C). ParDAG results regarding the causal effects simulated from the exposures to the outcomes are extremely sparse but very dense within the responses. Only oriented dependencies are estimated within the responses, as assumed by the model (Figure 4D). MRPC infers correctly most of the dependencies within X, but it does not have the power to detect all simulated causal effects Θ at the specified type I error rate for the conditional independence test (α=0.01) with a few false negatives (FNs) (Figure 4E, FNs between ${\hat{X}}_{1}^{*},{\hat{X}}_{2}^{*}$ and ${\hat{Y}}_{2}^{*}$) and well as FPs within Y (FPs between ${\hat{Y}}_{2}^{*},{\hat{Y}}_{3}^{*},{\hat{Y}}_{4}^{*},{\hat{Y}}_{5}^{*}$, where bidirectionally is erroneously detected). MrDAG performs better than alternative methods to detect both directed and bidirected edges, with only one FP between ${\hat{X}}_{5}^{*}$ and ${\hat{X}}_{15}^{*}$ (Figure 4F).

Similar comments can be made for a particular replicate of scenario ${\text{DAG}}_{X}$-${\text{DAG}}_{Y}$, although in this scenario the dependency patterns are more complex, since a topological ordered DAG is simulated also within the outcomes (Figure 4G). ${\text{MR}}^{2}$ does not detect any unmeasured pleiotropy within the outcomes, and the results coincide with MR-BMA, both with several FPs (Figures 4H and 4I). MrDAG confirms its good performance except for the directionality of the dependency relations within X, where bidirectional edges are found with a few FPs (Figure 4L, FPs between ${\hat{X}}_{12}^{*}$ and ${\hat{X}}_{1}^{*}$ and between ${\hat{X}}_{8}^{*}$ and ${\hat{X}}_{9}^{*}$) and an FP between ${\hat{X}}_{1}^{*}$ and ${\hat{Y}}_{1}^{*}$ (although no threshold has been applied to PPEIs) and no FNs.

**Figure 5:**
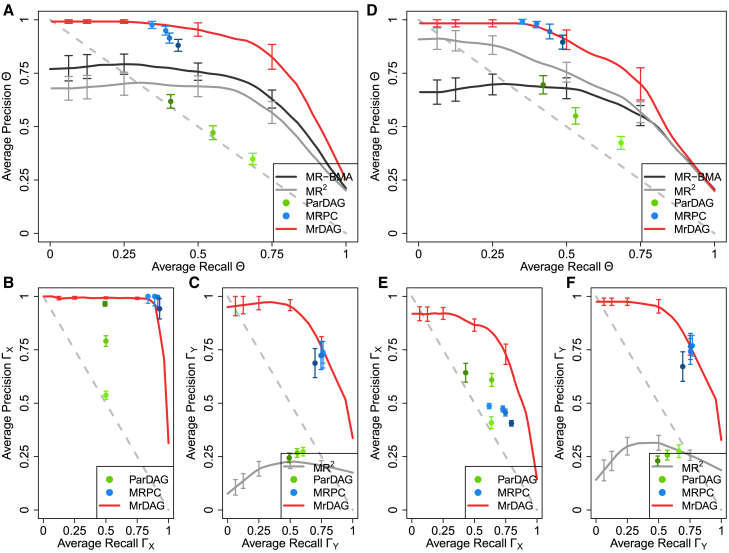
Precision-recall curves (PRCs) for all methods considered in the simulated scenarios ${\text{UndG}}_{X}$-${\text{DAG}}_{Y}$ and ${\text{DAG}}_{X}$-${\text{DAG}}_{Y}$ show recall (=sensitivity=TP/(TP+FN)) in the x-axis and precision (=positivepredictivevalue=TP/(TP+FP)) in the y-axis with TP=truepositive,FN=falsenegative and FN=falsepositive averaged over 25 replicates in each scenario. In scenario ${\text{UndG}}_{X}$-${\text{DAG}}_{Y}$ (A–C), the strength of correlation between consecutive X is set at $r_{X}=0.6$ and then decreases exponentially for non-consecutive exposures, and the average level of the mediation parameters within Y is set at $m_{Y}=1$, while in scenario ${\text{DAG}}_{X}$-${\text{DAG}}_{Y}$ (D–F), the average level of the mediation parameters within X and Y is set at $r_{X}=0.6$ and $m_{Y}=1$, respectively. For details, see Appendix A. In both scenarios, the results are presented separately for the simulated dependency structures from the exposures to the outcomes (A and D), within the exposures (B and E) and the outcomes (C and D), respectively. Vertical bars in each PRC, at specific recall levels 0.0625, 0.125, 0.25, 0.50, and 0.75, indicate standard error. For the MRPC algorithm, the type I error rate for the conditional independence test is set at α={0.01,0.05,0.10,0.20} (from light- to dark-blue dots), and for the ParDAG algorithm we specify three different values for the Lasso penalization λ={0.5,0.7,0.9} (from light- to dark-green dots). See supplemental text for details.

Figure 5 generalizes the results depicted in Figure 4, averaging the results over 25 replicates of the simulated scenarios ${\text{UndG}}_{X}$-${\text{DAG}}_{Y}$ (Figures 5A–5C) and ${\text{DAG}}_{X}$-${\text{DAG}}_{Y}$ (Figures 5D–5F) with the same parameters setting used in Figure 4. The results are presented separately for the simulated dependency structures from the exposures to the outcomes (Figures 5A and 5D), within the exposures (Figures 5B and 5E), and within the outcomes (Figures 5C and 5F), respectively.

On average, MRPC and MrDAG have good performance in both simulated scenarios (Figures 5A and 5D). MRPC’s best results are obtained at a stringent type I error rate α=0.01 for the conditional independent tests (blue dots), although they are quite similar across different values of α and thus robust to this choice. However, it fails to detect the simulated dependency pattern within X in scenario ${\text{DAG}}_{X}$-${\text{DAG}}_{Y}$ (Figure 5E). The performance of MR-BMA can be only evaluated for the detection of the causal effects from the exposures to the outcomes (Figures 5A and 5D). As we noticed above, the large number of FPs degrades the results of this method, which was not developed to deal with multiple related responses. MR^2^ is not able to detect complex dependency relations simulated within the outcomes (Figures 5C and 5F), although this is expected given the assumed bidirectionality within Y. It performs better than MR-BMA in scenario ${\text{DAG}}_{X}$-${\text{DAG}}_{Y}$ but not in scenario ${\text{UndG}}_{X}$-${\text{DAG}}_{Y}$, where dependencies within Y are wrongly estimated, showing that its results crucially depends on the quality of the detected unmeasured pleiotropy. The performance of ParDAG is the worst among the methods considered for all types of designed relationships, slightly better within the exposures (Figures 5B and 5E) and between the exposures and outcomes (Figures 5A and 5D), and worse within the outcomes (Figures 5C and 5F), likely due to the very dense solutions within Y as already noted in Figures 4D and 4J. Since ParDAG detects only directed edges, in Figure 5B, where the partial correlation between exposures is simulated, the method has 50% recall rate. The results also seem quite different according to the penalty parameter λ.

MrDAG has a strong performance in both scenarios. In contrast to MR-BMA and ${\text{MR}}^{2}$, in scenario ${\text{DAG}}_{X}$-${\text{DAG}}_{Y}$ (Figures 5D–5F), there is only a small reduction of the precision in the estimation of the dependency relations between the exposures and the outcomes and within the latter, compared to the scenario ${\text{UndG}}_{X}$-${\text{DAG}}_{Y}$ (Figures 5A–5C).

The comments above can be extended to the scenarios where the relationships within outcomes are completely mediated (${\text{UndG}}_{X}$-${\text{Med}}_{Y}$ depicted in Figures S2A–S2C and ${\text{DAG}}_{X}$-${\text{Med}}_{Y}$ shown in Figures S2D–S2F). In these scenarios, the mediation within the outcomes is easier to detect (Figures S2C and S2F) than a topologically ordered DAG simulated within Y.

Figure S4 shows the results of the area under the curve of precision recall (AUCPR) to detect the causal effects Θ and the sensitivity of the methods to different specifications of $r_{X}$ and $m_{Y}$. MrDAG is confirmed to be the best method, with stable AUCPR for any combination of $r_{X}$ and $m_{Y}$ and with similar AUCPR when partial correlation or a topological ordered DAG is simulated within X. MR-BMA performs well, especially in the scenario ${\text{UndG}}_{X}$-${\text{Med}}_{Y}$ (Figure S4A), which is the scenario that is most compatible for this method, as well as in scenario ${\text{DAG}}_{X}$-${\text{Med}}_{Y}$ (Figure S4C), where its performance slightly decreases. Despite the limitations highlighted above, ${\text{MR}}^{2}$ is overall the second-best method, although it shows a drop of power in scenario ${\text{UndG}}_{X}$-${\text{DAG}}_{Y}$ (Figure S4B). Both MRPC and ParDAG seem to be less precise at higher levels of $r_{X}$ irrespective of the simulated scenario, with ParDAG also influenced by the value of $m_{Y}$. Similarly, Figures S5 and S6 show the sensitivity of the algorithms to detect the simulated patterns within X and within Y for different specifications of $r_{X}$ and $m_{Y}$.

In summary, MrDAG outperforms competing methods in estimating the dependency relations, unconfounded by U, within the exposures, within the outcomes, and between them, simulated at the individual level and estimated by using summary-level data in a variety of scenarios and with different levels of the strength of the dependencies (mediation parameters $r_{X}$ and $m_{Y}$) within the exposures and the outcomes.

### MrDAG improves the estimation of the causal effects over existing methods

**Figure 6:**
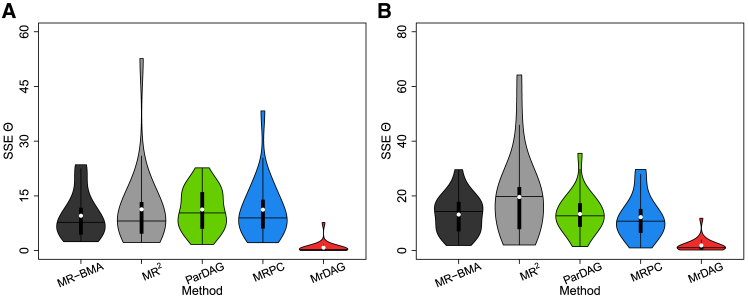
Violin plots of the sum of squares error (SSE) of the causal effects Θ between the exposures and the outcomes for all methods considered in the simulated scenarios ${\text{UndG}}_{X}$-${\text{DAG}}_{Y}$ and ${\text{DAG}}_{X}$-${\text{DAG}}_{Y}$ across 25 replicates in each scenario (A) In scenario ${\text{UndG}}_{X}$-${\text{DAG}}_{Y}$, the strength of correlation between consecutive X is set at $r_{X}=0.6$ and then decreases exponentially for non-consecutive exposures, and the average level of the mediation parameters within Y is set at $m_{Y}=1$. (B) In scenario ${\text{DAG}}_{X}$-${\text{DAG}}_{Y}$, the average level of the mediation parameters within X and Y is set at $r_{X}=0.6$ and $m_{Y}=1$, respectively. For details, see Appendix A. In each violin plot, the vertical black thick line displays the interquartile range, the black horizontal line denotes the median, and the white dot denotes the mean. For MRPC and ParDAG algorithms, we only show the results obtained at type I error rate for the conditional independence test α=0.01 and Lasso penalization λ=0.9, respectively. These values provide the best results for the two algorithms as shown in Figures 5 and S2.

Figure 6A shows the SSE of the causal effects Θ between the exposures and the outcomes for all methods considered in the simulated scenario ${\text{UndG}}_{X}$-${\text{DAG}}_{Y}$ and in Figure 6B for the simulated scenario ${\text{DAG}}_{X}$-${\text{DAG}}_{Y}$ across 25 replicates in each scenario with the same parameter settings and implementation of algorithms described above. For MRPC and ParDAG algorithms, we only show the results obtained at type I error rate for the conditional independence test α=0.01 and Lasso penalization λ=0.9, respectively. These values provide the best results for the two algorithms as shown in Figures 5 and S2.

MrDAG has the lowest SSE mean and median (white dots and horizontal black line, respectively) in both scenarios. As expected, when a topological ordered DAG is simulated within the exposures (Figure 6B), the violin plots have a wider range, showing more variable results, although the median is almost similar to the scenario with simulated partial correlation within X (Figure 6A). Alternative methods have larger SSEs.

Similar comments can be made for simulated scenarios ${\text{UndG}}_{X}$-${\text{Med}}_{Y}$ (Figure S3A) and ${\text{DAG}}_{X}$-${\text{Med}}_{Y}$ (Figure S3B), where a complete mediation is considered within the outcomes. MrDAG is confirmed as the best method.

We conclude this section by inspecting the sensitivity of the SSE of the causal effects between the exposures and the outcomes for different values of the average level of the mediation parameters $r_{X}$ and $m_{Y}$. The estimation of the causal effects displayed in Figure S7 shows that both MR-BMA and MRPC depend on the combination of $r_{X}$ and $m_{Y}$, with almost similar performance when a complete mediation is simulated (Figures S7A and S7C). MR^2^ has good performances across all scenarios compared to the other methods, but its behavior depends largely on the simulated level of $m_{Y}$, which, in turn, affects the estimated dependency relations within Y (see Figure S6). Compared to the other methods, MrDAG is not only the best, but it is rather insensitive to different levels of the mediation parameters within X and Y.

In summary, MrDAG has the lowest bias in the estimation of the causal effects in all simulation scenarios and for any combinations of the parameters $r_{X}$ and $m_{Y}$ that control the strengths of the pleiotropy within the exposure and the outcomes. The advantage of MrDAG is more pronounced when there are complex relationships within the responses or the outcomes, and, in particular, when both cases are simulated, which reflects more closely what happens in real-life applications.

### Robustness to noisy genetic association estimates and mis-specification of the exposure-outcome groups’ definition

We evaluate the robustness of the proposed MrDAG model by looking at the effect of noisy genetic association estimates and the mis-specification of the exposure-outcome groups’ definition.

Regarding the effect of imprecise genetic association estimates, we replicate the setup used in the simulated scenarios described above, but we decrease the number of individuals from N=100,000 to N=20,000, equally split between the exposures and the outcomes, i.e., $N_{Y}=N_{X}=10,000$. Although this can be considered an extreme case because the sample size in modern GWASs is much larger (see Table S1 for the number of individuals considered in the real data application), it reflects the presence of noisy genetic association estimates.

The results concerning the detection of the simulated sparse signals and the quality of the causal effect estimates are presented in Figures S8–S11 for all simulated scenarios and a particular choice of the parameters $r_{X}=0.6$ and $m_{Y}=1$. While there is a less clear advantage of the proposed MrDAG model over MR-BMA and ${\text{MR}}^{2}$ in all scenarios considered and a similar behavior regarding the detection of the simulated causal effects with the MRPC algorithm (see Figures S8 and S9), MrDAG outperforms the other methods in the quality of the causal effect estimates when dependency relations are simulated within the outcomes (see Figure S11). Only in one case do we record a worse performance of the proposed model than alternative methods, specifically against MR-BMA and ${\text{MR}}^{2}$ in their most favourable simulated scenario ${\text{UndG}}_{X}$-${\text{Med}}_{Y}$ (see Figure S10A).

Regarding the robustness to the mis-specification of the exposure-outcome groups’ definition, we took the datasets originally simulated and incorrectly defined the two groups. Specifically, for all algorithms considered, 5 exposures are now mis-specified as outcomes, which reduced the group of exposures from 15 to 10 and increased the group of outcomes from 5 to 10. In doing so, reverse causation, originally not considered in the simulation study, is now present. For instance, this happens if, in a relation dependence between two exposures, the parent node is mis-specified and wrongly assigned to the outcomes group.

We evaluate the ability of the algorithms to detect the simulated sparse signals based not only on a subset of the causal effects Θ originally simulated from the exposures to the outcomes (i.e., selecting 10×5 exposure-outcome combinations from the original simulated 15×5) but also on a subset of the sparse signals $\Gamma_{X}$ that originally was simulated within the exposures (i.e., including 10×5 exposure-exposure combinations from the original simulated 15×15). Overall, in all mis-specified scenarios, the number of exposure-outcome combinations where the sparse causal effects might be present increases from 15×5=75 to 10×10=100.

The results of this experiment regarding the detection of the simulated sparse signals and the quality of the causal effects estimation for all simulated scenarios, and a particular choice of the parameters $r_{X}=0.6$ and $m_{Y}=1$, are presented in Figures S12–S15. As expected, the detection of the simulated causal effects is more difficult, although MR-BMA depends less on the mis-specification given that it does not model dependency relations within either the exposures or the outcomes. MrDAG is less influenced than the other causal graphical algorithms and particularly MRPC, and it still has a clear edge on MR-BMA when dependency relations are simulated within the outcomes (see Figures S13A–S13D). Regarding the quality of the causal effects estimation, MrDAG is confirmed to be the overall best method (see Figures S14 and S15). MR-BMA is the second-best method when no dependency structure is simulated within the outcomes.

### Real data application: The impact of lifestyle and behavioral traits on mental health

We apply MrDAG to investigate its ability to detect the effects of lifestyle and behavioral exposures on the risk of mental health phenotypes as well as potential forms of interventions for their prevention. As exposures, we chose seven lifestyle and behavioral traits that have previously been investigated for their effects on mental health, including EDU, physical activity (PA), sleep duration (SP), alcohol consumption (ALC), SM, and LST. As outcomes, we selected seven mental health phenotypes, including MDD, AN, attention-deficit hyperactivity disorder (ADHD), BD, autism spectrum disorder (ASD), schizophrenia (SCZ), and COG. See Table S1 for the description of the summary-level statistics, the data sources, and the number of IVs for each trait, and the Appendix A for the pre-processing steps. In a separate analysis, we also investigate the reverse direction, i.e., whether the same mental health phenotypes have an impact on the group of lifestyle and behavioral traits selecting IVs for the mental health phenotypes (see Appendix A for the respective pre-processing steps).

**Figure 7:**
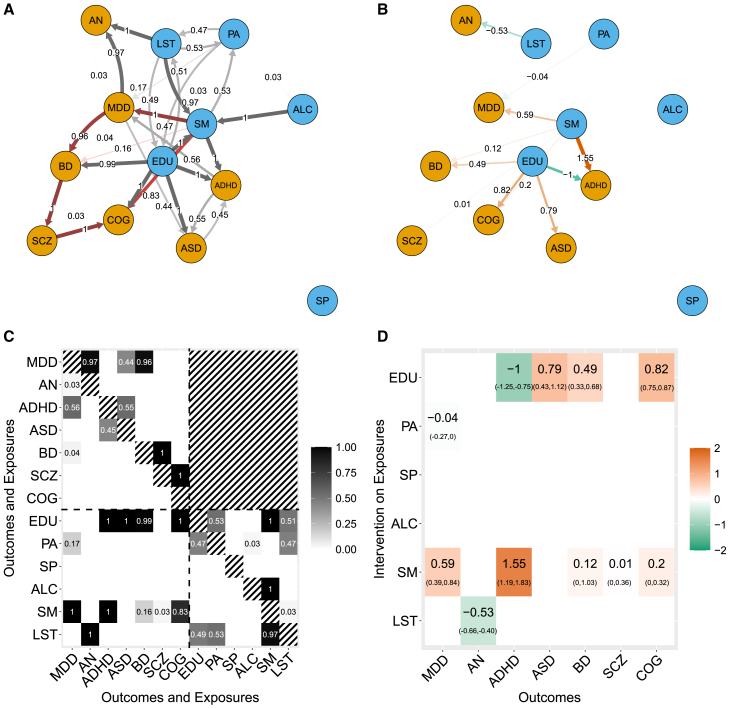
Results of MrDAG algorithm regarding how lifestyle and behavioral exposures impact mental health outcomes (A) PDAG of the posterior probability of edge inclusion (PPEI) within the exposures (lifestyle and behavioral traits, blue nodes), the outcomes (mental health phenotypes, orange nodes), and between them. Undirected edges are represented as bidirectional edges; see, for instance, edges between PA (physical activity) and LST (leisure screen time) or ASD (autism spectrum disorder) and ADHD (attention-deficit hyperactivity disorder). Red edges indicate the estimated direct and indirect path from SM (smoking) to COG (cognition), including the path from SM to SCZ (schizophrenia). Neither reverse causation from the outcomes to the exposures nor phenotypic traits feedback loops are allowed. (B) (Bayesian model-averaged) Causal effects on the outcomes (orange nodes) under intervention on the exposures (blue nodes). Red and green edges indicate positive and negative (Bayesian model-averaged) causal effects, respectively. (C) Posterior probability of edge inclusion (PPEI) for each combination of outcomes (mental health phenotypes) and exposures (lifestyle and behavioral traits). Horizontal and vertical dotted lines separate the exposures (bottom-right submatrix) from the outcomes (top-left submatrix). PPEIs between exposures and outcomes are depicted in the bottom-left submatrix. Neither reverse causation (top-right submatrix) nor phenotypic traits feedback loops (main diagonal) are allowed (black-white strips). (D) (Bayesian model-averaged) Causal effects (95% credible intervals) on the outcomes (y axis) under intervention on the exposures (x axis).

Figure 7 presents the results of MrDAG. In particular, Figures 7A and 7C show the estimated PPEI (Equation A14) after structure learning, and Figures 7B and 7D show the (Bayesian-model-averaged) causal effects (95% credible intervals [CI]) between the exposures and the outcomes. We ran the MrDAG algorithm for $10^{6}$ Markov chain Monte Carlo (MCMC) sweeps, $10^{5}$ of which as burn-in (for details see supplemental text). The computational time is 1 h 40 min on Cambridge HPC. Figure S16A shows no sign of aberrant behavior of the MCMC or the Markov chain being trapped in local maxima. The number of sweeps seems sufficient for the convergence of the MCMC algorithm to draw samples from the posterior distribution. Results on PPEI and the causal effects are not thresholded, and sparsity is enforced by assigning *a priori* on the number of expected edges. We set it at $\pi^{\text{edge}}=0.16$, i.e., we expect *a priori* one edge for each of the 13 traits (see Appendix A and supplemental text). Post-processing of the MrDAG output and corresponding outliers detection (CPO estimation) show no invalid IVs due to unmeasured pleiotropy that acts on a single outcome at a time (see Appendix A and Figure S17). The time required to estimate CPO is 3 h 30 min, higher than the MrDAG algorithm, since the marginal likelihood has to be calculated for each observation across all the graphical models visited during the MCMC. However, it is less than the computation time of the MrDAG algorithm × the number of observations, since no structure learning needs to be performed and, thus, there is no need to evaluate the time-consuming Metropolis-Hastings ratio in Equation A11.

As shown in Figures 7C and 7D, there are two key shared exposures with important downstream effects on mental health phenotypes, which are EDU and SM, on which we focus our discussion. For each of them, we describe how MrDAG can disentangle complex dependency relations within the exposures and the outcomes and detect (partial or complete) mediation, which prevents spurious findings.

As could be expected due to its centrality in the global health agenda^54^ and the high level of confounding of this phenotype with other genetically associated biological, behavioral, and socioeconomic traits, genetically predicted EDU shows the most inter-exposure and exposure-outcome dependency relations (Figure 7C, bottom part). Previous work has supported the broad mental health implications of education.^55^ First, in keeping with previous findings,^26^^,^^56^^,^^57^^,^^58^ our results show that genetically predicted EDU increases COG, and liability to ASD and BD, as well as decreasing liability to ADHD. In contrast, genetically predicted EDU has no effects on SP, the amount of ALC, or the liability to MDD,^26^ AN,^59^ or SCZ^58^ (Figure 7D). Second, we investigate the detected dependency relations of EDU with other exposures that contribute to the reported associations. We find bidirectional relationships between genetically predicted EDU, PA, and LST consistent with a large body of literature.^26^^,^^60^ Dependency relations have been also identified between EDU and SM.^26^^,^^27^ Supported by the existing literature, these results confirm the ability of MrDAG to disentangle complex relationships that exist between inter-related exposures.

We find that SM is second only to EDU in its association with several outcomes. Specifically, genetically predicted levels of SM are associated with an increased liability to MDD and ADHD, as previously reported.^61^^,^^62^ It is also associated with BD and SCZ (although these effects are small) and COG. Notably, the association between genetically predicted levels of SM and COG is also detected by standard MR but with a negative effect (see Figure 1A), while MR-BMA^1^ does not declare it significant after FDR control across all exposures and outcomes, although it estimates a positive effect (see Table S3). As discussed above, we also check the detected dependency relations of SM with other exposures. MrDAG appropriately identifies the relationship between ALC and SM, but not vice versa. In a recent MR publication,^63^ the opposite association is observed. However, in contrast to Reed et al.,^63^ who conceptualize SM with smoking initiation, we use a lifetime smoking index,^61^ which captures smoking duration, heaviness, and cessation.

As important as the discussion of existing associations between the exposures and the outcomes is, it is similarly insightful to discuss the absence of causal effects, especially those relationships that are reported in the literature or found by standard (one exposure and one outcome) MR models. For example, we do not replicate all previous evidence for genetically predicted levels of SM being associated with mental health phenotypes. Although we find a strong effect of genetically predicted levels of SM on MDD,^61^ we do not find the same strong effect of SM on SCZ^61^ as observed in observational studies.^64^^,^^65^ By looking at Figure 7C, this might be due to pleiotropic effects that have been identified by MrDAG within the mental health phenotypes. In line with prior findings, evidence from MrDAG supports dependency relations between genetic liability to MDD and AN, ASD, and BD^28^ as well as between genetic liability to BD and SCZ.^66^ Lastly, in keeping with prior findings of possible bidirectional ASD-ADHD relationships,^67^ we observed genetic dependency relations between ASD and ADHD, and vice versa. These results suggest that the genetic effects of SM on SCZ can be mediated by pleiotropic effects within the responses. By considering the results above, we hypothesize that the SM-SCZ relationship is partly mediated first by MDD and then by BD. Moreover, there is another path that goes from the genetically predicted level of SM to SCZ through a positive weak association identified by MrDAG between SM and BD.^68^ Both genetic paths are illustrated in Figure 7A and highlighted in red. Conditionally on these relationships that are not considered in standard MR or MVMR, MrDAG does not detect a strong causal effect between SM and SCZ.

We further note that the effect of SM on ADHD is both direct and indirect, the latter mediated first by MDD and then by ASD. Thus, our analysis pinpoints the important role of MDD, which partly or entirely accounts for many paths within mental health phenotypes and their causal exposures. This might be due to the potentially high levels of confounding and non-specific genetic associations present in the original MDD GWAS^69^^,^^70^ as well as the high levels of symptom-level and, therefore, diagnostic overlap between MDD and all other psychiatric disorders.^71^ Nonetheless, the implications of our results, assuming the validity of all GWAS findings, are that prevention and/or therapeutic intervention on MDD^72^ can have a cascade of important effects for the prevention of several mental health phenotypes.

To investigate this hypothesis, Figures S18A and S18B show the results of MrDAG when MDD is removed from the list of outcomes. Regarding the association between genetically predicted levels of SM and ADHD, it is still present with the same strength and similar CI depicted in Figure 7D, suggesting that the indirect effect mediated first by MDD and then by ASD is negligible. Figure S18B also shows that, after removing MDD, genetically predicted levels of SM are positively associated with SCZ, as reported in the literature, as well as negatively associated with LST, as shown in Figure 1. Combined with our main findings, this result indicates that the absence of a link between SM and SCZ (and the link between LST and SCZ) in the MrDAG model is likely due to the mediation of MDD. Similar results are obtained if BD, which appears in the path highlighted in red in Figure 7A, is removed from the list of outcomes (see Figure S19).

**Figure 8:**
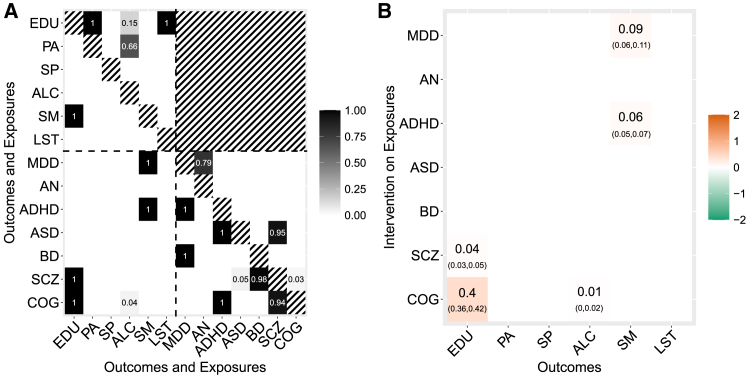
Results of MrDAG regarding how liability to mental health phenotypes affects lifestyle and behavioral traits after removing invalid IVs (A) Posterior probability of edge inclusion (PPEI) for each combination of outcomes (lifestyle and behavioral traits) and exposures (mental health phenotypes). Horizontal and vertical dotted lines separate the exposures (bottom-right submatrix) from the outcomes (top-left submatrix). PPEIs between exposures and outcomes are depicted in the bottom-left submatrix. Neither reverse causation (top-right submatrix) nor phenotypic traits feedback loops (main diagonal) are allowed (black-white strips). (B) (Bayesian model-averaged) Causal effects (95% credible intervals) on the outcomes (y axis) under intervention on the exposures (x axis).

To check whether the hypothesized edges’ orientation between the exposures and the responses might not be supported by the data, we have also tested reverse causation, whereby we assess the impact of mental health phenotypes on lifestyle and behavioral traits by selecting genetic variants to be associated with the mental health phenotypes. As before, we used $10^{6}$ MCMC sweeps of which $10^{5}$ were burn-in, and the computational time is 2 h on the Cambridge HPC. Post-processing of MrDAG output to check for the presence of invalid IVs due to unmeasured pleiotropy that acts on a single outcome at a time identifies 84 IVs (18%) as invalid (see Figure S26). CPO estimation took 3 h to run. We removed these IVs and rerun MrDAG on the remaining 386 IVs. Figures 8 and S27 show the results of this analysis where, besides the positive effect of genetically predicted COG on EDU,^73^ genetic liability to MDD^61^ and ADHD is associated with SM, the latter well documented in epidemiological studies^74^ and confirmed in a randomized clinical trial of smoking cessation,^75^ although the respective effect size is small.

We conclude the analysis of the real data application by assessing the validity of the results obtained by MrDAG and adding the comparison with other methods. We divide this internal check into sensitivity to hyper-prior specification and robustness of structure learning. Regarding the first point, Figure S20 shows that the (Bayesian model-averaged) causal effects as well as the 95% CIs for different values of the *a priori* probability of edge inclusion are not influenced by this choice. For the second internal check, we bootstrap MrDAG repeatedly on the data^76^ (see supplemental text). In Figure S21 we present the bootstrap frequency of edge inclusion for each permitted combination of exposures and outcomes and the scatterplot of the PPEI against the bootstrap frequency of edge inclusion. The results show that there is a satisfactory agreement between a single run of the algorithm and the bootstrap results for the reported causal associations. Extended results are presented in supplemental text.

Figures S22–S25 show results of ${\text{MR}}^{2}$, MRPC, ParDAG, and Graph-MRcML applied to the same real data. We discuss in detail the results of ${\text{MR}}^{2}$ and Graph-MRcML.

As in the simulation study, ${\text{MR}}^{2}$ shows some difficulties in teasing out complex relationships within the responses, which are mostly directed, e.g., MDD → BD, or mediated, e.g., ADH → MDD → AN, and detected by ${\text{MR}}^{2}$ as MDD-BD and ADHD-AN, once the causal effects have been detected, and vice versa.

Graph-MRcML is the only method considered that treats all traits as equal and does not distinguish between exposures and outcomes. To perform this task, it requires a different format for the data input (see supplemental text). The results show generally a good agreement with MrDAG regarding EDU and SM as effective points of intervention, and the absence of any direct effect of SM on SCZ. They also largely agree on the lack of reverse causation, with the effect of COG on EDU detected by both. However, two important differences are apparent. They are related to MrDAG shrinkage of unimportant causal effects and sparse structure learning. In Graph-MRcML, the total effects are decomposed into the direct effects of all possible trait pairs (see Figure S25B), including the non-significant ones (see Figure S25C), leading to the “dilution” of the causal effect size of significant trait pairs. An example of this phenomenon is the effect of SM on SCZ. Albeit not selected at 5% Bonferroni-adjusted significance level $\left(p_{\text{adj}}=1\right)$, the direct causal effect of SM on SCZ is one of the largest estimated by Graph-MRcML, weakening the estimated causal effects of the other trait pairs; for example, the size of the causal effect of SM on ADHD obtained by Graph-MRcML (1.03) is 50% lower compared to MrDAG (1.55). In contrast, MrDAG estimates the direct causal effects given the sparse visited EGs, on average with ≈21 edges out of 114 possible ones (Figure S16), thus shrinking to zero the causal effects of unimportant dependency relations. The second difference is linked to the selection of important relationships. In MrDAG this is accomplished by a score-based (marginal likelihood) structure learning of sparse EGs visited during the MCMC, which separates important dependency relations from less important ones. In Figure S20 we show that the results do not depend on the *a priori* probability of edge inclusion. Contrarily, in Graph-MRcML small differences in the adjusted significance level lead to different models. An example is the effect of SM on ADHD (Figure S25A), which is not significant at 5% Bonferroni-adjusted significance level $\left(p_{\text{adj}}=0.051\right)$, although this relation has been detected by MrDAG and the majority of alternative approaches. At higher Bonferroni-adjusted significance level it becomes significant. Another example is the effect of EDU on ASD, which has an adjusted p value that is only slightly higher than 20% $\left(p_{\text{adj}}=0.208\right)$. It is not clear whether or not it should be included in the causal graphical model.

## Discussion

In this study, we have introduced MrDAG, the first Bayesian causal graphical MR model for joint analysis of multiple exposures and outcomes. The proposed method can detect dependency patterns within the exposures as well as within the outcomes, thus allowing for a more precise estimation of the causal effects from the exposures to the outcomes.

In a comprehensive simulation study, MrDAG outperforms recently proposed one-outcome-at-a-time and multi-response multi-variable MR methods and causal graphical models under the constraint on edges’ orientation from the exposures to the outcomes. We showcased the advantage of MrDAG also in a real data application to disentangle how lifestyle and behavioral traits interact to cause mental health phenotypes and, separately, the opposite. We highlighted how MrDAG can recover information on the genetic paths that link exposures to outcomes compared to existing MR methods that ignore these dependency relations. Specifically, we highlighted primarily, education and secondarily, smoking as solely effective points of intervention given their distinct downstream effects on multiple mental health phenotypes. In contrast to widely used uni- and multi-variable MR methods, and a recently proposed multi-response model, neither leisure screen time (LST) nor sleep duration (SP) have been identified as key exposures of intervention. Compared to other causal graphical models considered in this study under the assumption of known directionality between the exposures and the outcomes, more significant causal effects are detected by MrDAG, especially those linked with education. Finally, by enforcing sparsity, MrDAG better separates important dependency relations from less important ones and better estimates direct causal effects than an alternative bidirectional causal graphical model where no regularization of the estimated causal effects is implemented.

These insights are possible because three methodological advances are considered in MrDAG. First, in structure learning, the hypothesis of no unobserved confounding is a fundamental underlying assumption. This assumption, known as causal sufficiency, is difficult to justify in real data applications, and its violation produces biased results. By using IVs within the MR paradigm, we bypass the need to remove the effects of the unobserved confounder from the individual-level data.^39^^,^^77^^,^^78^ Specifically, we avoid the assumption of causal sufficiency by employing genetically predicted exposures and outcomes that depend only on the genetic variants chosen as IVs. Genetically predicted exposures are key in the derivation of the two-stage least-square causal effect estimator,^41^ but in MrDAG we have extended it to include genetically predicted outcomes. On both predicted groups of traits, we perform EG exploration to learn the unconfounded dependency relations that exist within and between the exposures and the outcomes. Our second contribution is the estimation of causal effects under intervention on the exposures conditionally on a given DAG. We showed that they can be estimated based on Pearl’s interventional calculus.^17^ Moreover, differently from Kalisch et al.^50^ and its application in the MRPC algorithm,^5^^,^^51^ in the proposed Bayesian implementation the estimation of the causal effects is averaged over the visited graphical models,^43^ thus taking into account the uncertainty regarding the EGs that best describe the dependency structure in a given dataset. Third, MrDAG allows the possibility of including domain-knowledge relations between the traits. In the designed MrDAG model, constraints between the exposures and the outcomes descend directly from the MR paradigm. Our Bayesian implementation of structure learning under restrictions offers clear advantages over alternative methods.^44^ In particular, adding an acceptance/rejection step to check whether the proposed EG satisfies the edge-orientation constraints is simple and effective. Although not presented in this study, other restrictions can be straightforwardly included—for instance, known relations regarding disease progression or time-dependent outcomes, e.g., smoking initiation and cessation.^79^

MrDAG is an MR approach that is best suited in the context of biologically informed relationships, since exposures and outcomes need to be specified before the analysis. This insight should be used to inform the design of the study as well as the data sources selected to test specific research questions and hypotheses. The lack of sufficient appreciation of underlying biology is one of the reasons for the current crisis faced in the application of MR more widely.^80^ In the case of no prior domain knowledge, agnostic causal network models should be preferred.^6^

There are some limitations in the proposed method. A drop in power to detect causal effects is apparent in the simulation study when the summary-level data are derived from noisy genetic association estimates. This is expected, since the detection of dependency relations requires informative data. As we have shown in the real data application, modern GWASs are performed on large cohorts, so noisy genetic association estimates are less of a concern, although this might still be an issue (shared with most causal graphical models) when molecular traits are considered, since their sample size could be small.^81^ Mis-specification of the exposure-outcome groups’ definition has the same effect on the power, since the hypothesized edges’ orientation between the exposures and the responses might not be supported by the data, e.g., if reverse causation is present. This can be investigated by flipping the comparison and treating the outcomes as exposures and vice versa. This entails selecting a new set of genetic variants as IVs to instrument the outcome traits and rerunning MrDAG to check for reverse causation, as illustrated in the real data application where we investigated the reverse direction, i.e., the impact of liability to mental health phenotypes on lifestyle and behavioral traits. We also suggest monitoring the rate of the rejection step. High levels might be an indicator of reverse causation and, thus, mis-specification of the exposure/outcome groups’ definition.

In the real data application, while the use of existing summary-level statistics of GWASs facilitates the integration of diverse phenotypes measured in different cohorts, we are also limited by the biases suffered by the initial GWASs. Specifically, studies of mental health rely on the presence of a clinical diagnosis. Consequently, it is not truly the genetic liability of the disease itself as much as the probability of having access to diagnoses or treatment. Our findings on the relationship between higher genetically predicted EDU and increased risk of ASD and BD, but decreased ADHD risk, provide an example of such bias. In these analyses, the predicted number of school years completed is unlikely to be causally implicated in the development of ASD traits. While the typical age of onset of ASD precedes the start of formal education (and is therefore unlikely to be caused by it), ASD-related traits are more likely to be recognized and referred, particularly in those who are undiagnosed or untreated, when individuals are within a schooling system where standardized testing and progress reports by peer comparison are performed. Moreover, current GWASs consider one trait or disease at a time and do not consider to what extent cases are comorbid with other diseases. Future GWASs on comorbidity^82^ might provide more fine-grained genetic associations, allowing disentanglement of some of these relationships.

In conclusion, MrDAG, with its unidirectional approach and Bayesian implementation, represents an alternative contribution to how we can learn complex relationships among phenotypic traits. It provides analysts with the opportunity to derive a more comprehensive picture of causal mechanisms between complex phenotypes. The real data application is an example of the proposed holistic approach, whereby we leverage MrDAG and large-scale genome-wide association data to offer novel mechanistic insight into the causal behavioral determinants of mental health phenotypes to delineate between their overlapping pathophysiology and phenotypic presentation toward translational progress in the field of mental health.

## Data and code availability

Data sources are presented in supplemental text with associated URL links. Social Science Genetic Association Consortium (SSGAC) summary-level statistics are available through a standard registration procedure (https://thessgac.com/register/).

The MrDAG learning R package is freely available on https://github.com/lb664/MrDAG/. It includes the data of the real data applications and how to run the algorithm. Post-processing routines to estimate the (Bayesian model-averaged) causal effects presented in this article are also included along with PPEI.

## Acknowledgments

The authors are thankful to Federico Castelletti and Guido Consonni for their insightful comments and suggestions and to the participants of the 1st Danish International Conference on Personalized Medicine, Aarhus, Denmark, whose remarks regarding preliminary results of the real data application led to its substantial improvement. The authors are also grateful to the editor and two anonymous referees for their valuable comments that greatly improved the presentation of the article and for pointing out useful references on causal graphical models.

The authors gratefully acknowledge United Kingdom Research and Innovation Medical Research Council grants MR/W029790/1 (V.Z. and L.B.), the Marmaduke Sheild Fund (L.B.), and the British Heart Foundation Centre of Research Excellence at Imperial College London grant RE/18/4/34215 (D.G.). This research was supported by the 10.13039/501100017510UK Dementia Research Institute (V.Z.), which receives its funding from 10.13039/501100017510UK DRI, funded by the UK MRC, Alzheimer’s Society, and Alzheimer’s Research UK, and by the 10.13039/501100018956NIHR Cambridge Biomedical Research Centre (NIHR203312) (L.B.). The views expressed are those of the authors and not necessarily those of the 10.13039/501100000272NIHR or the 10.13039/501100000276Department of Health and Social Care.

## Author contributions

Conceptualization, L.B., V.Z., and D.G.; methodology: L.B. and V.Z.; formal analysis, V.Z. and L.B.; resources, all collaborators; data curation, V.Z. and D.G.; writing – original draft, V.Z., L.B., T.C., D.G., and N.C.; writing – review & editing, L.B., V.Z., and D.G.; visualization, V.Z. and L.B.; funding acquisition, V.Z.; supervision, L.B. and D.G.

## Declaration of interests

D.G. is the Chief Executive Officer of Sequoia Genetics. D.G. has financial interests in several biotechnology companies. T.C. is employed by Sequoia Genetics.

## Appendix A

In the following, we denote with capital letters the random variables Y,X,G, and U for the observed outcome, exposure, instrumental variable, and unobserved confounder, respectively, and with small letters y,x,g, and u their corresponding observations. Multivariate random variables and corresponding observations are presented in bold. A marginal element of a vector of random variables is specified by a suitable subscript index, e.g., $Y_{k},k\in K=\left\{1,\ldots ,q\right\},X_{j},j\in J=\left\{1,\ldots ,p\right\}$, and $G_{i},i\in I=\left\{1,\ldots ,n\right\}$. $Y_{\setminus k}$ and $X_{\setminus j}$ consist of all the outcomes and exposures except those that are related to the kth response and jth exposure, respectively. Finally, vectors understood as column vectors and matrices are indicated in bold, the latter also in capital letters.

We indicate with $\beta_{i,j}^{X}$ and $\beta_{i,k}^{Y}$ the effect of the genetic variant i∈I on the exposure j∈J and outcome k∈K, respectively, with $\beta_{X_{j}}$ and $\beta_{Y_{j}}$ the n-dimensional vector of genetic effects on the jth exposure and kth outcome, respectively, and, finally, with $B_{X}$ and $B_{Y}$ the (n×p)- and (n×q)-dimensional matrices of the genetic effects on all exposures and outcomes. $\theta_{j,k}$ denotes the causal parameter of interest, i.e., the direct causal effect of $X_{j}$ on $Y_{k}$, and $\gamma_{h,j}^{X}$ and $\gamma_{h,k}^{Y}$ the mediation effect of $X_{h}$ on $X_{j},h\neq j$ and $Y_{j}$ on $Y_{k},h\neq k$, respectively. $\Theta ,\Gamma_{X}$, and $\Gamma_{Y}$ indicate the corresponding (p×q)-, (p×p)-, and (q×q)-dimensional matrices of the causal parameters of interest (Θ) and the mediation parameters $\Gamma_{X}$ and $\Gamma_{Y}$ whose average value is $r_{X}$ and $m_{Y}$, respectively. The symbol “∧” denotes the estimator of a parameter or its estimated value and “^∗^” an IVW parameter.

Let D=(V,E) be a directed acyclic graph (DAG), where V denotes a set of vertices (nodes) and E=V×V a set of directed edges, i.e., if (z,v)∈E, then (z,v)∉E. For a given DAG D, if z→v, then z is a parent of v and, conversely, v is a child of z. Moreover, if z→…→v, then z is an ancestor of v and v is a descendant of z. We denote the parent set of v in D as ${\text{pa}}_{D}\left(v\right)$ and $v\cup {\text{pa}}_{D}\left(v\right)={\text{fa}}_{D}\left(v\right)$ the family of v. Unless otherwise stated, for ease of notation, we remove the subscript D. We indicate with $Y_{\text{pa}\left(k\right)}$ and $X_{\text{pa}\left(k\right)}$ the outcome and exposure parents of the outcome k∈K, with $X_{\text{pa}\left(j\right)}$ the exposure parents of the exposure j∈J and with $X_{\text{fa}\left(j\right)}$ its family, i.e., $X_{\text{fa}\left(j\right)}=X_{j}\cup X_{\text{pa}\left(j\right)}$.

In research by Didelez and co-workers,^15^^,^^37^^,^^38^ key results regarding standard Mendelian randomization (MR) (single exposure with single instrumental variable and single outcome) are presented. Here, we use them to show that MrDAG is an extension of standard MR when multiple exposures and outcomes are considered. Technical details are provided in supplemental text.

These results are conditioned on a given DAG. Since the underlying dependency relations within and between exposures and outcomes are not known (latent) and need to be estimated, structure learning is performed on the genetically predicted values of the traits. We draw the connection between them and the genetic components of the phenotypic covariance, which are orthogonal to any confounders U. Thus, by using the genetically predicted values, the causal sufficiency condition (usually assumed explicitly or implicitly in structure learning) is not required. We conclude this section with a description of the Bayesian implementation of the proposed model and details regarding the data-generation steps used in the simulation study.

### Multi-exposure and multi-outcome core conditions for instrumental variables

Let Y,X, and G be the q-, p- and n-dimensional vector of the outcomes, exposures and instruments (genetic variants) random variables, respectively.

Let us assume the following “multi-variate core conditions” (MCC) for valid instrumental variables (IVs), which are the extensions of the core conditions that G has to satisfy in standard MR^15^:

(IV1) $G_{i}⫫U,\forall i\in I$, i.e., $G_{i}$ must be independent of U;

(IV2) , i.e., $G_{i}$ must not be independent of $X_{j}$ conditionally on $X_{\setminus j}$; and

(IV3) $G_{i}⫫Y_{k}|\left(X,U\right),\forall i\in I,\text{and}\;\forall k\in K$, i.e., $G_{i}$ must be independent of $Y_{k}$ conditionally on X and U.

The first multi-exposure and multi-outcome core condition (MCC) for instrumental variables is similar to the first CC in standard MR.^15^ The second MCC imposes that $G_{i}$ should be associated with $X_{j}$ conditionally on the other exposures. The third MCC establishes that the instrumental variables and outcomes are conditionally independent given the exposures and the unobserved confounder.

From the DAG D involving Y,X,G, and U that satisfies the MCC, the corresponding Markov properties say that $G_{i}⫫U,\forall i\in I$, since $G_{i}$ is not a descendant of U and vice versa and , because $X_{j}$ is a descendant of $G_{i}$. The Markov property for the third MCC is $G_{i}⫫Y_{k}|\left(Y_{\text{pa}\left(k\right)},X_{\text{pa}\left(k\right)},U\right),\forall i\in I\;\text{and}\;\forall k\in K$, since $G_{i}$ is a non-descendant of $Y_{k}$ and $\left(Y_{\text{pa}\left(k\right)},X_{\text{pa}\left(k\right)},U\right)$ are the parents of $Y_{k}$.

### Interventional distributions and causal effects estimation

The conditional dependencies associated with the multi-exposure and multi-outcome DAG D lead to the following factorization of the joint density of all random variables considered:

$$f\left(y,x,g,u\right)=\prod_{k\in K}f\left(y_{k}|y_{\text{pa}\left(k\right)},x_{\text{pa}\left(k\right)},u\right)\prod_{j\in J}f\left(x_{j}|x_{\text{pa}\left(j\right)},g,u\right)f\left(g\right)f\left(u\right)\text{,}$$

which is known as pre-intervention distribution and assumed to be faithful to the DAG,^53^ i.e., there are no conditional dependence relationships between the variables in the model that do not follow directly from the Markov properties.

The post-intervention distribution under intervention on the hth exposure sets to take the value ${\tilde{x}}_{h}$ is obtained by the truncated factorization^17^

(Equation A2) $$\begin{array}{cc} f\left(y,x_{\setminus h},g,u|\text{do}\left(X_{h}={\tilde{x}}_{h}\right)\right) & =\prod_{k\in K}f\left(y_{k}|{\tilde{x}}_{h},y_{\text{pa}\left(k\right)},x_{\text{pa}\left(k\right)},u\right)\\ & \;\prod_{j\in J\setminus \left\{h\right\}}f\left(x_{j}|x_{\text{pa}\left(j\right)},g,u\right)I_{x_{h}}\left({\tilde{x}}_{h}\right)f\left(g\right)f\left(u\right) \end{array}\text{,}$$

where $I_{x}\left(\tilde{x}\right)$ is the indicator function which is equal to one if $x_{h}={\tilde{x}}_{h}$ and zero otherwise. Graphically, the directed edges to $X_{h}$ from its parents in X,G and U are removed.

A post-intervention distribution under intervention on the hth exposure is obtained from Equation A2 by marginalizing all variables but the selected outcome and the exposure on which an intervention is carried out:

(Equation A3) $$\begin{array}{cc} f\left(y_{k}|\text{do}\left(X_{h}={\tilde{x}}_{h}\right)\right) & =\int f\left(y,x_{\setminus h},g,u|\text{do}\left(X_{h}={\tilde{x}}_{h}\right)\right)\;dy_{\setminus k}dx_{\setminus h}dg\;du\\ & =\int f\left(y_{k}|{\tilde{x}}_{h},x_{\text{pa}\left(h\right)},u\right)f\left(x_{\text{pa}\left(h\right)},u\right)I_{x_{h}}\left({\tilde{x}}_{h}\right)\;dx_{\text{pa}\left(h\right)}du \end{array}$$

This result is derived from Pearl^17^ and follows directly from the Markov properties of the DAG. It establishes that the parents of the variable on which an intervention is carried out are the only variables that need to be measured to estimate the causal effect on an outcome.^83^

The post-intervention distribution (Equation A3) can be summarized by taking the expectation and defining the causal effect of an intervention^84^ as

$$\theta_{h,k}={\frac{\partial }{\partial x_{h}}E\left(Y_{k}|\text{do}\left(X_{h}=x_{h}\right)\right)|}_{x_{h}={\tilde{x}}_{h}},h\in J,k\in K\text{.}$$

In supplemental text, we show the identifiability of the causal effect (proposition S2) and the derivation of its estimand in multiple exposures and multiple outcomes MR framework (proposition S3). We also show the consistency of the effects of the regressions of each outcome and exposure on G (proposition S1), i.e., the estimated genetic effects on the outcomes and exposures contain all information regarding the causal parameters of interest and the mediation parameters within the exposures and the outcomes unconfounded by U.

Here, for a given DAG D, we report the IVW estimator of the causal effect of the intervention in $X_{h}$ on $Y_{k}$:

(Equation A4) $${\hat{\theta }}_{h,k}={\left[{\left({\hat{B}}_{X_{\text{fa}\left(h\right)}}^{*⊤}{\hat{B}}_{X_{\text{fa}\left(h\right)}}^{*}\right)}^{-1}{\hat{B}}_{X_{\text{fa}\left(h\right)}}^{*⊤}{\hat{\beta }}_{Y_{k}}^{*}\right]}_{1}\text{,}$$

where the subscript indicates the first element of the solution of the linear least squares (LLS) regression, since $\text{fa}\left(v\right)=v\cup \text{pa}\left(v\right),X_{\text{fa}\left(h\right)}$ denotes the exposures that are the family of the exposure $X_{h}$ under intervention, ${\hat{B}}_{X_{\text{fa}\left(h\right)}}^{*}$ are the IVW estimated coefficients of the regressions of each exposure in $X_{\text{fa}\left(h\right)}$ on G, and ${\hat{\beta }}_{Y_{k}}^{*}$ is the IVW estimated coefficient of a regression of $Y_{k}$ on G. Equation A4 resembles the standard IVW estimator of the causal effect that approximates the estimate that would have been obtained if individual-level data were available.^3^ However, in contrast to general proposed solutions in MVMR, in Equation A4 the set of regressors is with regard to the family of the exposure under intervention.

### Dependency structure under the effect of unobserved confounders

To estimate Equation A4, structure learning of the graphical models needs to be performed to detect the parents $X_{\text{pa}\left(h\right)}$ of the exposure $X_{h}$ under intervention. However, structure learning assumes causal sufficiency,^39^ i.e., it requires that there are no hidden (or latent) variables that are common causes of two or more traits. Instead, in this study we explicitly assume that an unobserved confounder U acts on both outcomes and exposures.

Links between the genetic correlation and MR causal effect estimate have been already discussed in Bulik-Sullivan et al.^85^ Here we provide further connections with genetic covariance,^86^ which is key to showing that, by working with summary-level statistics, it is possible to recover the dependency structure between the corresponding traits in the original (individual-level) data unconfounded by U.

Let us assume that the genetic effect on a phenotypic trait is linear and consider two traits,

$$\begin{array}{cc} Y_{k} & =G^{⊤}\beta_{Y_{k}}+\psi_{Y}U+\epsilon_{Y_{k}},k\in K\text{,}\\ X_{j} & =G^{⊤}\beta_{X_{j}}+\psi_{X}U+\epsilon_{X_{j}},j\in J\text{,} \end{array}$$

where G is a set of genetic variants of dimension n, either spanning the whole genome or region(s)-specific or selected to be associated with a trait, $\beta_{Y_{k}}$ and $\beta_{Y_{k}}$ are the genetic effects, U is an unobserved confounder that affects both traits with $\psi_{Y}$ and $\psi_{X}$ the effects sizes, and $\epsilon_{Y_{k}}$ and $\epsilon_{X_{k}}$ are white noises that can be interpreted as environmental effects. We assume that G⫫U and, similarly, $G⫫\epsilon_{Y_{k}}$ and $G⫫\epsilon_{X_{j}}$. Finally, we assume that $U⫫\epsilon_{Y_{k}}$ and $U⫫\epsilon_{X_{j}}$, i.e., the unobserved confounder U exerts its effect on both traits and it is distinct from other environmental factors. Under this model, the phenotypic covariance is

(Equation A5) $$\begin{array}{cc} \text{Cov}\left(Y_{k},X_{j}\right) & =\text{Cov}\left(G^{⊤}\beta_{Y_{k}}+\psi_{Y}U+\epsilon_{Y_{k}},G^{⊤}\beta_{X_{j}}+\psi_{X}U+\epsilon_{X_{j}}\right)\\ & =\beta_{Y_{k}}^{⊤}V\left(G\right)\beta_{X_{j}}+\psi_{Y}\psi_{X}V\left(U\right)+\text{Cov}\left(\epsilon_{Y_{k}},\epsilon_{X_{j}}\right) \end{array}$$

The phenotypic covariance can be decomposed into $c_{g}\left(Y_{k},X_{k}\right)=\text{Cov}\left(G^{⊤}\beta_{Y_{k}},G^{⊤}\beta_{X_{j}}\right)=\beta_{Y_{k}}^{⊤}V\left(G\right)\beta_{X_{j}}$, the genetic covariance between the two traits, i.e., the covariance between the genetic components of the two traits, $G^{⊤}\beta_{Y_{k}}$ and $G^{⊤}\beta_{X_{j}}$, and the environmental covariance, i.e., the covariance between the environmental effects of two traits that we have split into the effect of the unobserved confounder, $c_{u}\left(Y_{k},X_{k}\right)=\psi_{Y}\psi_{X}V\left(U\right)$, and other environmental factors, $c_{e}\left(Y_{k},X_{k}\right)=\text{Cov}\left(\epsilon_{Y_{k}},\epsilon_{X_{j}}\right)$. If the environmental factors are trait specific, since U includes all common confounding factors, $c_{e}\left(Y_{k},X_{k}\right)=0$ and Equation A5 shows that an estimand of the covariance between two traits unconfounded by U is $c_{g}$.

Assuming that the individuals for the two phenotypic traits are drawn from the same population with LD matrix between the genetic variants $V=G^{⊤}G$, the sampling distribution of the genetic effects are $N_{Y_{k}}^{1/2}\left({\hat{\beta }}_{Y_{k}}-\beta_{Y_{k}}\right)\overset{d}{\to }N_{n}\left(0,\sigma_{Y_{k}}^{2}V^{-1}\right)$ and $N_{X_{j}}^{1/2}\left({\hat{\beta }}_{X_{j}}-\beta_{X_{j}}\right)\overset{d}{\to }N_{n}\left(0,\sigma_{X_{j}}^{2}V^{-1}\right)$, where “d” denotes convergence in distribution. Under infinite sample sizes, ${\hat{\beta }}_{Y_{k}}\overset{p}{\to }\beta_{Y_{k}}$ and ${\hat{\beta }}_{X_{j}}\overset{p}{\to }\beta_{X_{j}}$, where “p” denotes convergence in probability, and an estimator of the genetic covariance between the two traits is

$${\hat{c}}_{g}\left(Y_{k},X_{j}\right)={\hat{\beta }}_{Y_{k}}^{⊤}V{\hat{\beta }}_{X_{j}.}$$

In the finite sample sizes case, the estimates of $\beta_{Y_{k}}$ and $\beta_{X_{j}}$ are noised and ${\hat{c}}_{g}\left(Y_{k},X_{j}\right)$ is biased^86^:

(Equation A6) $$E\left({\hat{c}}_{g}\left(Y_{k},X_{j}\right)\right)=\beta_{Y_{k}}^{⊤}V\beta_{X_{j}}+\frac{N_{o}\sum_{i=1}^{n}V_{ii}}{N_{Y_{k}}N_{X_{j}}}c_{u}\left(Y_{k},X_{j}\right)\text{,}$$

where $N_{o}$ is the sample size overlap between the two traits and n is the number of genetic variants considered. However, even in the scenario of complete overlap, the bias in Equation A6 is negligible if the sample sizes of the two traits are large, as usually happens in modern GWAS, and n is small compared to the sample sizes.

The same considerations can made for all phenotypic traits under investigation to reconstruct their joint genetic covariance unconfounded by U:

(Equation A7) $$\Sigma =\left[\begin{array}{cc} \Sigma_{YY} & \Sigma_{YX}\\ \Sigma_{XY} & \Sigma_{XX} \end{array}\right]=\left[\begin{array}{cc} B_{Y}^{⊤}V\left(G\right)B_{Y} & B_{Y}^{⊤}V\left(G\right)B_{X}\\ B_{X}^{⊤}V\left(G\right)B_{Y} & B_{X}^{⊤}V\left(G\right)B_{X} \end{array}\right]$$

where $\Sigma_{YY},\Sigma_{XX}$, and $\Sigma_{XY}$ are the genetic covariances within the outcomes, the exposures, and between them, and $B_{Y}$ and $B_{X}$ are the genetic effects on the outcomes and the exposures, respectively.

### MrDAG model

Assuming that the individuals for two phenotypic traits $Y_{k}$ and $X_{j}$ are drawn from the same population with LD matrix V, we have $N_{Y_{k}}^{1/2}\left(g^{⊤}{\hat{\beta }}_{Y_{k}}-g^{⊤}\beta_{Y_{k}}\right)\overset{d}{\to }N\left(0,\sigma_{Y_{k}}^{2}\right)$ and $N_{X_{j}}^{1/2}\left(g^{⊤}{\hat{\beta }}_{X_{j}}-g^{⊤}\beta_{X_{j}}\right)\overset{d}{\to }N\left(0,\sigma_{X_{j}}^{2}\right)$, where g are the selected IVs, $g^{⊤}{\hat{\beta }}_{Y_{k}}$ and $g^{⊤}{\hat{\beta }}_{X_{j}}$ are the kth and the jth genetically predicted values of the outcome and exposure, i.e., ${\hat{Y}}_{k}$ and ${\hat{X}}_{k}$, respectively.

The joint distribution of all genetically predicted values of the outcomes and exposures based on the IVs is

$${\left[g^{⊤}{\hat{B}}_{Y}g^{⊤}{\hat{B}}_{X}\right]}^{⊤}∼N_{q+p}\left({\left[g^{⊤}B_{Y}g^{⊤}B_{X}\right]}^{⊤},\Sigma \right)\text{,}$$

i.e., for large sample sizes they are normally distributed with mean ${\left[g^{⊤}B_{Y}g^{⊤}B_{X}\right]}^{⊤}$ and covariance matrix $\Sigma \in C_{D}$, the space of the symmetric positive definite covariance matrices Markov with respect to the DAG D.

If we assume that IVW is performed on the estimated genetic effects and IVs are independent after pruning or clumping, i.e., $V=I_{n}$, the MrDAG model becomes

(Equation A8) $${\left[g^{⊤}{\hat{B}}_{Y}^{*}g^{⊤}{\hat{B}}_{X}^{*}\right]}^{⊤}∼N_{q+p}\left({\left[g^{⊤}B_{Y}^{*}g^{⊤}B_{X}^{*}\right]}^{⊤},\Sigma^{*}\right)\text{,}$$

where ${\left[g^{⊤}{\hat{B}}_{Y}^{*}g^{⊤}{\hat{B}}_{X}^{*}\right]}^{⊤}={\left[g^{⊤}{\bar{\sigma }}_{Y}^{-1}{\hat{B}}_{Y}g^{⊤}{\bar{\sigma }}_{Y}^{-1}{\hat{B}}_{X}\right]}^{⊤}$ with ${\bar{\sigma }}_{Y}^{2}I_{n}=q^{-1}\sum_{k\in K}V\left({\hat{\beta }}_{Y_{k}}\right)$^2^ and similarly for ${\left[g^{⊤}B_{Y}^{*}g^{⊤}B_{X}^{*}\right]}^{⊤}$. The covariance matrix can be partitioned into

$$\Sigma^{*}=\left[\begin{array}{cc} \Sigma_{YY}^{*} & \Sigma_{YX}^{*}\\ \Sigma_{XY}^{*} & \Sigma_{XX}^{*} \end{array}\right],$$

where $\Sigma_{YY}^{*},\Sigma_{XX}^{*}$, and $\Sigma_{XY}^{*}$ are the genetic covariances within the outcomes, the exposures, and between them and its inverse (Harville,^87^ theorem 8.5.11) into

(Equation A9) $$\Omega^{*}=\Sigma^{{*}^{-1}}=\left[\begin{array}{cc} \Omega_{YY}^{*} & -\Omega_{YY}^{*}\Sigma_{YX}^{*}\Sigma_{XX}^{{*}^{-1}}\\ -\Sigma_{XX}^{{*}^{-1}}\Sigma_{XY}^{*}\Omega_{YY}^{*} & \Sigma_{XX}^{{*}^{-1}}+\Sigma_{XX}^{{*}^{-1}}\Sigma_{XY}^{*}\Omega_{YY}^{*}\Sigma_{YX}^{*}\Sigma_{XX}^{{*}^{-1}} \end{array}\right]\text{,}$$

with $\Omega^{*}\in P_{D}$, the space of the precision matrices Markov with respect to the DAG D and $\Omega_{YY}^{*}={\left(\Sigma_{YY}^{*}-\Sigma_{YX}^{*}\Sigma_{XX}^{{*}^{-1}}\Sigma_{XY}^{*}\right)}^{-1}$. However, since by partial ordering $\Omega_{YX}^{*}=\Omega_{YY}^{*}\Sigma_{YX}^{*}\Sigma_{XX}^{{*}^{-1}}=0$, Equation A9 becomes

$$\Omega^{*}=\left[\begin{array}{cc} \Omega_{YY}^{*} & 0\\ -\Sigma_{XX}^{{*}^{-1}}\Sigma_{XY}^{*}\Omega_{YY}^{*} & \Sigma_{XX}^{{*}^{-1}} \end{array}\right]\text{.}$$

By using $\Omega^{*}$, Gaussian graphical models^33^ can be used to estimate the conditional dependence relationships between the traits in the original (individual-level) data unconfounded by U, since genetically predicted outcomes and exposures depend only on the selected IVs.

Finally, for a given DAG D, the estimand of the causal effect under intervention^88^ is

(Equation A10) $$\theta_{h,k}={\left[\Sigma_{\text{fa}\left(h\right),\text{fa}\left(h\right)}^{{*}^{-1}}\Sigma_{\text{fa}\left(h\right),k}^{*}\right]}_{1}\text{,}$$

where $\Sigma_{\text{fa}\left(h\right),\text{fa}\left(h\right)}^{*}$ indicates the submatrix of $\Sigma^{*}$ whose rows and columns are $\text{fa}\left(h\right),\Sigma_{\text{fa}\left(h\right),k}^{*}$ indicates the subvector of $\Sigma^{*}$ whose rows are fa(h) and the columns correspond to the kth outcome, and where the subscript indicates the first element of the vector. By using Equation A7 and since $V\left(G\right)=I_{n}$, Equation A10 becomes

$$\theta_{h,k}={\left[{\left(B_{X_{\text{fa}\left(h\right)}}^{*⊤}B_{X_{\text{fa}\left(h\right)}}^{*}\right)}^{-1}B_{X_{\text{fa}\left(h\right)}}^{*⊤}\beta_{Y_{k}}^{*}\right]}_{1}\text{,}$$

where $B_{X_{\text{fa}\left(h\right)}}^{*}$ are the IVW coefficients of the regressions of each exposure in $X_{\text{fa}\left(h\right)}$ on G, and $\beta_{Y_{k}}^{*}$ is the IVW coefficient of a regression of $Y_{k}$ on G. The corresponding estimator coincides with Equation A4. As noted above, in contrast to general proposed solutions in MVMR, by conditioning on a given DAG, the set of regressors used in the estimator of the causal effect is with regard to the family of the exposure under intervention.

### MrDAG algorithm

#### Markov equivalent class, completed partial DAGs, essential graphs, and partial DAGs

The estimation of a DAG from observational data suffers the known problem of identifiability, i.e., it is not possible to estimate uniquely the underlying true DAG, since its conditional independencies can be encoded in several alternative DAGs. This set of DAGs that hold the same conditional independencies is known as Markov equivalent class (MEC), and the best that can be done from observational data is to estimate this class. All DAGs with the same conditional independencies can be represented by a completed partial DAG (CPDAG)^89^ or essential graph (EG).^35^ EGs are chain graphs (CGs) whose chain components are decomposable undirected graphs.^33^ A CPDAG or EG is a partially directed graph that might contain both directed and undirected edges without directed cycles. Finally, Partially DAGs (PDAGs) contain both directed and undirected edges, and directed cycles might be present.

#### Posterior probability of edge inclusion

In the following, we refer to ${\hat{Y}}^{*}=g^{⊤}{\hat{B}}_{Y}^{*}$ and ${\hat{X}}^{*}=g^{⊤}{\hat{B}}_{X}^{*}$, the genetically predicted values of the outcomes and exposures based on the genetic variants selected as IVs, as the “data.” Note that these quantities are not directly observable, but they are readily available as the product of the IVW summary-level statistics and the genotypes.

Technical details of the algorithm for graphical model exploration that we used to develop the MrDAG algorithm are presented in Castelletti et al.^90^ In brief, it is based on a MCMC algorithm devised to explore the space of EGs whose enumeration is infeasible, since their number grows super-exponentially with the number of nodes. The EG G is sampled from a proposal distribution described in He et al.^91^ Specifically, given EG G, six types of operators in the proposal distribution are considered: (1) inserting an undirected edge, (2) deleting an undirected edge, (3) inserting a directed edge, (4) deleting a directed edge, (5) converting two adjacent undirected edges in a v structure, and (6) converting a v structure in two adjacent undirected edges. Each operator $o_{G}$ determines the transition of G into another EG $G^{\prime }$ with the probability of transition from G to $G^{\prime }$ given by $p_{G,G^{\prime }}=1/\left|O_{G}\right|$, where $O_{G}$ is the set of operators that are allowed in the current EG G. The proposed EG $G^{\prime }$ is accepted with a probability given by a Metropolis-Hastings (M-H) ratio defined to guarantee the convergence of the algorithm to the correct posterior distribution

(Equation A11) $$\alpha =\min\left\{1,\frac{m_{G^{\prime }}\left(\text{data}\right)p\left(G^{\prime }\right)p_{G^{\prime },G}}{m_{G}\left(\text{data}\right)p\left(G\right)p_{G,G^{\prime }}}\right\}\text{,}$$

where p(G) is the prior on G (Equation A12) and $p_{G,G^{\prime }}$ is the transition probability defined above. The key ingredient of the M-H ratio is a closed-form expression for the marginal likelihood $m_{G}\left(\text{data}\right)$. For its computation, along with some hyper-parameters based on the data input (including the number of IVs), it requires the sufficient statistic for $\Sigma^{*}$ which is function of ${\hat{Y}}^{*}$ and ${\hat{X}}^{*}$. Since the IVs are chosen independently after pruning or clumping, the sufficient statistic becomes a function only of the summary-level data. We refer interested readers to Castelletti et al.^90^ for the derivation of the marginal likelihood.

In the MrDAG algorithm, we added an acceptance/rejection step to guarantee that in the proposed graph the partial ordering that corresponds to the orientation of the edges from the exposures to the outcomes is satisfied (see Figure 2E). Specifically, let $\left\{D_{l}\left(G\right);l=1,\ldots ,L_{G}\right\}$ denote the set of distinct DAGs in the MEC represented by G. The acceptance/rejection step checks that the partial ordering is fulfilled in the set of distinct DAGs. To check the efficiency of this step, we also monitor its acceptance rate. We also included a tempering scheme^92^ by considering an annealing parameter T in the M-H ratio to facilitate the convergence of the MCMC algorithm to the posterior distribution and the exploration of regions of high posterior mass. The temperature 1/T exponentiates the M-H ratio, and its value increases linearly during the burn-in until T=1 at the end of the burn-in.

Sparsity is enforced by assigning a prior to G and specifically on $G^{U}$, the skeleton of G, which contains the same edges of G but without orientation:

(Equation A12) $$G_{\left(l\right)}^{U}|\pi^{\text{edge}}\overset{i.i.d.}{∼}Ber\left(\pi^{\text{edge}}\right),l=1,\ldots ,\left(q+p\right)\left(q+p-1\right)/2\text{,}$$

where $G_{\left(l\right)}^{U}$ is the lth element of the vectorized lower triangular part of the adjacency matrix of $G^{U}$ and (q+p)(q+p−1)/2 is the maximum number of edges in an EG on q+p nodes.

The posterior distribution of G is

$$P\left(G|\text{data}\right)=\frac{m_{G}\left(\text{data}\right)P\left(G\right)}{\sum_{G\in S}m_{G}\left(\text{data}\right)P\left(G\right)}\text{,}$$

with S the set of all EGs with q+p nodes.

The posterior probability of edge inclusion (PPEI) is defined as

$$P_{z\to v}\left(\text{data}\right)=\sum_{G\in S_{z\to v}}P\left(G|\text{data}\right)\text{,}$$

where $S_{z\to v}$ is the set of EGs with q+p nodes containing the directed edge z→v.

Let $\left\{G^{s},s=1,\ldots ,S\right\}$ the set of visited EGs by MrDAG. The posterior probability of $G^{s}$ can be approximated by

(Equation A13) $$P\left(G^{s}|\text{data}\right)\approx \frac{m_{G^{s}}\left(\text{data}\right)P\left(G^{s}\right)}{\sum_{s=1}^{S}m_{G^{s}}\left(\text{data}\right)P\left(G^{s}\right)}$$

and, similarly, the PPEI by

(Equation A14) $$P_{z\to v}\left(\text{data}\right)\approx \sum_{s=1}^{S}I_{G\in S_{z\to v}}\left(G^{s}\in S_{z\to v}\right)P\left(G^{s}|\text{data}\right)\text{,}$$

where $I_{G}\in S_{z\to v}\left(G^{s}\in S_{z\to v}\right)$ is the indicator function that is equal to one if the visited EG belongs to the set of EGs with q+p nodes containing the directed edge z→v. Note that, although MrDAG explores the space of EGs, the graphs obtained by thresholding the PPEIs might give rise to a PDAG.^90^

### Bayesian causal effects estimation

Here, we summarize the results reported in Castelletti and Consonni^88^ that we employed to derive the Bayesian estimation of the causal effects under unobserved confounders.

Let us rewrite Equation A8 as

$${\left[g^{⊤}{\hat{B}}_{Y}^{*}g^{⊤}{\hat{B}}_{X}^{*}\right]}^{⊤}|\Sigma_{D}^{*}∼N_{q+p}\left({\left[g^{⊤}B_{Y}^{*}g^{⊤}B_{X}^{*}\right]}^{⊤},\Sigma_{D}^{*}\right)\text{,}$$

where $\Sigma_{D}^{*}\in C_{D}$, the space of s.p.d. (symmetric positive definite) covariance matrices Markov with respect to D. For ease of notation, in the following, we drop the subscript D.

Let $\Omega^{*}=\Sigma^{{*}^{-1}}=L^{*}D^{{*}^{-1}}L^{*⊤}$ be the modified Cholesky decomposition of the precision $\Omega^{*}$. The DAG Cholesky parameterization of $\Omega^{*}$ is given by the node-parameters $\omega_{l}^{*}=\left(D_{ll}^{*},L_{\text{fa}\left(l\right)}^{*}\right),l=1,\ldots ,q+p$, with

$$D_{ll}^{*}=\Sigma_{ll|\text{pa}\left(l\right)}^{*},L_{\text{fa}\left(l\right)}^{*}=-\Sigma_{\text{pa}\left(l\right)}^{{*}^{-1}}\Sigma_{\text{fa}\left(l\right)}^{*}\text{,}$$

where $\Sigma_{\text{pa}\left(l\right)}^{*}$ indicates the submatrix of $\Sigma^{*}$ whose rows and columns are pa(l).

For a given DAG D,^88^ derive the posterior distribution of $\omega_{l}^{*},l=1,\ldots ,q+p$, in an objective Bayes framework, which has the advantage of not depending on subjective priors hyper-parameters. In turn, the posterior draws of the Cholesky parameters $\omega_{l}^{*}$ provide posterior draws from $\left(\Omega^{*}|\text{data}\right)=\left(L^{*}D^{{*}^{-1}}L^{*⊤}|\text{data}\right)$ and, finally, by using Equation S9 for a given DAG, posterior samples of the causal effects between the exposures and the outcomes.

In contrast to frequentist approaches^50^^,^^84^ where, for an estimated EG G by the PC algorithm^53^ (or any other score-based method), the causal effects are calculated over all DAGs within the MEC G or, more efficiently, only “the unique possible” causal effects within a given EG G are provided, in this study we also consider the uncertainty related to the estimation of the EGs. Averaging over the visited EGs, the (Bayesian-model-averaged) causal effect under intervention in the hth exposures on the kth outcomes is

(Equation A15) $$\theta_{h,k}|\text{data}=\sum_{s=1}^{S}E\left(\theta_{h,k}\left(G^{s}\right)|\text{data},G^{s}\right)P\left(G^{s}|\text{data}\right),h\in J,k\in K\text{,}$$

where $G^{s}$ is an unique EGs visited during the MCMC, $E\left(\theta_{h,k}\left(G^{s}\right)|\text{data},G^{s}\right)$ is the posterior expectation of the causal effect given $G^{s},P\left(G^{s}|\text{data}\right)$ is defined in Equation A13. Note that $E\left(\theta_{h,k}\left(G^{s}\right)|\text{data},G^{s}\right)$ is implicitly defined over the distinct DAGs in the EG G. Specifically, let $\left\{D_{l}\left(G\right);l=1,\ldots ,L_{G}\right\}$ denote the set of distinct DAGs in the MEC represented by G. Then,

$$E\left(\theta_{h,k}\left(G^{s}\right)|\text{data},G^{s}\right)=\frac{1}{L_{G^{s}}}\sum_{l=1}^{L_{G^{s}}}E\left(\theta_{h,k}\left(D_{l}\left(G^{s}\right)\right)|\text{data},G^{s}\right)\text{,}$$

where $\theta_{h,k}\left(D_{l}\left(G^{s}\right)\right)|\text{data},G^{s}$ are posterior draws obtained during the MCMC. Finally, by a suitable modification of Equation A15, credible intervals of the causal effects between the exposures and outcomes can be derived.

### Detection of invalid IVs due to unmeasured pleiotropy

Detection of invalid IVs due to unmeasured pleiotropy in which some genetic variants are directly associated with a single outcome at a time and not via the exposures is performed by using the conditional predictive ordinate (CPO).^93^ The CPO is also known as the leave-one-out cross-validation predictive density,^94^ which is defined for the MrDAG model as

$$\begin{array}{ccc} {\text{CPO}}_{i} & = & m\left({\text{data}}_{i}|{\text{data}}_{-i}\right)\\ & = & \int_{S}m_{G}\left({\text{data}}_{i}\right)P\left(G|{\text{data}}_{-i}\right)dG\\ & = & E_{G|\text{data}}^{-1}\left(m_{G}^{-1}\left({\text{data}}_{i}\right)\right) \end{array}\text{,}$$

where $m_{G}\left({\text{data}}_{i}\right)$ is the marginal likelihood evaluated at the ith IV conditionally on the EG G and $P\left(G|{\text{data}}_{-i}\right)$ is the posterior probability of the EG G conditionally on all remaining IVs, with S the set of all EGs with q+p nodes. The CPO is based on the idea that is behind leave-one-out cross-validation: if the likelihood of observing an IV, having observed all the remaining IVs, is “small,” then the IV is considered an outlier and therefore an invalid IV.

An estimate of the CPO is based on the visited EGs during the MCMC. Specifically,

$${\hat{\text{CPO}}}_{i}=\frac{1}{\frac{1}{S}\sum_{s=1}^{S}m_{G^{s}}^{-1}\left({\text{data}}_{i}\right)}\text{,}$$

with $\left\{G^{s},s=1,\ldots ,S\right\}$ the set of EGs visited during the MCMC. Thus, the Monte Carlo estimate of ${\text{CPO}}_{i}$ is obtained without actually omitting the ith IV from the estimation of the posterior distribution of G and is provided by the harmonic mean of the marginal likelihood across the visited graphical models.

Recommendations are available regarding the threshold for the detection of outliers. Log-inverse-CPOs larger than 40 can be considered as possible outliers and higher than 70 as extreme values. Ntzoufras^95^^,^^96^ and Congdon^95^^,^^96^ recommend scaling CPOs by dividing each one by its maximum recorded across the MCMC sweeps and considering observations with scaled CPOs under 0.01 to be outliers. If few CPOs are less than 0.01, the model is considered to fit adequately. For the detection of outliers, we employ scaled CPO definition.

### Simulation study

We share several aspects of the simulation study with Zuber et al.^2^ It is formulated in a two-sample summary-level MR design, where N=100,000 independent individuals are simulated, of which $N_{Y}=50,000$ are used to compute the genetic associations with the exposures and $N_{X}=50,000$ to compute the genetic associations with the outcomes. Thus, we assume that the quantitative exposures $X_{j},j\in J=\left\{1,\ldots ,p\right\}$, and the quantitative responses $Y_{k},k\in K=\left\{1,\ldots ,q\right\}$, are measured on the same individuals $N_{X}$ and $N_{Y}$, respectively, with 100% sample overlap within the exposures and 100% sample overlap within the outcomes, but the samples are independent between the two groups, i.e., no sample overlap between $N_{X}$ and $N_{Y}$.

In all simulated scenarios, we consider p=15 exposures, q=5 outcomes, and n=100 independent genetic variants as IVs. Genetic variants for the ith genetic variant and each individual l are simulated independently according to a binomial distribution with minor allele frequency (MAF) equal to 0.05, i.e., $g_{l,i}\overset{i.i.d.}{∼}Bin\left(2,0.05\right),l\in L=\left\{1,\ldots ,N\right\},i\in I=\left\{1,\ldots ,n\right\}$. The resulting matrix of genetic variants G is split into two equally sized groups, $G_{X}$ and $G_{Y}$, of dimension $N_{X}\times n$ and $N_{Y}\times n$, respectively. Thus, no IVW is needed in the simulation study given that the same MAF at 5% is used to simulate the genetic variants.

Overall, the data-generation process consists of two stages. In the first stage, the raw data for the exposures X and the outcomes Y are simulated. Then, in the second stage, summary-level statistics are obtained as the linear regression coefficients ${\hat{\beta }}_{i,j}^{X}$ from a univariable linear regression in which the jth exposure is regressed on the ith genetic variant in sample 1 and the linear regression coefficients ${\hat{\beta }}_{i,k}^{Y}$ from a univariable linear regression in which the kth outcome is regressed on the ith genetic variant in sample 2.

In the following, we detail each stage and how we simulate the quantities involved. We start with the first stage which is divided into two steps.

(1) In the first step, the exposures are generated as follows:

(Equation A16) $$x_{j}=G_{X}\beta_{X_{j}}+\psi_{X}u_{X}+\epsilon_{X_{j}},j\in J\text{,}$$

where $G_{X}$ and $u_{X}$ are the genetic variants of the n IVs and the values of the confounder U measured on the same $N_{X}$ individuals, respectively, and where $\beta_{X_{j}}$ and $\psi_{X}$ are the corresponding genetic and confounding effects. $\epsilon_{X_{j}}∼N_{N_{X}}\left(0,h_{X_{j}}I_{N_{X}}\right)$, with $h_{X_{j}}$ the jth diagonal element of the (p×p)-dimensional matrix

(Equation A17) $$H_{X}=\frac{1-v_{X}}{v_{X}}{\left(G_{X}B_{X}+\psi_{X}u_{X}1_{p}^{⊤}\right)}^{⊤}\left(G_{X}B_{X}+\psi_{X}u_{X}1_{p}^{⊤}\right)\text{,}$$

where $v_{X}$ is the desired level of heritability, or how much variation G can explain of $X_{j}$, fixed at 10% for all exposures and in all simulated scenarios. In Equation A17, $B_{X}={\left\{\beta_{X_{j}}\right\}}_{j\in J}$ is an (n×p)-dimensional matrix of the effects of the genetic variants on the exposures.

The confounder U is drawn from a multi-variate standard Gaussian distribution, i.e., $u∼N_{N}\left(0,I_{N}\right)$ and then split into two equally sized vectors $u_{X}$ and $u_{Y}$, with effect $\psi_{X}$ impacting all exposures and $\psi_{Y}$ effecting all outcomes.

The effects $\beta_{X_{j}}$ of the n genetic variants on the jth exposure are drawn following Castelletti et al.^90^ We randomly generate a topologically ordered DAG among the p exposures with a probability of edge inclusion $p_{X}^{\text{edge}}=2/\left(p-1\right)$ using the function randomDAG() in the R package *pcalg*.^50^ Thus, the resulting DAG implies the following system of equations^34^:

(Equation A18) $$\beta_{X_{j}}=\sum_{h\in \text{pa}\left(j\right)}\gamma_{h,j}^{X}\beta_{X_{h}}+\epsilon_{X_{j}}$$

with $\epsilon_{X_{j}}∼N\left(0,I_{n}\right)$. For each j∈J, the effect within the exposures $\gamma_{h,j}^{X}$ are uniformly chosen in the interval $\left[-1.1r_{X},-0.9r_{X}\right]\cup \left[0.9r_{X},1.1r_{X}\right]$. This construction procedure for $\beta_{X_{j}}$ corresponds to the simulated scenario that we call “${\text{DAG}}_{X}$,” i.e., DAG within X, which, in turn, is paired with two different simulated scenarios for the effects $\beta_{Y_{k}}$ described in the second step (first stage) of the simulation study.

We also simulate the effects $\beta_{X_{j}}$ following Zuber et al.^2^ Specifically, we simulate $\beta_{X_{j}}∼N_{n}\left(0,R_{X}\right)$, where $R_{X}$ is the (p×p)-dimensional Toeplitz matrix with $r_{X}^{\left|j-j^{\prime }\right|}$ for $j,j^{\prime }\in J$. The matrix $R_{X}$ implies a tridiagonal sparse inverse correlation matrix $\Omega_{X}=R_{X}^{-1}$. The interpretation of non-zero elements of $\Omega_{X}$ coincides with the effects simulated in Equation A18. We call this second scenario for the effects of the genetic variants on the exposures “${\text{UndG}}_{X}$,” i.e., undirected graph within X.

In both simulated scenarios for X we use different levels of $r_{X}$, ranging from independence to a strong dependence, i.e., $r_{X}=\left\{0,0.2,0.4,0.6,0.8\right\}$, where $r_{X}=0.6$ represents a medium dependence between the genetic associations with the exposures. We use this value in the figures presented in the ”simulation study” section.

(2) In the second step (first stage) of the simulation study, the outcomes are generated on another independent set of $N_{Y}$ individuals based on the following set of equations:

(Equation A19) $$y_{k}=X\theta_{k}+\sum_{h\in \text{pa}\left(k\right)}\gamma_{h,k}^{Y}y_{h}+\psi_{Y}u+\epsilon_{Y_{k}},k\in K\text{,}$$

where X is the $\left(N_{X}\times p\right)$-dimensional matrix of exposures simulated using Equation A16, $\theta_{k}={\left(\theta_{1k},\ldots ,\theta_{pk}\right)}^{⊤}$ is p-dimensional (sparse) vector the causal effects from the exposures to the kth outcome and where $\psi_{Y}$ is the effect of the confounder U on the outcomes. $\epsilon_{Y_{k}}∼N_{N_{Y}}\left(0,h_{Y_{k}}I_{N_{Y}}\right)$, with $h_{Y_{k}}$ the kth diagonal element of the (q×q)-dimensional matrix

$$\begin{array}{cc} H_{Y} & =\frac{1-v_{Y}}{v_{Y}}{\left(X\theta_{k}+\sum_{h\in \text{pa}\left(k\right)}\gamma_{h,k}^{Y}y_{h}+\psi_{Y}u+\varepsilon_{Y_{k}}\right)}^{⊤}\\ & \;\left(X\theta_{k}+\sum_{h\in \text{pa}\left(k\right)}\gamma_{h,k}^{Y}y_{h}+\psi_{Y}u+\varepsilon_{Y_{k}}\right) \end{array}\text{,}$$

where $v_{Y}$ is the desired level of the proportion of variance explained, fixed at 25% for all outcomes and in all simulated scenarios.

In Equation A19, the term $\sum_{h\in \text{pa}\left(k\right)}\gamma_{h,k}^{Y}Y_{h}$ depends on a randomly generated topologically ordered DAG among the q outcomes with probability of edge inclusion $p_{Y}^{\text{edge}}=1/\left(q-1\right)$. For each k∈K, the effects within the outcomes $\gamma_{h,k}^{Y}$ are uniformly drawn in the interval $\left[0.9m_{Y},1.1m_{Y}\right]$. In analogy with the first step, we call this scenario “${\text{DAG}}_{Y}$,” i.e., DAG within Y.

We also simulate a simplified scenario where

(Equation A20) $$y_{k}=\gamma_{h,k}^{Y}y_{h}+\psi_{Y}u+\epsilon_{Y_{k}}\text{,}$$

i.e., a randomly selected outcome k is completed mediated by another randomly selected response chosen between the remaining ones. We call this scenario “${\text{Med}}_{Y}$,” i.e., complete mediation of an outcome, since in the previous scenario “${\text{DAG}}_{Y}$” partial mediations^97^ are likely simulated, while here we exclude this case. In this second simulated scenario for the outcomes, the matrix $H_{Y}$ is calculated according to Equation A20. Moreover, we use different levels of $m_{Y}$, ranging from small to a strong level of (partial or complete) mediation, i.e., $m_{Y}=\left\{0.25,0.50,0.75,1,1.5,2\right\}$, where $m_{Y}=1$ represents a medium (partial or complete) mediation effect. We use this value in the figures presented in the section “simulation study.”

Finally, the causal effects $\theta_{k}$ are drawn independently from a multi-variate Gaussian distribution, i.e., $\theta_{k}∼N_{p}\left(0,I_{p}\right)$.

In both simulated scenarios for Y, we consider a (q×p)-dimensional sparse matrix of causal effects $\Theta ={\left\{\theta_{k}\right\}}_{k\in K}$, where 30 cells of the matrix are non-zero and where several exposures are either shared or distinct for the outcomes. Specifically, we select at random the same proportion of cells in the matrix Θ and assign them the simulated values, while the other cells are set to zero.

After the first stage, four scenarios are created by combining the simulations for X and Y: (1) “${\text{UndG}}_{X}$-${\text{Med}}_{Y}\text{,}$” i.e., undirected graph within X and complete mediation of an outcome in Y; (2) “${\text{DAG}}_{X}$-${\text{Med}}_{Y}$,” i.e., topologically ordered DAG within X and complete mediation of a response within Y; (3) “${\text{UndG}}_{X}$-${\text{DAG}}_{Y}$,” i.e., undirected graph within X and topologically ordered DAG within Y; and (4) “${\text{DAG}}_{X}$-${\text{DAG}}_{Y}$,” topologically ordered DAGs within X and Y. In (2) and (4) the overall DAGs, obtained by combining different simulation patterns for X and Y, are fully oriented, while in (1) and (3) they are partially oriented.

After creating the data at the individual level, in the second stage, we compute the summary-level statistics from the two independent groups of individuals. The input data for the simulation study are the summary-level statistics ${\hat{B}}_{X}={\left\{{\hat{\beta }}_{i,j}^{X}\right\}}_{i\in I,j\in J}$, an (n×p)-dimensional matrix, and ${\hat{B}}_{Y}={\left\{{\hat{\beta }}_{i,k}^{Y}\right\}}_{i\in I,k\in K}$, an (n×q)-dimensional matrix, derived from a univariable linear regression model, where each genetic variant $G_{i}$ is regressed against each exposure $X_{j}$ and each outcome $Y_{k}$, one at a time.

### Real data application: Pre-processing and data preparation

The first step of the data processing merges the summary-level data (beta regression coefficients, their standard errors, and associated p values) of all exposures by their unique “rs” identifier and aligns the effect direction of the genetic associations with each exposure according to the same effect allele. As IVs, we select the genetic variants that are associated with any of the exposures at genome-wide significance (minimum p value <5 × 10^−8^ across all exposures). Next, we merge the genetic variants selected as IVs with the outcome data by their unique “rs” identifier and align the effect direction of the genetic associations with each outcome according to the same effect allele. Finally, we clump the genetic variants to be independent at $r^{2}<0.01$ using a European reference panel.^30^ This results in n=708 independent genetic variants selected as IVs. See Table S1 for the description of the summary-level statistics, the data sources, and the number of non-unique IVs that were genome-wide significant for each exposure, along with the contribution (%) of each exposure on the selected IVs.

Finally, we perform reverse causation using the same traits with mental health phenotypes as exposures and lifestyle and behavioral traits as outcomes. We apply the same procedure described above, resulting in 470 IVs for mental health phenotypes. See Table S1 for details regarding the number of non-unique IVs that were genome-wide significant for each exposure along with the contribution (%) of each exposure on the selected IVs.

## Supplemental information

Document S1. Supplemental text, Figures S1–S27, and Tables S1–S3

Document S2. Article plus supplemental information
