# Supplementary material for: Bayesian causal graphical model for joint Mendelian randomization analysis of multiple exposures and outcomes
Source: Am J Hum Genet. 2025 Apr 2;112(5):1173–98. doi: 10.1016/j.ajhg.2025.03.005 (PMC12120189; doi:10.1016/j.ajhg.2025.03.005)
Supplement: Document S1. Supplemental text, Figures S1–S27, and Tables S1–S3 [file mmc1.pdf]

The American Journal of Human Genetics, Volume *112*

Supplemental information

Bayesian causal graphical model for joint Mendelian randomization analysis of multiple exposures and outcomes

Verena Zuber, Toinét Cronjé, Na Cai, Dipender Gill, and Leonardo Bottolo

S1 Supplemental Text

In the following, we assume that the conditional expectation of the outcome Y_k, $k \in K$, and the conditional expectation of the exposure X_j, $j \in J$, are linear with regard to their DAG parents without interactions and all dependencies only affect the mean. These assumptions lead to

$$\mathbb{E}(Y_k \mid \boldsymbol{Y}_{\mathrm{pa}(k)} = \boldsymbol{y}_{\mathrm{pa}(k)}, \boldsymbol{X}_{\mathrm{pa}(k)} = \boldsymbol{x}_{\mathrm{pa}(k)}, U = u) = \sum_{j \in \mathrm{pa}(k)} \theta_{j,k} x_j + \sum_{h \in \mathrm{pa}(k)} \gamma^Y_{h,k} y_h + \psi_Y u,$$
$$\mathbb{E}(X_j \mid \boldsymbol{X}_{\mathrm{pa}(j)} = \boldsymbol{x}_{\mathrm{pa}(j)}, \boldsymbol{G} = \boldsymbol{g}, U = u) = \sum_{i \in I} \beta^X_{i,j} g_i + \sum_{h \in \mathrm{pa}(j)} \gamma^X_{h,j} x_h + \psi_X u,$$

where $\mathrm{pa}_{\mathcal{D}}(v)$ denotes the parent set of the node v in the DAG $\mathcal{D}$ (in the following, for ease of notation, we remove the subscript $\mathcal{D}$), $\gamma^Y_{h,k}$ is the effect of the hth outcomes on the kth outcome, $h \neq k$, $\theta_{j,k}$, is the causal effect of the jth exposure on the kth outcome and $\gamma^X_{h,j}$ is the effect of the hth exposure on the jth exposure, $h \neq j$.

In addition, we assume that the expectation of Y_k conditionally on an intervention on X_h, $h \in \mathrm{pa}(k)$, is

$$\mathbb{E}(Y_k \mid \mathrm{do}(X_h = \widetilde{x}_h), \boldsymbol{X}_{j \in \mathrm{pa}(h)} = \boldsymbol{x}_{j \in \mathrm{pa}(h)}, U = u) = \theta_{h,k} \widetilde{x}_h + \sum_{j \in \mathrm{pa}(h)} \theta_{j,k} x_j + \psi_Y u. \quad \text{(S1)}$$

Regressions of outcomes and exposures on $\boldsymbol{G}$

The proof that, for a given DAG $\mathcal{D}$, the effects of $\mathbb{E}(Y_k \mid \boldsymbol{G} = \boldsymbol{g})$, $k \in K$, and $\mathbb{E}(X_j \mid \boldsymbol{G} = \boldsymbol{g})$, $j \in J$, can be obtained without adjustment on U because $\boldsymbol{G}$ is randomly assigned at conception[1], as shown for standard MR[2], is provided in the following proposition. The proof depends on the multivariate "core conditions" (MCC) for valid IVs presented in the main text Appendix, the Markov properties (MP) of the DAG and the different expectations being additive in the conditioning variables. For $\mathbb{E}(Y_k \mid \boldsymbol{G} = \boldsymbol{g})$, we compute the conditional expectation of Y_k given $(\boldsymbol{Y}_{\mathrm{pa}(k)}, \boldsymbol{X}_{\mathrm{pa}(k)}, U)$, then (for each outcome in $\boldsymbol{Y}_{\mathrm{pa}(k)}$ we proceed similarly) integrate out each exposure in $\boldsymbol{X}_{\mathrm{pa}(k)}$ with respect to its conditional distribution given $(\boldsymbol{G} = \boldsymbol{g}, U)$ until there are no exposures that have other exposures as parents and, finally, integrate out the unobserved confounder U with regard to its marginal distribution. We use the same strategy to compute the conditional expectation of X_j given $\boldsymbol{G}$.

Proposition S1. For a given DAG $\mathcal{D}$ that represents the conditional dependencies between $\boldsymbol{Y}$, $\boldsymbol{X}$, $\boldsymbol{G}$ and U, $\mathbb{E}(Y_k \mid \boldsymbol{G} = \boldsymbol{g})$, $\forall k \in K$, and $\mathbb{E}(X_j \mid \boldsymbol{G} = \boldsymbol{g})$, $\forall j \in J$, are unconfounded by U.

Proof. Let's assume that the multivariate extensions of the "core conditions" for a valid instrumental variable hold:

(IV1) $G_i \perp\!\!\!\perp U$, $\forall i \in I$, *i.e.*, G_i must be independent of U;

(IV2) $G_i \not\!\perp\!\!\!\perp X_j \mid \boldsymbol{X}_{\setminus j}$, $\forall i \in I$ and $\forall j \in J$, *i.e.*, G_i must *not* be independent of X_j conditionally on $\boldsymbol{X}_{\setminus j}$;

(IV3) $G_i \perp\!\!\!\perp Y_k \mid (\boldsymbol{X}, U)$, $\forall i \in I$ and $\forall k \in K$, *i.e.*, G_i must be independent of Y_k conditionally on $\boldsymbol{X}$ and U.

For a given DAG, let's assume that, among the outcomes and exposures, the parents of Y_k are $\boldsymbol{Y}_{\mathrm{pa}(k)}$ and $\boldsymbol{X}_{\mathrm{pa}(k)}$, $\boldsymbol{Y}_{\mathrm{pa}(k)}$ do not have any outcomes among their parents and $\boldsymbol{X}_{\mathrm{pa}(k)}$ have parents among the exposures. We denote the parents of $\boldsymbol{X}_{\mathrm{pa}(k)}$ as $\boldsymbol{X}_{\mathrm{pa}(\mathrm{pa}(k))}$ which, in turn, do not have any exposures among their parents. We can write the conditional expectation of Y_k given $\boldsymbol{G}$ as

$$\begin{aligned}
\mathbb{E}(Y_k \mid \boldsymbol{G}=\boldsymbol{g}) &= \mathbb{E}_{U|G=g}\, \mathbb{E}_{Y_{\mathrm{pa}(k)}, X_{\mathrm{pa}(k)}, X_{\mathrm{pa}(\mathrm{pa}(k))}|G=g,U} \\
&\quad \mathbb{E}(Y_k \mid \boldsymbol{Y}_{\mathrm{pa}(k)}, \boldsymbol{X}_{\mathrm{pa}(k)}, \boldsymbol{X}_{\mathrm{pa}(\mathrm{pa}(k))}, \boldsymbol{G}=\boldsymbol{g}, U) \\
&= \mathbb{E}_{U|G=g}\, \mathbb{E}_{X_{\mathrm{pa}(\mathrm{pa}(k))}|G=g,U}\, \mathbb{E}_{X_{\mathrm{pa}(k)}|X_{\mathrm{pa}(\mathrm{pa}(k))},G=g,U}\, \mathbb{E}_{Y_{\mathrm{pa}(k)}|X_{\mathrm{pa}(k)},X_{\mathrm{pa}(\mathrm{pa}(k))},G=g,U} \\
&\quad \mathbb{E}(Y_k \mid \boldsymbol{Y}_{\mathrm{pa}(k)}, \boldsymbol{X}_{\mathrm{pa}(k)}, \boldsymbol{X}_{\mathrm{pa}(\mathrm{pa}(k))}, \boldsymbol{G}=\boldsymbol{g}, U) \\
&= \mathbb{E}_{U|G=g}\, \mathbb{E}_{X_{\mathrm{pa}(\mathrm{pa}(k))}|G=g,U}\, \mathbb{E}_{X_{\mathrm{pa}(k)}|X_{\mathrm{pa}(\mathrm{pa}(k))},G=g,U} \\
&\quad \mathbb{E}_{Y_{\mathrm{pa}(k)}|X_{\mathrm{pa}(k)},X_{\mathrm{pa}(\mathrm{pa}(k))},U}\, \mathbb{E}(Y_k \mid \boldsymbol{Y}_{\mathrm{pa}(k)}, \boldsymbol{X}_{\mathrm{pa}(k)}, \boldsymbol{G}=\boldsymbol{g}, U) \text{ since MP} \\
&= \mathbb{E}_U\, \mathbb{E}_{X_{\mathrm{pa}(\mathrm{pa}(k))}|G=g,U}\, \mathbb{E}_{Y_{\mathrm{pa}(k)}|X_{\mathrm{pa}(k)},X_{\mathrm{pa}(\mathrm{pa}(k))},U} \\
&\quad \mathbb{E}_{X_{\mathrm{pa}(k)}|X_{\mathrm{pa}(\mathrm{pa}(k))},G=g,U}\, \mathbb{E}(Y_k \mid \boldsymbol{Y}_{\mathrm{pa}(k)}, \boldsymbol{X}_{\mathrm{pa}(k)}, U) \text{ since IV1 and IV3} \\
&= \mathbb{E}_U\, \mathbb{E}_{X_{\mathrm{pa}(\mathrm{pa}(k))}|G=g,U}\, \mathbb{E}_{Y_{\mathrm{pa}(k)}|X_{\mathrm{pa}(k)},X_{\mathrm{pa}(\mathrm{pa}(k))},U} \\
&\quad \mathbb{E}_{X_{\mathrm{pa}(k)}|X_{\mathrm{pa}(\mathrm{pa}(k))},G=g,U}\Big(\sum_{j\in\mathrm{pa}(k)} \theta_{j,k} X_j + \sum_{h\in\mathrm{pa}(k)} \gamma^Y_{h,k} Y_h + \psi_Y U\Big) \\
&= \sum_{j\in\mathrm{pa}(k)} \theta_{j,k} \underbrace{\mathbb{E}_U\, \mathbb{E}_{X_{\mathrm{pa}(j)}|G=g,U}\, \mathbb{E}_{X_j|X_{\mathrm{pa}(j)}G=g,U}\, X_j}_{T_1} + \\
&\quad \sum_{h\in\mathrm{pa}(k)} \gamma^Y_{h,k} \underbrace{\mathbb{E}_U\, \mathbb{E}_{X_{\mathrm{pa}(h)}|G=g,U}\, \mathbb{E}_{Y_h|X_{\mathrm{pa}(h)},U}\, Y_h}_{T_2} + \psi_Y\, \mathbb{E}\, U.
\end{aligned} \tag{S2}$$

Under the assumptions regarding the parents of $\boldsymbol{Y}_{\mathrm{pa}(k)}$ and $\boldsymbol{X}_{\mathrm{pa}(\mathrm{pa}(k))}$, we have

$$\begin{aligned}
T_1 &= \mathbb{E}_U\, \mathbb{E}_{X_{\mathrm{pa}(j)}|G=g,U}\Big(\sum_{s\in\mathrm{pa}(j)} \gamma^X_{s,j} X_s + \boldsymbol{g}^\top \boldsymbol{\beta}_{X_j} + \psi_X U\Big) \\
&= \boldsymbol{g}^\top \boldsymbol{\beta}_{X_j} + \sum_{s\in\mathrm{pa}(j)} \gamma^X_{s,j}\, \mathbb{E}_U\, \mathbb{E}_{X_s|G=g,U}\, X_s + \psi_X\, \mathbb{E}\, U \\
&= \boldsymbol{g}^\top \boldsymbol{\beta}_{X_j} + \sum_{s\in\mathrm{pa}(j)} \gamma^X_{s,j}(\boldsymbol{g}^\top \boldsymbol{\beta}_{X_s} + \psi_X\, \mathbb{E}\, U) + \psi_X\, \mathbb{E}\, U \\
&= \boldsymbol{g}^\top \boldsymbol{\beta}_{X_j} + \sum_{s\in\mathrm{pa}(j)} \gamma^X_{s,j} \boldsymbol{g}^\top \boldsymbol{\beta}_{X_s} + \{(1 + \sum_{s\in\mathrm{pa}(j)} \gamma^X_{s,j})\}\psi_X\, \mathbb{E}\, U
\end{aligned} \tag{S3}$$

and

$$\begin{aligned}
T_2 &= \mathbb{E}_U\, \mathbb{E}_{X_{\mathrm{pa}(h)}|G=g,U}\Big(\sum_{r\in\mathrm{pa}(h)} \theta_{r,h} X_r + \psi_Y U\Big) \\
&= \sum_{r\in\mathrm{pa}(h)} \theta_{r,h}\, \mathbb{E}_U\, \mathbb{E}_{X_r|G=g,U}\, X_r + \psi_Y\, \mathbb{E}\, U \\
&= \sum_{r\in\mathrm{pa}(h)} \theta_{r,h}(\boldsymbol{g}^\top \boldsymbol{\beta}_{X_r} + \psi_X\, \mathbb{E}\, U) + \psi_Y\, \mathbb{E}\, U \\
&= \sum_{r\in\mathrm{pa}(h)} \theta_{r,h} \boldsymbol{g}^\top \boldsymbol{\beta}_{X_r} + \Big(\sum_{r\in\mathrm{pa}(h)} \theta_{r,h}\psi_X + \psi_Y\Big)\, \mathbb{E}\, U.
\end{aligned} \tag{S4}$$

Combining (S2), (S3) and (S4), we obtain

$$\begin{aligned}\mathbb{E}(Y_k \mid \boldsymbol{G}=\boldsymbol{g}) = \alpha_{Y_k} + \sum_{j\in \mathrm{pa}(k)} \theta_{j,k}\boldsymbol{g}^\top\boldsymbol{\beta}_{X_j} + \sum_{j\in \mathrm{pa}(k)} \theta_{j,k} \sum_{s\in \mathrm{pa}(j)} \gamma^X_{s,j}\boldsymbol{g}^\top\boldsymbol{\beta}_{X_s} + \\ \sum_{h\in \mathrm{pa}(k)} \gamma^Y_{h,k} \sum_{r\in \mathrm{pa}(h)} \theta_{r,h}\boldsymbol{g}^\top\boldsymbol{\beta}_{X_r},\end{aligned} \tag{S5}$$

where $\sum_{j\in \mathrm{pa}(k)} \theta_{j,k}\boldsymbol{g}^\top\boldsymbol{\beta}_{X_j}$, $\sum_{j\in \mathrm{pa}(k)} \theta_{j,k} \sum_{s\in \mathrm{pa}(j)} \gamma^X_{s,j}\boldsymbol{g}^\top\boldsymbol{\beta}_{X_s}$ and $\sum_{h\in \mathrm{pa}(k)} \gamma^Y_{h,k} \sum_{r\in \mathrm{pa}(h)} \theta_{r,h}\boldsymbol{g}^\top\boldsymbol{\beta}_{X_r}$ are the genetic components of Y_k determined by the genetic components of the exposures among its parents and mediated by other exposures and outcomes. Finally, α_{Y_k} captures all terms that are related to $\mathbb{E}\,U$.

Similarly, assuming that $\boldsymbol{X}_{\mathrm{pa}(\mathrm{pa}(j))}$ do have parents among the exposures, we have

$$\begin{aligned}\mathbb{E}(X_j \mid \boldsymbol{G}=\boldsymbol{g}) &= \mathbb{E}_{U|G=g}\, \mathbb{E}_{X_{\mathrm{pa}(\mathrm{pa}(j))}|G=g,U}\, \mathbb{E}_{X_{\mathrm{pa}(j)}|X_{\mathrm{pa}(\mathrm{pa}(j))},G=g,U} \\ &\quad \mathbb{E}(X_j \mid \boldsymbol{X}_{\mathrm{pa}(j)}, \boldsymbol{X}_{\mathrm{pa}(\mathrm{pa}(j))}, \boldsymbol{G}=\boldsymbol{g}, U) \\ &= \mathbb{E}_{U|G=g}\, \mathbb{E}_{X_{\mathrm{pa}(\mathrm{pa}(j))}|G=g,U}\, \mathbb{E}_{X_{\mathrm{pa}(j)}|X_{\mathrm{pa}(\mathrm{pa}(j))},G=g,U} \\ &\quad \mathbb{E}(X_j \mid \boldsymbol{X}_{\mathrm{pa}(j)}, \boldsymbol{G}=\boldsymbol{g}, U) \text{ since MP} \\ &= \mathbb{E}_U\, \mathbb{E}_{X_{\mathrm{pa}(\mathrm{pa}(j))}|G=g,U}\, \mathbb{E}_{X_{\mathrm{pa}(j)}|X_{\mathrm{pa}(\mathrm{pa}(j))},G=g,U} (\boldsymbol{g}^\top\boldsymbol{\beta}_{X_j} + \sum_{r\in \mathrm{pa}(j)} \gamma^X_{r,j} X_r + \psi_X U) \\ &= \boldsymbol{g}^\top\boldsymbol{\beta}_{X_j} + \sum_{r\in \mathrm{pa}(j)} \gamma^X_{r,j}\, \mathbb{E}_U\, \mathbb{E}_{X_{\mathrm{pa}(r)}|G=g,U}\, \mathbb{E}_{X_r|X_{\mathrm{pa}(r)},G=g,U}\, X_r + \psi_X\, \mathbb{E}\,U \\ &\quad \text{since IV1} \\ &= \boldsymbol{g}^\top\boldsymbol{\beta}_{X_j} + \sum_{r\in \mathrm{pa}(j)} \gamma^X_{r,j}\, \mathbb{E}_U\, \mathbb{E}_{X_{\mathrm{pa}(r)}|G=g,U} (\boldsymbol{g}^\top\boldsymbol{\beta}_{X_r} + \sum_{s\in \mathrm{pa}(r)} \gamma^X_{s,r} X_s + \psi_X U) + \\ &\quad \psi_X\, \mathbb{E}\,U \\ &= \boldsymbol{g}^\top\boldsymbol{\beta}_{X_j} + \sum_{r\in \mathrm{pa}(j)} \gamma^X_{r,j} (\boldsymbol{g}^\top\boldsymbol{\beta}_{X_r} + \sum_{s\in \mathrm{pa}(r)} \gamma^X_{s,r}\, \mathbb{E}_U\, \mathbb{E}_{X_s|G=g,U}\, X_s + \psi_X U) + \\ &\quad \psi_X\, \mathbb{E}\,U \\ &= \boldsymbol{g}^\top\boldsymbol{\beta}_{X_j} + \sum_{r\in \mathrm{pa}(j)} \gamma^X_{r,j} \{\boldsymbol{g}^\top\boldsymbol{\beta}_{X_r} + \sum_{s\in \mathrm{pa}(r)} \gamma^X_{s,r} (\boldsymbol{g}^\top\boldsymbol{\beta}_{X_s} + \psi_X\, \mathbb{E}\,U) + \psi_X\, \mathbb{E}\,U\} + \\ &\quad \psi_X\, \mathbb{E}\,U \\ &= \alpha_{X_j} + \boldsymbol{g}^\top\boldsymbol{\beta}_{X_j} + \sum_{r\in \mathrm{pa}(j)} \gamma^X_{r,j}\boldsymbol{g}^\top\boldsymbol{\beta}_{X_r} + \sum_{r\in \mathrm{pa}(j)} \gamma^X_{r,j} \sum_{s\in \mathrm{pa}(r)} \gamma^X_{s,r}\boldsymbol{g}^\top\boldsymbol{\beta}_{X_s},\end{aligned} \tag{S6}$$

where $\boldsymbol{g}^\top\boldsymbol{\beta}_{X_j}$, $\sum_{r\in \mathrm{pa}(j)} \gamma^X_{r,j}\boldsymbol{g}^\top\boldsymbol{\beta}_{X_r}$ and $\sum_{r\in \mathrm{pa}(j)} \gamma^X_{r,j} \sum_{s\in \mathrm{pa}(r)} \gamma^X_{s,r}\boldsymbol{g}^\top\boldsymbol{\beta}_{X_s}$ are the direct and mediated genetic components of X_j which, in turn, are function of the genetic components of other exposures. Finally, α_{X_j} captures all terms that are related to $\mathbb{E}\,U$. ■

Remark S1. In (S2) and (S6), we made some assumptions regarding the dependency structure of the parents of $\boldsymbol{Y}_{\mathrm{pa}(k)}$ and $\boldsymbol{X}_{\mathrm{pa}(\mathrm{pa}(k))}$, and the parents of $\boldsymbol{X}_{\mathrm{pa}(\mathrm{pa}(j))}$, respectively. The proof does not change if further dependency relations are considered among the outcomes and the exposures given that the expectations are additive in the conditioning variables and each exposure can be integrated out with regard to its conditional distribution. Under these assumptions, regardless of the dependency structure associated

with a given DAG, it is possible to integrate out U with regard to its marginal distribution.

Remark S2. In (S5), the regression coefficient of a regression of Y_k on $\boldsymbol{G}$ is "consistent" for the causal parameters of interest as well as the mediation parameters within the outcomes and the exposures. In (S6), the regression coefficient of a regression of X_j on $\boldsymbol{G}$ yields a consistent estimate of the mediation parameters within the exposures.

Identification and estimation of the causal effects

In this section, we first specify what the causal parameters of interest are, and second determine how they relate to the parameters of a regression of Y_k on $\boldsymbol{G}$ (S2) and a regression of X_j on $\boldsymbol{G}$ (S6).

To prove that

$$\theta_{h,k} = \frac{\partial}{\partial x_h} \mathbb{E}(Y_k \mid \mathrm{do}(X_h = x_h))\bigg|_{x_h=\tilde{x}_h}, \tag{S7}$$

we assume general linear models for the dependencies among the variables $\boldsymbol{Y}$, $\boldsymbol{X}$, $\boldsymbol{G}$ and U and compute the conditional expectation of Y_k given $(\boldsymbol{X}_{\mathrm{pa}(h)}, U)$. Then, we remove the conditioning on $\mathrm{do}(X_h = x_h)$ since there cannot be dependence between any random variables and an intervention, integrate out each exposure in $\boldsymbol{X}_{\mathrm{pa}(h)}$ with respect to its conditional distribution and, finally, integrate out $\boldsymbol{G}$ and U with regard to their marginal distributions.

Proposition S2. For a given DAG $\mathcal{D}$ that represents the conditional dependencies between $\boldsymbol{Y}$, $\boldsymbol{X}$, $\boldsymbol{G}$ and U, the causal effect of an intervention in $X_h = \tilde{x}_h$ on Y_k is $\theta_{h,k}$.

Proof. Let's assume MCC and model (S1) hold. To show (S7), it is sufficient to prove that

$$\begin{aligned}
\mathbb{E}(Y_k \mid \mathrm{do}(X_h = x_h)) &= \mathbb{E}_{X_{\mathrm{pa}(h)},U|\mathrm{do}(X_h=x_h)}\, \mathbb{E}(Y_k \mid \mathrm{do}(X_h = x_h), \boldsymbol{X}_{\mathrm{pa}(h)}, U) \\
&= \mathbb{E}_{U|\mathrm{do}(X_h=x_h)}\, \mathbb{E}_{X_{\mathrm{pa}(h)}|\mathrm{do}(X_h=x_h),U}\, \mathbb{E}(Y_k \mid \mathrm{do}(X_h = x_h), \boldsymbol{X}_{\mathrm{pa}(h)}, U) \\
&= \mathbb{E}_U\, \mathbb{E}_{X_{\mathrm{pa}(h)}|U}\, \mathbb{E}(Y_k \mid \mathrm{do}(X_h = x_h), \boldsymbol{X}_{\mathrm{pa}(h)}, U) \\
&= \mathbb{E}_U\, \mathbb{E}_{X_{\mathrm{pa}(h)}|U} (\theta_{h,k} x_h + \textstyle\sum_{j\in\mathrm{pa}(h)} \theta_{j,k} X_j + \psi_Y U) \\
&= \theta_{h,k} x_h + \mathbb{E}_U (\textstyle\sum_{j\in\mathrm{pa}(h)} \theta_{j,k}\, \mathbb{E}_{X_j|U}\, X_j + \psi_Y U) \\
&= \theta_{h,k} x_h + \mathbb{E}_U (\textstyle\sum_{j\in\mathrm{pa}(h)} \theta_{j,k}\, \mathbb{E}_G\, \mathbb{E}_{X_j|G,U}\, X_j + \psi_Y U) \text{ since IV1} \\
&= \theta_{h,k} x_h + \mathbb{E}_U (\textstyle\sum_{j\in\mathrm{pa}(h)} \theta_{j,k}\, \mathbb{E}_G (\sum\limits_{i\in I} G_i \beta^X_{i,j} + \psi_X U) + \psi_Y U) \\
&= \theta_{h,k} x_h + \textstyle\sum_{j\in\mathrm{pa}(h)} \theta_{j,k} (\sum\limits_{i\in I} \mathbb{E}\, G_i \beta^X_{i,j} + \psi_X\, \mathbb{E}\, U) + \psi_Y\, \mathbb{E}\, U.
\end{aligned}$$

■

The next proposition shows that the causal parameter of interest is related to the regression parameters of a regression of Y_k on $\boldsymbol{G}$ and a regression of X_j on $\boldsymbol{G}$.

Proposition S3. The estimand of the causal parameter of interest $\theta_{h,k}$ is the solution of the linear least squares (LLS) regression of $\boldsymbol{\beta}_{Y_k}$ on $\boldsymbol{B}_{X_{\mathrm{fa}(h)}} = \{\boldsymbol{\beta}_{X_j}\}_{j\in\mathrm{pa}(h)}$.

Proof. Define $\overline{G}_i = G_i - \mathbb{E}(G_i)$, $\forall i \in I$, so that $\mathrm{Cov}(Y_k, G_i) = \mathbb{E}(Y_k\overline{G}_i)$. With MCC and model (S1), we have

$$\begin{aligned}
\mathbb{E}(Y_k\overline{G}_i) &= \mathbb{E}_U\,\mathbb{E}_{G_i|U}\,\mathbb{E}_{X_{\mathrm{pa}(h)}|G_i,U}\,\mathbb{E}_{X_h|X_{\mathrm{pa}(h)},G_i,U}\,\mathbb{E}(Y_k\overline{G}_i \mid X_h, \boldsymbol{X}_{\mathrm{pa}(h)}, \overline{G}_i, U)\\
&= \mathbb{E}_U\,\mathbb{E}_{G_i}\,\mathbb{E}_{X_{\mathrm{pa}(h)}|G_i,U}\\
&\quad \mathbb{E}_{X_h|X_{\mathrm{pa}(h)},G_i,U}(\{\theta_{h,k}X_h + \textstyle\sum_{j\in\mathrm{pa}(h)}\theta_{j,k}X_j + \psi_Y U\}\overline{G}_i) \text{ since IV1}\\
&= \theta_{h,k}\,\mathbb{E}_{G_i}\,\mathbb{E}_{X_h|G_i}(X_h\overline{G}_i) + \textstyle\sum_{j\in\mathrm{pa}(h)}\theta_{j,k}\,\mathbb{E}_{G_i}\,\mathbb{E}_{X_j|G_i}(X_j\overline{G}_i) + \psi_Y\,\mathbb{E}_U\,\mathbb{E}_{G_i}(U\overline{G}_i)\\
&= \theta_{h,k}\,\mathbb{E}(X_h\overline{G}_i) + \textstyle\sum_{j\in\mathrm{pa}(h)}\theta_{j,k}\,\mathbb{E}(X_j\overline{G}_i) + \psi_Y\,\mathbb{E}\,U\,\mathbb{E}\,\overline{G}_i \text{ since IV1}\\
&= \theta_{h,k}\,\mathbb{E}(X_h\overline{G}_i) + \textstyle\sum_{j\in\mathrm{pa}(h)}\theta_{j,k}\,\mathbb{E}(X_j\overline{G}_i).
\end{aligned}$$

Dividing both sides by $\mathbb{E}(\overline{G}_i^2)$, we obtain

$$\begin{aligned}
\frac{\mathbb{E}(Y_k\overline{G}_i)}{\mathbb{E}(\overline{G}_i^2)} &= \theta_{h,k}\frac{\mathbb{E}(X_h\overline{G}_i)}{\mathbb{E}(\overline{G}_i^2)} + \sum_{j\in\mathrm{pa}(h)}\theta_{j,k}\frac{\mathbb{E}(X_j\overline{G}_i)}{\mathbb{E}(\overline{G}_i^2)}\\
\beta_{i,k}^Y &= \theta_{h,k}\beta_{i,h}^X + \sum_{j\in\mathrm{pa}(h)}\theta_{j,k}\beta_{i,j}^X
\end{aligned}$$

and in matrix form for all $i \in I$

$$\begin{aligned}
\boldsymbol{\beta}_{Y_k} &= \boldsymbol{\beta}_{X_h}\theta_{h,k} + \textstyle\sum_{j\in\mathrm{pa}(h)}\boldsymbol{\beta}_{X_j}\theta_{j,k}\\
&= \boldsymbol{B}_{X_{\mathrm{fa}(h)}}\boldsymbol{\theta}_{\mathrm{fa}(h),k},
\end{aligned}$$

where $\mathrm{fa}(v) = v \cup \mathrm{pa}(v)$ is the family of v. Assuming that $n > p$, we obtain

$$\theta_{h,k} = [(\boldsymbol{B}_{X_{\mathrm{fa}(h)}}^\top\boldsymbol{B}_{X_{\mathrm{fa}(h)}})^{-1}\boldsymbol{B}_{X_{\mathrm{fa}(h)}}^\top\boldsymbol{\beta}_{Y_k}]_1, \tag{S8}$$

where the subscript indicates the first element of the solution of the LLS regression which is well defined because $\boldsymbol{B}_{X_{\mathrm{fa}(h)}} \neq \boldsymbol{0}$ since IV2. Finally, the estimator of the causal parameter of interest is

$$\widehat{\theta}_{h,k} = [(\widehat{\boldsymbol{B}}_{X_{\mathrm{fa}(h)}}^\top\widehat{\boldsymbol{B}}_{X_{\mathrm{fa}(h)}})^{-1}\widehat{\boldsymbol{B}}_{X_{\mathrm{fa}(h)}}^\top\widehat{\boldsymbol{\beta}}_{Y_k}]_1. \tag{S9}$$

■

Remark S3. The IVW estimator of the main parameter of interest $\theta_{h,k}$ is

$$\begin{aligned}
\widehat{\theta}_{h,k} &= [(\widehat{\boldsymbol{B}}_{X_{\mathrm{fa}(h)}}^\top\overline{\sigma}_Y^{-2}\boldsymbol{V}\widehat{\boldsymbol{B}}_{X_{\mathrm{fa}(h)}})^{-1}\widehat{\boldsymbol{B}}_{X_{\mathrm{fa}(h)}}^\top\overline{\sigma}_Y^{-2}\boldsymbol{V}\widehat{\boldsymbol{\beta}}_{Y_k}]_1\\
&= [(\widehat{\boldsymbol{B}}_{X_{\mathrm{fa}(h)}}^{*\top}\widehat{\boldsymbol{B}}_{X_{\mathrm{fa}(h)}}^{*})^{-1}\widehat{\boldsymbol{B}}_{X_{\mathrm{fa}(h)}}^{*\top}\widehat{\boldsymbol{\beta}}_{Y_k}^{*}]_1,
\end{aligned}$$

where $\overline{\sigma}_Y^2\boldsymbol{V}^{-1} = q^{-1}\sum_{k\in K}\mathbb{V}(\widehat{\boldsymbol{\beta}}_{Y_k})^3$ with $\boldsymbol{V}$ the LD matrix of the population from which the traits are drawn.

Remark S4. Assuming linearity, no interactions and no unobserved confounders, back-door adjustment[4] can be applied to derive the effects of a mediator unconfounded by

other mediators, *i.e.*, the mediation parameter $\gamma^X_{h,j}$ between the hth and jth exposures, $j \neq h \in J$, unconfounded by other exposures and $\gamma^Y_{h,k}$ between the hth and kth outcomes $j \neq h \in J$, unconfounded by other outcomes and/or exposures. In the case of unobserved confounders, as in the proposed MR framework, mediation parameters can be derived, but they do not have a causal interpretation. This is due to the selection of IVs discussed in the main text Section 'Selection of instrumental variables'. When taken on their own, the group of exposures violates condition IV3 (each exposure is not conditionally independent of $\boldsymbol{G}$ given the other exposures and U) and the group of outcomes violates condition IV2 (each outcome is independent of $\boldsymbol{G}$).

Simulation study and real data application

MrDAG parameters setting

In the following, we detail the setting of MrDAG Markov chain Monte Carlo (MCMC) algorithm presented in the main text Appendix that we used in the simulation study and the real data application. In the simulation study, MrDAG algorithm is run for $75,000$ MCMC sweeps, of which $25,000$ as burn-in and results saved every 10 sweeps, resulting in $5,000$ posterior samples for all the unknowns. Since *a priori* we expect one edge for each of the $(q+p)$ nodes, the prior probability of edge inclusion in (12) is

$$\begin{aligned}\pi^{\text{edge}} &= \tfrac{q+p}{(q+p)(q+p-1)/2} \\ &= \tfrac{2}{q+p-1},\end{aligned} \tag{S10}$$

where q is the number of responses, p is the number of exposures and the denominator is the maximum number of edges in a graph with $(q+p)$ nodes. Using (S10) with $q=5$ and $p=15$, the prior probability of edge inclusion is $\pi^{\text{edge}}=0.10$. Finally, the value of the burn-in annealing parameter T is set at 5. No other hyper-parameters need to be defined given the specification of objective priors for model selection based on the fractional Bayes factor, leading to a closed-form expression for the marginal likelihood[5] and the use of objective priors also for the inverse of the covariance matrix[6].

To select a suitable number of MCMC sweeps, and in particular the length of the burn-in, we executed some preliminary runs and checked by visual inspection the trace plots of the marginal likelihood and the model size, *i.e.*, the number of edges selected during the MCMC. No signs of slow convergence or aberrant behaviour of the designed MCMC sampler were identified in a variety of scenarios and parameters settings, suggesting that the number of sweeps during burn-in is sufficient to reach convergence and sample from the equilibrium distribution of the Markov chain.

In the real data analysis, we considerably increase the number of MCMC sweeps to 10^6 of which 10^5 as burn-in and results saved every 100 sweep, resulting in $9,000$ posterior samples for all the unknowns. Assuming *a priori* an edge for each of the $(q+p)$ nodes and using (S10), with $q=7$ and $p=6$, $\pi^{\text{edge}}=0.16$. Finally, the value of the burn-in annealing parameter T is set at 10. As illustrated in Figure S16, the burn-in and the total length of the MCMC far exceed the number of sweeps required to reach convergence to the equilibrium distribution of the Markov chain and explore the graph space faithfully. No signs of slow convergence or aberrant behaviour of the designed MCMC sampler appear. We used the same number of MCMC sweeps, annealing parameter T and π^{edge} to test reverse causation in the real data analysis.

We conclude with the description of MrDAG parameters we used in the bootstrap analysis[7] to check the robustness of the findings in the real data application. We bootstrapped the IVs with replacement $B=100$ times and ran MrDAG algorithm on each bootstrap summary-level statistics. All parameters are left unchanged compared to the original real data analysis, $\pi^{\text{edge}}=0.16$ and $T=10$. However, due to the expensive computational time of each run, we decreased the MCMC sweeps to $75,000$, of which $25,000$ as burn-in without thinning, resulting in $50,000$ posterior samples for each bootstrap sample. The length of the burn-in was suggested by looking at the trace plot of the marginal-log likelihood in a few trail runs. Overall, considering the whole bootstrap analysis, we obtain 7.5×10^6 posterior samples, of which 5×10^6 after burn-in. Finally,

we calculate the bootstrap frequency of edge inclusion as the average of the posterior probability of edge inclusion (PPEI) obtained in each bootstrap sample.

Alternative methods parameters setting

In the simulation study, as an alternative method we would like to compare, we include MR-BMA[8], a Bayesian variable selection approach for multivariable MR, with a prior probability of inclusion set at 0.10. In this way, we match the prior specification of the edge inclusion probability in MrDAG. MR-BMA only considers one outcome at-a-time and is performed on each response separately. We use also MR-BMA in the real data application and we set the prior probability of inclusion set at 0.16 to match that used in MrDAG.

The second alternative method is MR[23]. The algorithm requires the specification of the *a priori* expected number of causal effects for each response and its variance. To match MrDAG sparsity prior, we set `EVgamma = c(2, 2)`, *i.e.*, the prior probability of a causal effects for each response is $2/15 = 0.13$, with $p = 15$ the number of simulated exposures, and where the *a priori* range of causal effects for each response is between 0 and 6. Finally, MR[2] algorithm is run for $15,000$ MCMC sweeps, of which $5,000$ as burn-in and results saved every 10 sweeps, resulting in $1,000$ posterior samples for all the unknowns. In the real data application, we set `EVgamma = c(1, 1)` with the prior probability of a causal effects for each response equal $1/6 = 0.16$, $p = 6$, and the *a priori* range of causal effects for each response between 0 and 4. We also extend the number of sweeps, *i.e.*, we run the algorithm for $150,000$ MCMC sweeps, of which $50,000$ as burn-in and results saved every 100 sweeps, resulting in $1,000$ posterior samples for all the unknowns.

We also include MRPC algorithm[9] based on[10]. It requires the specification of the "sufficient statistic" for the data and the number of "genetic variants". For the former, we calculate the Pearson correlation between the summary-level statistics and provide the number of simulated IVs, $n = 100$. For the latter, we indicate the number of exposures simulated. Since the genetic associations with the exposures and the responses are asymptotically normally distributed, we select `gaussCItest` option to test the conditional independence between the simulated summary-level statistics. We do not correct for multiplicity. Instead, a type I error rate α is used for the conditional independence test. The detected Partial DAG (PDAG) are obtained at different values of $\boldsymbol{\alpha} = \{0.01, 0.05, 0.10, 0.20\}$. Subsequently, they are used to estimate causal effects under intervention by using the function `o.ida()` in the R package *pcalg*[11] which considers "only those edges necessary to compute the possible optimal valid adjustment sets, using these as adjustment sets to estimate the unique possible causal effects" and specifying the option `type = "pdag"`. In the real data application, we set $n = 708$, $p = 6$ and $\alpha = 0.01$ which provides the best results in the simulation study.

Further, we consider Partition DAG algorithm[12] implemented in the R package *PDAG*[13]. It performs structure learning under constraints and causal effects estimation based on Lasso penalisation of the negative log-likelihood. The constraints correspond to a pre-specified partition of the nodes in which domain-specific knowledge provides information regarding their partial ordering. In our setup, the prior knowledge allows us to partition the nodes into exposures and outcomes and the partial ordering information corresponds to the edges' orientation from the exposures to the responses. Since two disjoint groups of nodes are considered, we use the function `partial2()` and specify `m1 = 15` where the

partition between the exposures and the outcomes occurs. Results are recorded for three different values for the Lasso penalisation $\boldsymbol{\lambda} = \{0.5, 0.7, 0.9\}$. Preliminary runs suggest using these values since at zero no penalisation occurs with extremely dense solutions and at 1 the shrinkage is too strong. In the real data application, we set `m1 = 6` and $\lambda = 0.90$ which provides the best results in the simulation study and tried a stability selection approach to set the penalisation parameter as suggested in[12]. The estimated penalisation parameter $\widehat{\lambda} = 0.95$ is very close to the value used in the real data analysis with no appreciable differences regarding the estimated causal effects.

The final alternative method considered in the simulation study is Graph-MRcML[14] which infers a causal network of total effects among multiple traits, then uses a graph deconvolution algorithm[15] to infer the corresponding network of direct effects. In the first step, the MRcML method[16] for invalid IVs screening is employed to infer both causal directions and total effect sizes between two traits while allowing bidirectional relationships. We set `random_start = 10` as recommended. In the second step, a graph deconvolution algorithm is employed on perturbed data sets to achieve more accurate finite-sample inference. We set the number of perturbed data sets at `num_pert = 10` which is much smaller than the recommended one (`num_pert = 200`). However, since the first step on each perturbed data set takes more than 10 minutes due to the large number of traits' pairs to be considered, and overall the algorithm takes more than 2 hours to terminate for each replicate, we limit the number perturbed data sets to 10. In the real data application, we follow step-by-step the instructions regarding the input data format provided by[14] and set `num_pert = 200` as recommended.

Definition of True Positive, False Negative and False Positive

To evaluate the performance of the methods considered and summarize it by a precision-recall curve (PRC), with recall (= sensitivity = TP/(TP + FN)) in the x-axis and precision (= positive predictive value = TP/(TP + FP)) in the y-axis, a definition of TP = True Positive, FN = False Negative and FN = False Positive is required.

However, since the methods considered in the simulation study are quite heterogeneous, there are substantial differences in what they estimate. MrDAG, MRPC and Graph-MRcML algorithms return as output PDAGs. In MrDAG this happens when, at a defined threshold of the PPEI, there are bidirected edges[5], while in the MRPC algorithm, this occurs since undirected edges might still exist after determining all v-structures and applying rules R1-R3 of the PC algorithm as given in Algorithm 2 detailed in[17]. In Graph-MRcML, this depends on the Bonferroni-adjusted significance level. In contrast, Partition DAG provides a fully oriented DAG as output.

For a fair comparison, it is paramount to define TP, FN and FP in a way that they do not favour implicitly any method. We depart from[5,18] who transform a simulated DAG (*true* DAG) into a CPDAG and[12] who modify a CPDAG output by reorienting all undirected edges according to the simulated DAG (as well as to the opposite direction). Instead, we compare the results of the different methods by considering the simulated *true* (partially- and fully-oriented) DAG as described in the main text Appendix.

We motivate this choice as follows:

- Transforming a *true* partially oriented DAG into a CPDAG might violate the partial ordering. If the resulting CPDAG is considered as the background truth and a resulting violation of the partial ordering is present, all methods will experience an increase of the FNs since either they automatically satisfy the constraint of the

causal effects from the exposures to the responses (MR-BMA) or this condition is enforced (MRPC, ParDAG and MrDAG).

- In MRPC and MrDAG, undirected edges might exist only within the exposures and outcomes (recall that an undirected edge $z - v$ is equivalent to $z \to v$ and $v \to z$) while in Graph-MRcML they might be between any pairs of traits. Reorienting undirected edges according to the *true* DAG can be a solution, but it provides a substantial advantage to these methods. Moreover, the divergence of the results, obtained by reorienting the undirected edges based on the true partially oriented DAG or flipping to the opposite direction, might be so large that it is not clear their overall performance. Moreover, in real data applications, flipping the directions is also meaningless since the ground truth is not available.
- Since in two scenarios of the simulation study DAGs are fully oriented and in two scenarios they are partially oriented (but only within the exposures), this should provide alternative advantages to the competing algorithms and, overall, a fair comparison.

We define TP when an algorithm can detect the simulated oriented edge, FN when an algorithm does not declare any oriented edge when instead it should and FP when an algorithm detects an oriented edge when instead it shouldn't. In the case of partially oriented DAGs, TP, FN and FP are defined with respect to the detection of bidirectional edges. In practice, their estimation is simplified by utilising the simulated and estimated non-symmetrical adjacency matrix for each replicate and in each scenario (see Figure 2).

Further results of MrDAG model in the real data application

Here, we present extended results concerning the findings obtained by alternative methods to detect the impact of lifestyle and behavioural traits on mental health phenotypes and internal checks of the results provided by MrDAG algorithm when applied to the same data set.

The risk of detecting spurious shared causal effects is very high when a standard MR method is used separately on each trait as well as when multiple exposures are considered for each outcome[8]. To show that this is a widespread issue which can be solved only if dependency relations are considered in the model, we contrast the results of MrDAG with multivariable single-response MR, implemented in the MR-BMA algorithm which can perform exposure selection[8] but is not designed for multiple outcomes.

Table S3 shows the results MR-BMA algorithm when applied to the same data set. By contrasting Figure 7 in the main text and Table S3 some general comments can be made. First, the signs of the causal effects from the exposures to the outcomes agree. Second, the overestimation of the causal effects by MR-BMA is a general feature since the pleiotropic effects within the outcomes are not considered in MR-BMA which tries to ascribe the whole direct effect to the exposures. See the effect of genetically predicted lifetime smoking index (SM) on BD and the predicted level of physical activity (PA) on major depressive disorder (MDD). Third, as expected from the simulation study, MR-BMA detects many more associations than MrDAG. For instance, genetically predicated sleeping (SP) has been found associated with SCZ and genetically predicted leisure screen time (LST) is associated with AN, ADHD and SCZ. Except for the association of LST with AN, MrDAG does not identify any of these effects. Finally, MR-BMA detects genetically predicated SM on SCZ while MrDAG lists it as a spurious causal effect.

Internal validation is an important step to assess the validity of the results obtained by the MrDAG algorithm. We divide this internal check into sensitivity to hyper-prior specifications and robustness of structure learning.

Regarding the first point, MrDAG is built under an objective Bayes framework which has the advantage of not depending on priors hyper-parameters (see Appendix in the main text). The only parameter that needs to be specified is the prior probability of edge inclusion that controls the level of sparsity. The results presented in the main text Section 'Real data application: The impact of lifestyle and behavioural traits on mental health' were obtained by setting $\pi^{\text{edge}} = 0.16$, *i.e.*, *a priori* we expect one edge for each of the 13 nodes. We repeat the analysis, and specify $\pi^{\text{edge}} = 0.08$ and $\pi^{\text{edge}} = 0.04$. Figure S20 shows that the (Bayesian model-averaged) causal effects as well as the 95% credible intervals (CIs) are not influenced by this choice, with only a handful of cases at $\pi^{\text{edge}} = 0.04$ where the causal effects and the CIs are slightly different.

Although MrDAG explores the space of alternative Essential Graphs (EGs)[19] that best fit the data and produces the posterior estimate of the causal effects averaging over the space of the visited graphical models and thus, taking into account model uncertainty without focusing on a single model, the second question is how robust are the results and how much they should be trusted. We answer these questions by bootstrapping MrDAG repeatedly on the data[7]. In Figures S21 we present the bootstrap frequency of edge inclusion for each permitted combination of exposures and outcomes and the scatterplot of the posterior probability of edge inclusion (PPEI) against the bootstrap frequency of edge inclusion. The results show that there is a satisfactory agreement between a single run of the algorithm and the bootstrap results for the reported causal associations. In four cases we report less alignment. The exposure-outcome pairs SM-SCZ and PA-MDD receive more weight in the bootstrap analysis than in the single run of the algorithm, while the opposite happens for EDU-BD and SM-COG, although, in all cases, the bootstrap frequency of edge inclusion is around 50%.

S2 Tables

Type	Trait	Acronym	Population	Sample size	No. IVs (%)	Reference	Data source
Expsoure	Education (in years)	EDU	European (meta-analysis)	766,345	426† (0.57)	Lee *et. al.*, 2018[20]	`https://thessgac.com/`
	Physical activity	PA	European (meta-analysis + UK Biobank)	608,595	5† (0.01)	Wang *et. al.*, 2022[21]	`https://www.ebi.ac.uk/gwas/studies/GCST90104341`
	Overall sleep duration	SP	European (UK Biobank)	446,118	56† (0.07)	Dashi *et. al.*, 2019[22]	`https://sleep.hugeamp.org/dinspector.html?dataset=GWAS_UKBB_eu`
	Alcohol consumption	ALC	European (UK Biobank)	480,842	63† (0.08)	Evangelou *et. al.*, 2019[23]	`http://ftp.ebi.ac.uk/pub/databases/gwas/summary_statistics/`
	Lifetime smoking index	SM	European (UK Biobank)	462,690	111† (0.15)	Wootton *et. al.*, 2020[24]	`https://data.bris.ac.uk/data/dataset/10i96zb8gm0j81yz0q6ztei23d`
	Leisure screen time	LST	European (meta-analysis + UK Biobank)	526,725	92† (0.12)	Wang *et. al.*, 2022[21]	`https://www.ebi.ac.uk/gwas/studies/GCST90104339`
Outcome	Major depressive disorder	MDD	European (meta-analysis)	170,756 cases and 329,443 controls	45‡ (0.10)	Howard *et. al.*, 2019[25]	`https://pgc.unc.edu/for-researchers/download-results/`
	Anorexia nervosa	AN	European (meta-analysis)	16,992 cases and 55,525 controls	5‡ (0.01)	Watson *et. al.*, 2019[26]	
	Attention deficit hyperactivity disorder	ADHD	European (meta-analysis)	20,183 cases and 35,191 controls	5‡ (0.01)	Demontis *et. al.*, 2019[27]	
	Bipolar disorder	BD	European (meta-analysis)	41,917 cases and 371,549 controls	47‡ (0.10)	Mullins *et. al.*, 2021[28]	
	Autism spectrum disorder	ASD	European (meta-analysis)	18,382 cases and 27,969 controls	1‡ (>0.01)	Grove *et. al.*, 2019[29]	
	Schizophrenia	SCZ	Trans-ethnic (meta-analysis)	76,755 cases 243,649 controls	233‡ (0.50)	Trubetskoy *et. al.*, 2022[30]	
	Cognition	CO	European (meta-analysis)	257,828	154‡ (33%)	Lee *et. al.*, 2018[20]	`https://thessgac.com/`

Table S1. Overview of summary-level statistics used in the real data application to detect the impact of lifestyle and behavioural traits on mental health phenotypes. †Number of non-unique genetic variants selected as IVs for each expsosure. ‡Number of non-unique genetic variants selected as IVs for each phenotypic trait in the reverse causal analysis.

Outcome	Exposure	Effect estimate	Standard error	p-value	FDR
MDD	**EDU**	-0.250	0.030	1.07×10^{-15}	1.12×10^{-14}
	**PA**	-0.280	0.071	0.001	0.004
	SP	-0.048	0.079	0.543	0.671
	ALC	-0.044	0.146	0.764	0.802
	**SM**	0.688	0.080	1.08×10^{-14}	9.06×10^{-14}
	**LST**	0.178	0.028	3.09×10^{-9}	1.62×10^{-8}
AN	**EDU**	0.376	0.075	8.39×10^{-7}	2.52×10^{-6}
	PA	0.348	0.249	0.183	0.319
	SP	-0.155	0.165	0.351	0.460
	ALC	-0.397	0.329	0.232	0.349
	SM	-0.085	0.176	0.632	0.717
	ALC	-0.397	0.329	0.232	0.349
	**LST**	-0.348	0.066	7.00×10^{-7}	2.26×10^{-6}
ADHD	**EDU**	-1.223	0.073	6.16×10^{-50}	1.29×10^{-48}
	PA	-0.566	0.236	0.030	0.063
	SP	-0.051	0.164	0.758	0.802
	ALC	0.021	0.296	0.944	0.967
	**SM**	2.251	0.189	3.03E-23	4.25E-22
	**LST**	0.580	0.068	6.42E-14	4.49E-13
ASD	**EDU**	0.434	0.076	2.35×10^{-8}	9.86×10^{-8}
	PA	-0.076	0.223	0.738	0.802
	SP	-0.084	0.159	0.601	0.701
	ALC	-0.596	0.296	0.047	0.090
	SM	0.218	0.172	0.207	0.347
	LST	0.068	0.073	0.349	0.460
BD	**EDU**	0.307	0.066	4.50×10^{-6}	1.26×10^{-5}
	PA	0.005	0.166	0.978	0.978
	**SP**	0.375	0.147	0.013	0.030
	ALC	0.281	0.243	0.252	0.365
	**SM**	0.899	0.139	1.56×10^{-9}	9.36×10^{-9}
	LST	-0.156	0.068	0.024	0.053
SCZ	EDU	0.089	0.074	0.231	0.349
	PA	-0.203	0.239	0.410	0.521
	**SP**	0.499	0.156	0.002	0.005
	ALC	0.167	0.300	0.578	0.694
	**SM**	1.036	0.172	1.37×10^{-8}	6.41×10^{-8}
	LST	-0.167	0.077	0.032	0.064
COG	**EDU**	0.773	0.020	6.93×10^{-146}	2.91×10^{-144}
	PA	0.163	0.099	0.123	0.224
	SP	-0.061	0.058	0.294	0.412
	ALC	0.136	0.113	0.233	0.349
	**SM**	-0.318	0.061	6.32×10^{-7}	2.21×10^{-6}
	**LST**	-0.166	0.029	7.25×10^{-8}	2.77×10^{-7}

Table S2. Results of standard MR[31] with one exposure one outcome at-a-time regarding how lifestyle and behavioural exposures impact mental health outcomes. Results include the causal effect estimates, the standard error and the corresponding p-value. We adjust for multiple testing using Benjamini-Hochberg False Discovery Rate (FDR)[32] across all exposures and outcomes. Exposures selected at 5% FDR are highlighted in bold.

Outcome	Exposure	MACE	mPPI	Empirical p-value	FDR
MDD	EDU	-0.005	0.136	0.693	0.693
	**PA**	-0.168	0.971	0.001	0.002
	SP	-0.023	0.291	0.251	0.301
	ALC	-0.078	0.482	0.208	0.301
	**SM**	0.608	1.000	1.00×10^{-5}	6.00×10^{-5}
	LST	0.023	0.362	0.054	0.107
AN	EDU	0.049	0.286	0.115	0.173
	PA	0.085	0.388	0.087	0.173
	**SP**	-0.374	0.943	0.003	0.009
	ALC	-0.047	0.249	0.945	0.945
	SM	0.094	0.347	0.228	0.273
	**LST**	-0.397	1.000	1.00×10^{-5}	6.00×10^{-5}
ADHD	**EDU**	-0.750	1.000	1.00×10^{-5}	3.00×10^{-5}
	PA	0.029	0.203	0.377	0.565
	SP	-0.010	0.135	0.965	0.965
	ALC	-0.037	0.236	0.965	0.965
	**SM**	1.464	1.000	1.00×10^{-5}	3.00×10^{-5}
	**LST**	0.273	0.983	1.30×10^{-4}	2.60×10^{-4}
ASD	**EDU**	0.723	1.000	1.00×10^{-5}	6.00×10^{-5}
	**PA**	-0.251	0.769	0.009	0.014
	SP	0.002	0.114	1.000	1.000
	**ALC**	-0.608	0.909	0.010	0.014
	**SM**	0.480	0.908	0.004	0.011
	**LST**	0.073	0.423	0.037	0.044
BD	**EDU**	0.396	0.994	9.00×10^{-5}	2.70×10^{-4}
	PA	0.107	0.476	0.053	0.079
	SP	0.099	0.462	0.092	0.110
	ALC	0.057	0.282	0.864	0.864
	**SM**	1.115	1.000	1.00×10^{-5}	6.00×10^{-5}
	**LST**	-0.133	0.712	0.008	0.016
SCZ	EDU	0.041	0.266	0.134	0.201
	PA	0.005	0.121	0.974	0.974
	**SP**	0.423	0.986	0.001	0.001
	ALC	0.063	0.282	0.863	0.974
	**SM**	1.233	1.000	1.00×10^{-5}	6.00×10^{-5}
	**LST**	-0.329	0.998	3.00×10^{-5}	9.00×10^{-5}
COG	**EDU**	0.820	1.000	1.00×10^{-5}	6.00×10^{-5}
	**PA**	-0.062	0.724	0.013	0.031
	SP	-0.018	0.324	0.198	0.297
	ALC	0.016	0.270	0.898	0.898
	**SM**	0.094	0.785	0.016	0.031
	LST	0.002	0.106	0.751	0.898

Table S3. Results of Mendelian Randomisation with Bayesian model averaging (MR-BMA) algorithm regarding how lifestyle and behavioural exposures impact mental health outcomes. MR-BMA[8] is run on each outcome separately. Results include the model-averaged causal effect estimate (MACE), the marginal posterior probability of inclusion (mPPI) and the corresponding empirical p-value[33]. For each outcome, we adjust for multiple testing using Benjamini-Hochberg False Discovery Rate (FDR) across all exposures. Exposures selected at (5/no of outcomes) = 0.714% FDR are highlighted in bold.

S3 Figures

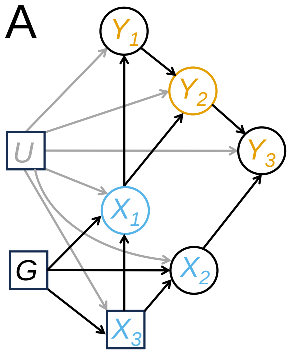

Figure S1. Sufficient adjustment sets required by the back-door criterion to guarantee that the association between X_1 and Y_2 is purely causative in the example depicted in Figure 2C. (**A**) $Y_2(x_1) \perp\!\!\!\perp X_1 \mid C$, $\forall x_1$, where $C = \{X_3, \boldsymbol{G}, U\}$ is the sufficient adjustment set[34]. By conditioning on $\{X_3, \boldsymbol{G}, U\}$, depicted as squares, there are no back-door paths from X_1 to Y_2. (**B**) $Y_2(x_1) \perp\!\!\!\perp X_1 \mid C$, $\forall x_1$, where $C = \{U\}$. The second sufficient adjustment set is obtained by using the function `adjustmentSets()` in the R package *dagitty* which implements the adjustment criterion presented in[35], an extension of Pearl's back-door criterion[4]. In (A) and (B), both sufficient adjustment sets include the unobserved confounder U.

Simulation study

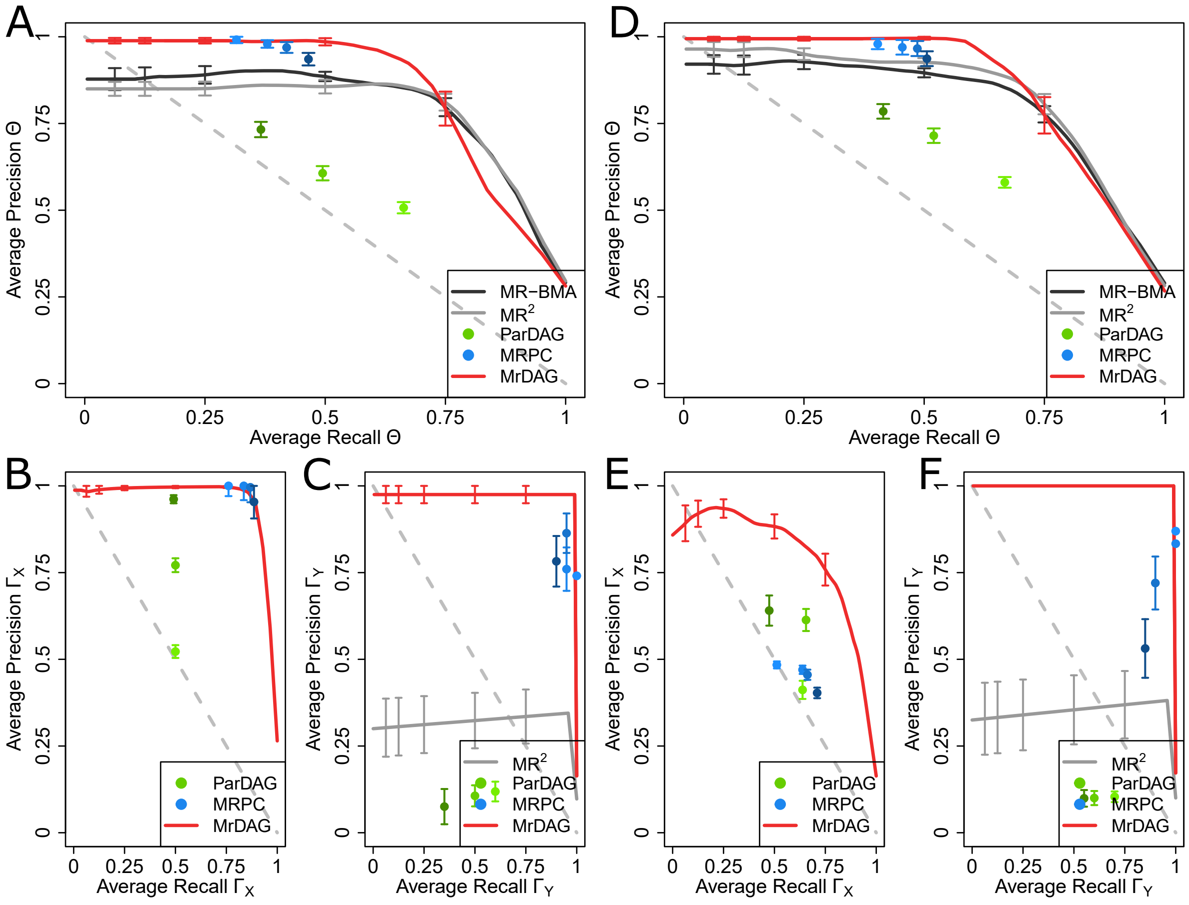

Figure S2. Precision-Recall Curves (PRCs) for all methods considered in the simulated scenarios UndG$_X$-Med$_Y$ and DAG$_X$-Med$_Y$ show recall (= sensitivity = TP/(TP+FN)) in the x-axis and precision (= positive predictive value = TP/(TP+FP)) in the y-axis with TP = True Positive, FN = False Negative and FN = False Positive averaged over 25 replicates in each scenario and for all methods considered. In scenario UndG$_X$-Med$_Y$ (**A**-**C**), the strength of the correlation between consecutive $\boldsymbol{X}$ is set at $r_X = 0.6$, and then it decreases exponentially for non-consecutive exposures, and the level of complete mediation in $\boldsymbol{Y}$ is set at $m_Y = 1$, while in scenario DAG$_X$-Med$_Y$ (**D**-**F**), the average level of the mediation parameters within $\boldsymbol{X}$ and the level of complete mediation in $\boldsymbol{Y}$ is set at $r_X = 0.6$ and $m_Y = 1$, respectively. For details, see in the main text Appendix. In both scenarios, the results are presented separately for the simulated dependency structures from the exposures to the outcomes (A and D), within the exposures (B and E) and the outcomes (C and D), respectively. Vertical bars in each PRCs, at specific recall levels $0.0625, 0.125, 0.25, 0.50$ and 0.75, indicate standard error. Vertical bars in each PRC, at specific recall levels $0.0625, 0.125, 0.25, 0.50$ and 0.75, indicate standard error. For the MRPC algorithm, the type I error rate for the conditional independence test is set at $\boldsymbol{\alpha} = \{0.01, 0.05, 0.10, 0.20\}$ (from light- to dark-blue dots) and for the ParDAG algorithm we specify three different values for the Lasso penalisation $\boldsymbol{\lambda} = \{0.5, 0.7, 0.9\}$ (from light- to dark-green dots). See Section 'Simulation study and real data application' for details.

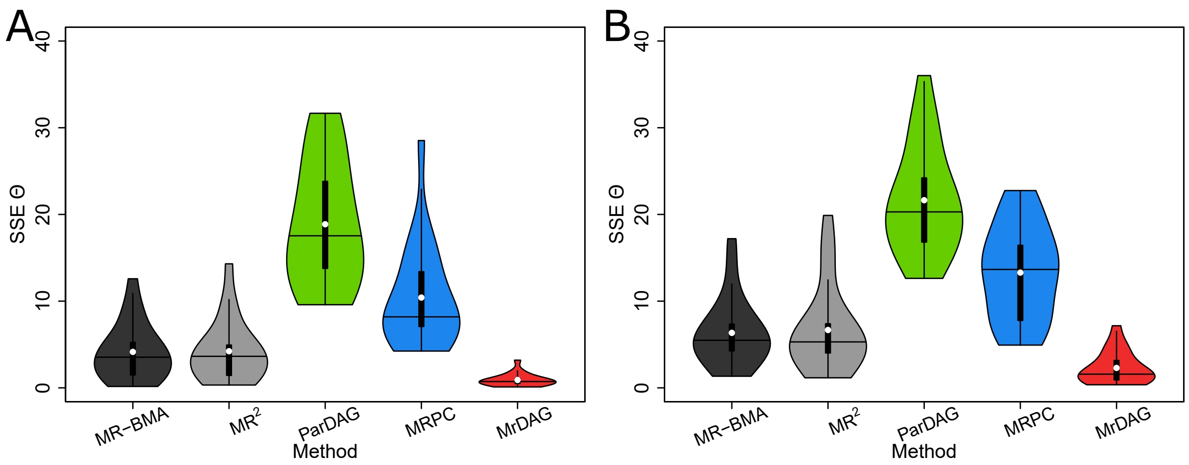

Figure S3. Violin plots of the Sum of Squares Error (SSE) of the causal effects Θ between the exposures and the outcomes for all methods considered in the simulated scenarios UndG$_X$-Med$_Y$ and DAG$_X$-Med$_Y$ across 25 replicates in each scenario. (**A**) In scenario UndG$_X$-Med$_Y$, the strength of correlation between consecutive $\boldsymbol{X}$ is set at $r_X = 0.6$, and then it decreases exponentially for non-consecutive exposures, and the level of complete mediation in $\boldsymbol{Y}$ is set at $m_Y = 1$. (**B**) In scenario DAG$_X$-Med$_Y$, the average level of the mediation parameters within $\boldsymbol{X}$ and the level of complete mediation in $\boldsymbol{Y}$ is set at $r_X = 0.6$ and $m_Y = 1$, respectively. For details, see in the main text Appendix. In each violin plot, the vertical black thick line displays the interquartile range, the black horizontal line denotes the median and the white dot the mean. For MRPC and ParDAG algorithms, we only show the results obtained at type I error rate for the conditional independence test $\alpha = 0.01$ and Lasso penalisation $\lambda = 0.9$, respectively. These values provide the best results for the two algorithms as shown in Figure 5 and Figure S2.

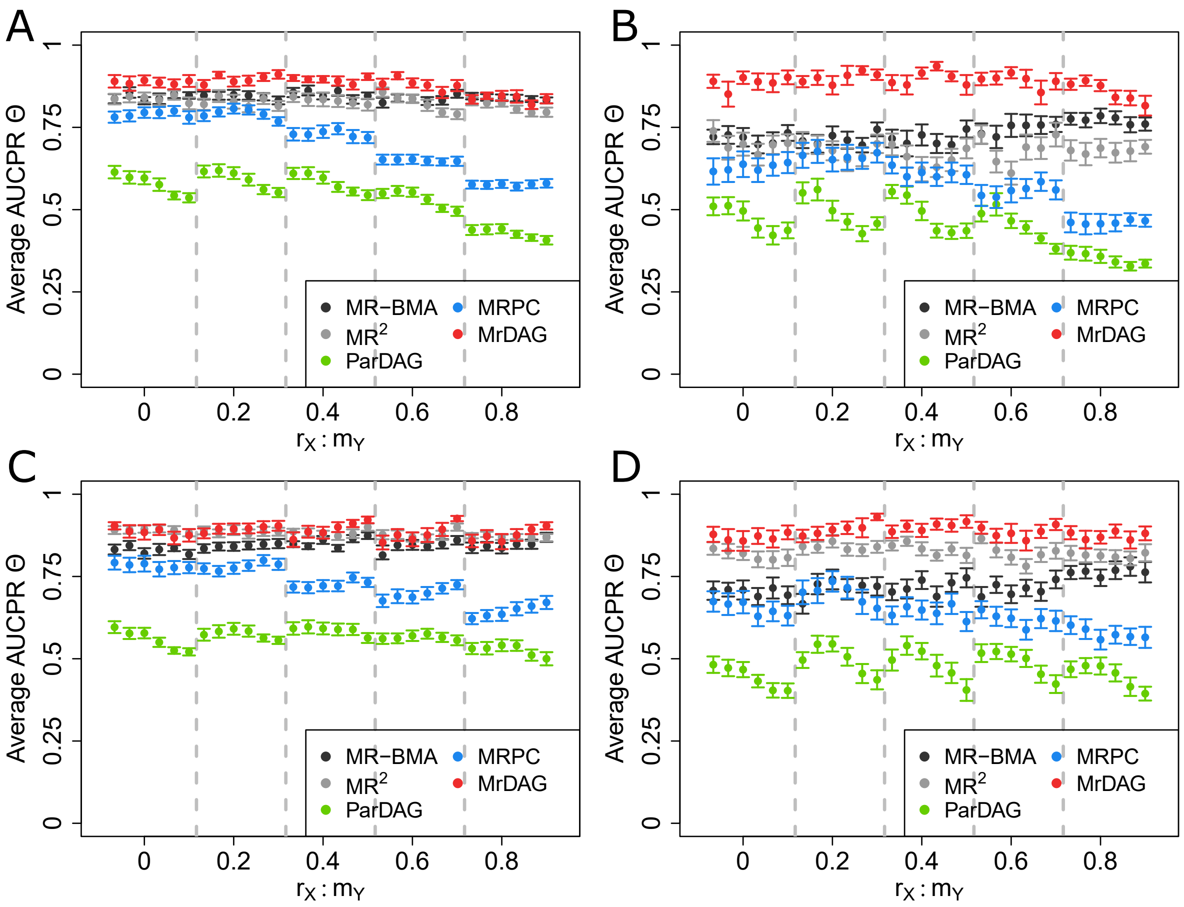

Figure S4. Area Under the Curve of Precision-Recall (AUCPR) of the causal effects Θ between the exposures and the outcomes for all methods considered and simulated scenarios averaged across 25 replicates for different value of the parameters $\boldsymbol{r}_X = \{0, 0.2, 0.4, 0.6, 0.8\}$ which controls the strength of correlation between consecutive $\boldsymbol{X}$, and then it decreases exponentially for non-consecutive exposures, in scenarios (**A**) UndG$_X$-Med$_Y$ and (**B**) UndG$_X$-DAG$_Y$ or the average level of the mediation parameters within $\boldsymbol{X}$ in scenarios (**C**) DAG$_X$-Med$_Y$ and (**D**) DAG$_X$-DAG$_Y$, and $\boldsymbol{m}_Y = \{0.25, 0.50, 0.75, 1, 1.5, 2\}$ which regulates the level of complete mediation in scenarios (**A**) UndG$_X$-Med$_Y$ and (**C**) DAG$_X$-Med$_Y$ or the average level of the mediation parameters within $\boldsymbol{Y}$ in scenarios (**B**) UndG$_X$-DAG$_Y$ and (**D**) DAG$_X$-DAG$_Y$, respectively. For details, see in the main text Appendix. At each value of r_X in the x-axis and delimited by vertical-dotted lines, the AUCPRs are plotted against all values of m_Y. The average value of the AUCPR and its standard error (vertical bar for each AUCPR) has been calculated using the functions `prediction()` and `performance()` in the R package *ROCR*[36,37]. For MRPC and ParDAG algorithms, we only show the results obtained at type I error rate for the conditional independence test $\alpha = 0.01$ and Lasso penalisation $\lambda = 0.9$, respectively. These values provide the best results for the two algorithms as shown in Figure 5 and Figure S2.

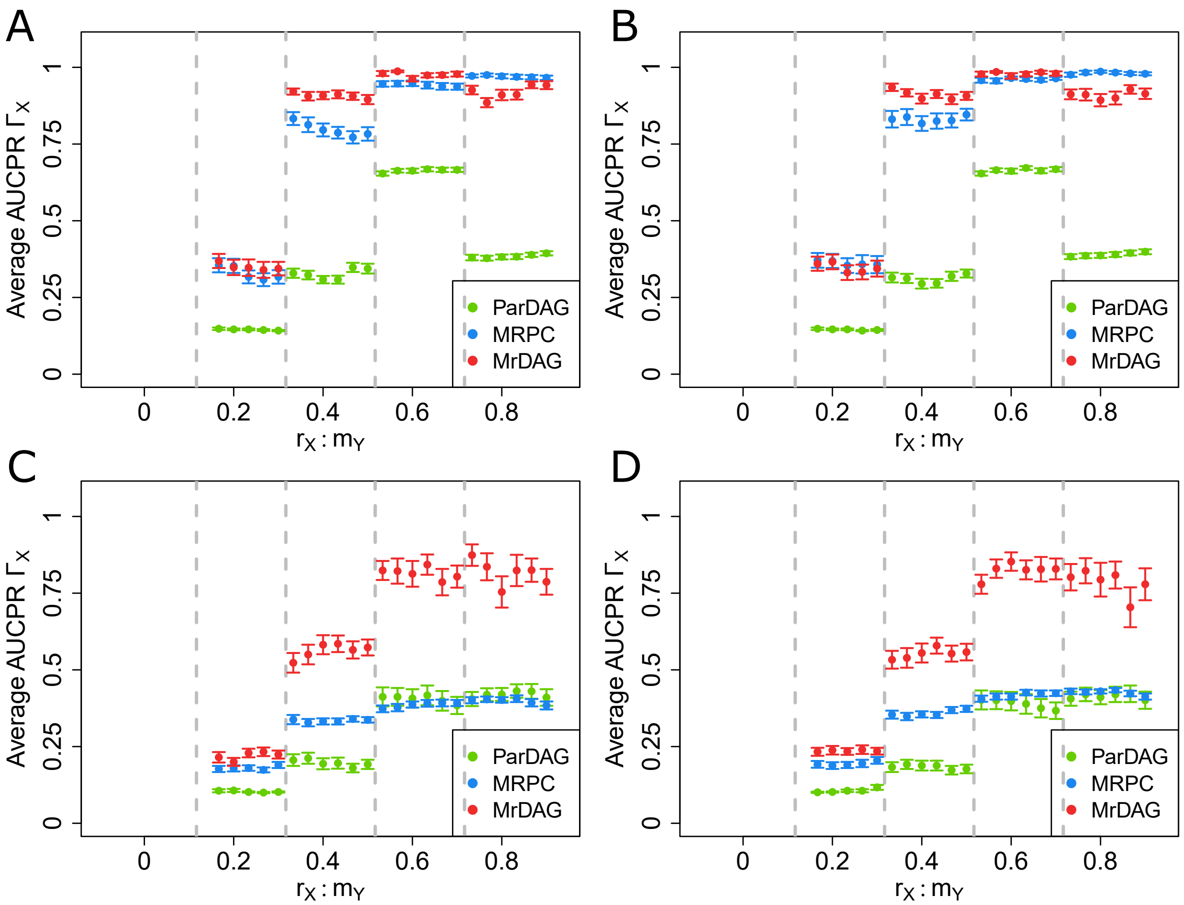

Figure S5. Area Under the Curve of Precision-Recall (AUCPR) of the detection of the dependency structure within the exposures for all methods considered and simulated scenarios averaged across 25 replicates for different value of the parameters $\boldsymbol{r}_X = \{0, 0.2, 0.4, 0.6, 0.8\}$ which controls the strength of correlation between consecutive $\boldsymbol{X}$, and then it decreases exponentially for non-consecutive exposures, in scenarios (**A**) UndG$_X$-Med$_Y$ and (**B**) UndG$_X$-DAG$_Y$ or the average level of the mediation parameters within $\boldsymbol{X}$ in scenarios (**C**) DAG$_X$-Med$_Y$ and (**D**) DAG$_X$-DAG$_Y$, and $\boldsymbol{m}_Y = \{0.25, 0.50, 0.75, 1, 1.5, 2\}$ which regulates the average level of complete mediation in scenarios (**A**) UndG$_X$-Med$_Y$ and (**C**) DAG$_X$-Med$_Y$ or the average level of the mediation parameters within $\boldsymbol{Y}$ in scenarios (**B**) UndG$_X$-DAG$_Y$ and (**D**) DAG$_X$-DAG$_Y$, respectively. For details, see in the main text Appendix. At each value of r_X in the x-axis and delimited by vertical-dotted lines, the AUCPRs are plotted against all values of m_Y. The average value of the AUCPR and its standard error (vertical bar for each AUCPR) has been calculated using the functions `prediction()` and `performance()` in the R package *ROCR*[36,37]. For MRPC and ParDAG algorithms, we only show the results obtained at type I error rate for the conditional independence test $\alpha = 0.01$ and Lasso penalisation $\lambda = 0.9$, respectively. These values provide the best results for the two algorithms as shown in Figure 5 and Figure S2.

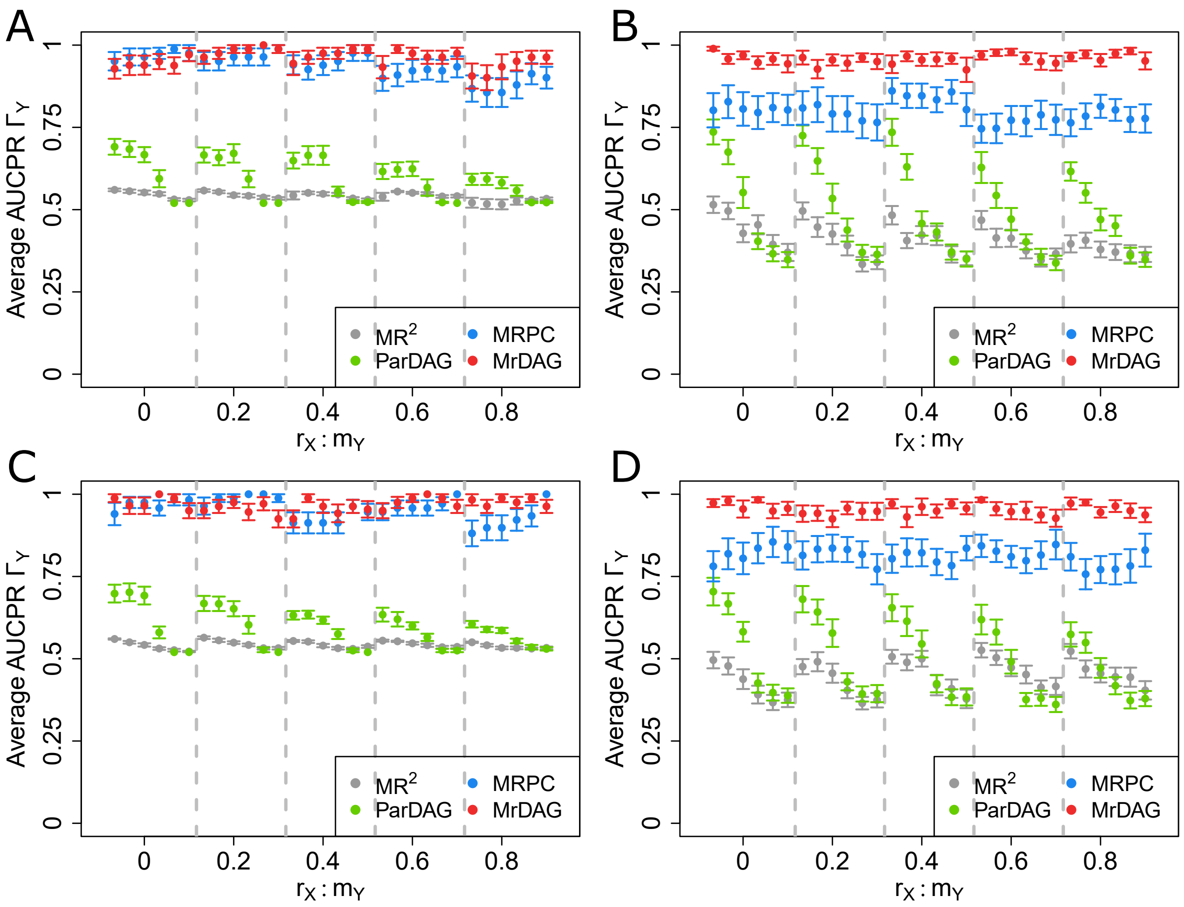

Figure S6. Area Under the Curve of Precision-Recall (AUCPR) of the detection of the dependency structure within the outcomes for all methods considered and simulated scenarios averaged across 25 replicates for different value of the parameters $\boldsymbol{r}_X = \{0, 0.2, 0.4, 0.6, 0.8\}$ which controls the strength of correlation between consecutive $\boldsymbol{X}$, and then it decreases exponentially for non-consecutive exposures, in scenarios (**A**) UndG$_X$-Med$_Y$ and (**B**) UndG$_X$-DAG$_Y$ or the average level of the mediation parameters within $\boldsymbol{X}$ in scenarios (**C**) DAG$_X$-Med$_Y$ and (**D**) DAG$_X$-DAG$_Y$, and $\boldsymbol{m}_Y = \{0.25, 0.50, 0.75, 1, 1.5, 2\}$ which regulates the level of complete mediation in scenarios (**A**) UndG$_X$-Med$_Y$ and (**C**) DAG$_X$-Med$_Y$ or the average level of the mediation parameters within $\boldsymbol{Y}$ in scenarios (**B**) UndG$_X$-DAG$_Y$ and (**D**) DAG$_X$-DAG$_Y$, respectively. For details, see in the main text Appendix. At each value of r_X in the x-axis and delimited by vertical-dotted lines, the AUCPRs are plotted against all values of m_Y. The average value of the AUCPR and its standard error (vertical bar for each AUCPR) has been calculated using the functions `prediction()` and `performance()` in the R package *ROCR*[36,37]. For MRPC and ParDAG algorithms, we only show the results obtained at type I error rate for the conditional independence test $\alpha = 0.01$ and Lasso penalisation $\lambda = 0.9$, respectively. These values provide the best results for the two algorithms as shown in Figure 5 and Figure S2.

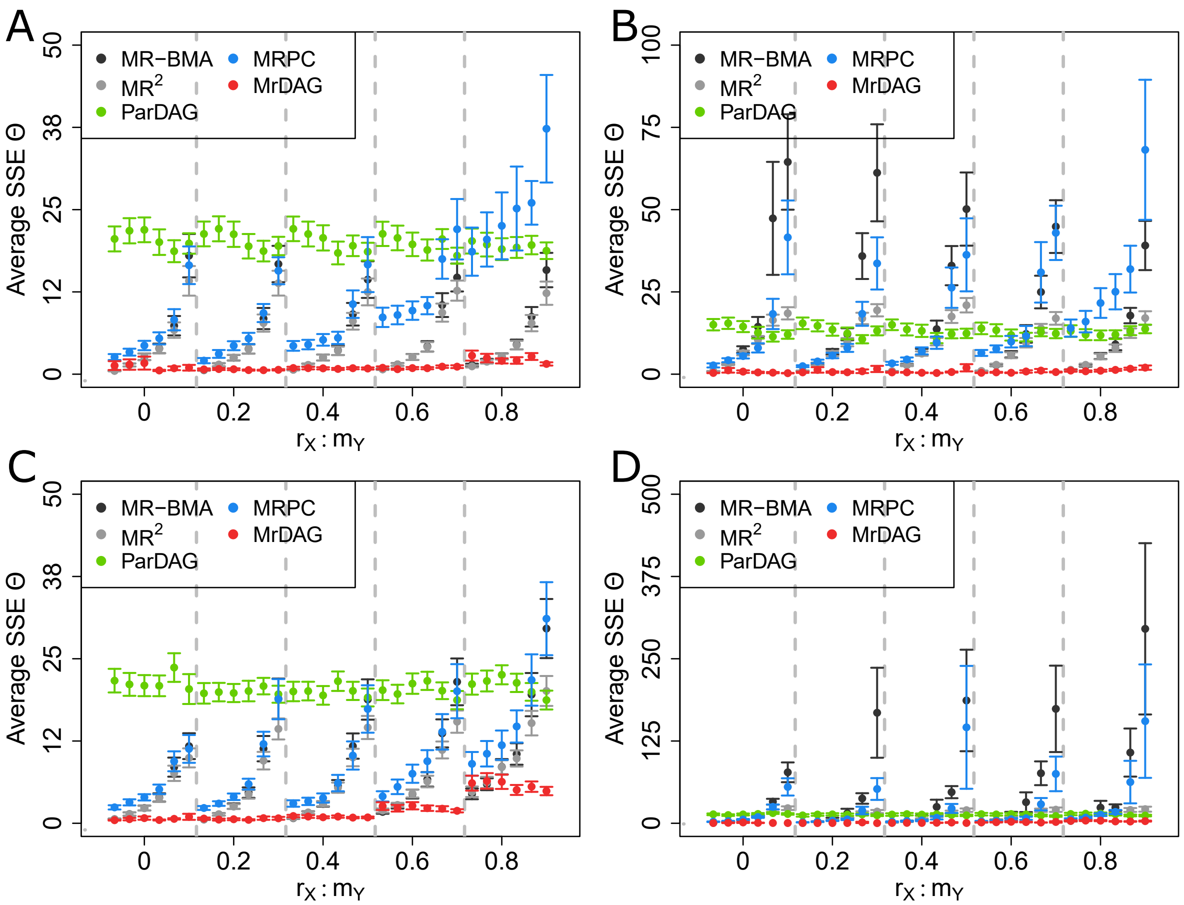

Figure S7. Sum of Squared Error (SSE) of the causal effects Θ between the exposures and the outcomes for all methods considered and simulated scenarios averaged across 25 replicates for different value of the parameters $\boldsymbol{r}_X = \{0, 0.2, 0.4, 0.6, 0.8\}$ which controls the strength of correlation between consecutive exposures, and then it decreases exponentially for non-consecutive exposures, in scenarios (**A**) UndG$_X$-Med$_Y$ and (**B**) UndG$_X$-DAG$_Y$ or the average level of the mediation parameters within $\boldsymbol{X}$ in scenarios (**C**) DAG$_X$-Med$_Y$ and (**D**) DAG$_X$-DAG$_Y$, and $\boldsymbol{m}_Y = \{0.25, 0.50, 0.75, 1, 1.5, 2\}$ which regulates the level of complete mediation in scenarios (**A**) UndG$_X$-Med$_Y$ and (**C**) DAG$_X$-Med$_Y$ or the average level of the mediation parameters within $\boldsymbol{Y}$ in scenarios (**B**) UndG$_X$-DAG$_Y$ and (**D**) DAG$_X$-DAG$_Y$, respectively. For details, see in the main text Appendix. At each value of r_X in the x-axis and delimited by vertical-dotted lines, the SSEs are plotted against all values of m_Y. Vertical bars for each SSE indicate standard error. For MRPC and ParDAG algorithms, we only show the results obtained at type I error rate for the conditional independence test $\alpha = 0.01$ and Lasso penalisation $\lambda = 0.9$, respectively. These values provide the best results for the two algorithms as shown in Figure 5 and Figure S2.

Simulation study: Robustness to noisy genetic association estimates

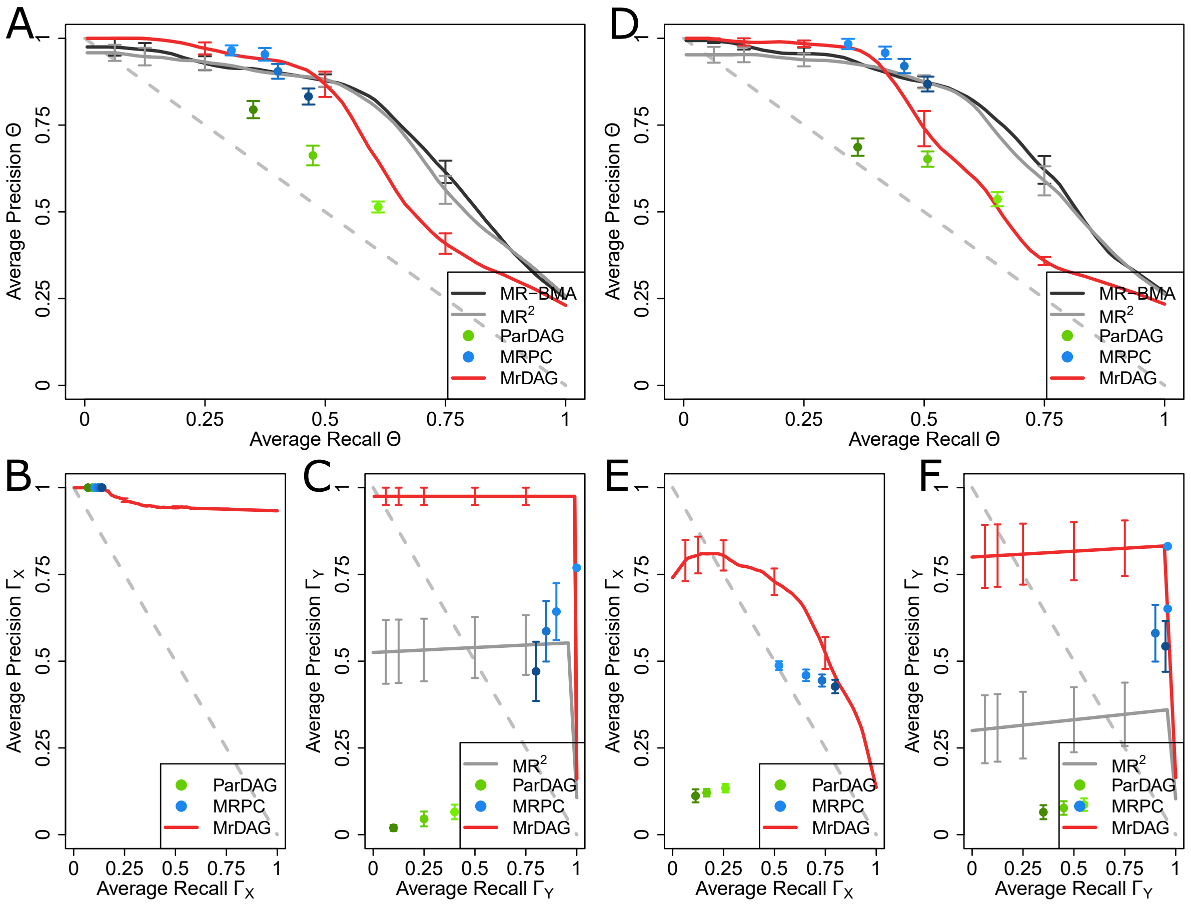

Figure S8. Precision-Recall Curves (PRCs) for all methods considered in the simulated scenarios UndG$_X$-Med$_Y$ and DAG$_X$-Med$_Y$ when the number of simulated individuals is decreased to N = 20,000 to mimic noisy genetic association estimates, equally split into $N_Y = N_X = 10,000$, show recall (= sensitivity = TP/(TP + FN)) in the x-axis and precision (= positive predictive value = TP/(TP + FP)) in the y-axis with TP = True Positive, FN = False Negative and FN = False Positive averaged over 25 replicates in each scenario and for all methods considered. In scenario UndG$_X$-Med$_Y$ (**A-C**), the strength of the correlation between consecutive $\boldsymbol{X}$ is set at $r_X = 0.6$, and then it decreases exponentially for non-consecutive exposures, and the level of complete mediation in $\boldsymbol{Y}$ is set at $m_Y = 1$, while in scenario DAG$_X$-Med$_Y$ (**D-F**), the average level of the mediation parameters within $\boldsymbol{X}$ and the level of complete mediation in $\boldsymbol{Y}$ is set at $r_X = 0.6$ and $m_Y = 1$, respectively. For details, see in the main text Appendix. In both scenarios, the results are presented separately for the simulated dependency structures from the exposures to the outcomes (A and D), within the exposures (B and E) and the outcomes (C and D), respectively. Vertical bars in each PRCs, at specific recall levels $0.0625, 0.125, 0.25, 0.50$ and 0.75, indicate standard error. Vertical bars in each PRC, at specific recall levels $0.0625, 0.125, 0.25, 0.50$ and 0.75, indicate standard error. For the MRPC algorithm, the type I error rate for the conditional independence test is set at $\boldsymbol{\alpha} = \{0.01, 0.05, 0.10, 0.20\}$ (from light- to dark-blue dots) and for the ParDAG algorithm we specify three different values for the Lasso penalisation $\boldsymbol{\lambda} = \{0.5, 0.7, 0.9\}$ (from light- to dark-green dots). See Section 'Simulation study and real data application' for details.

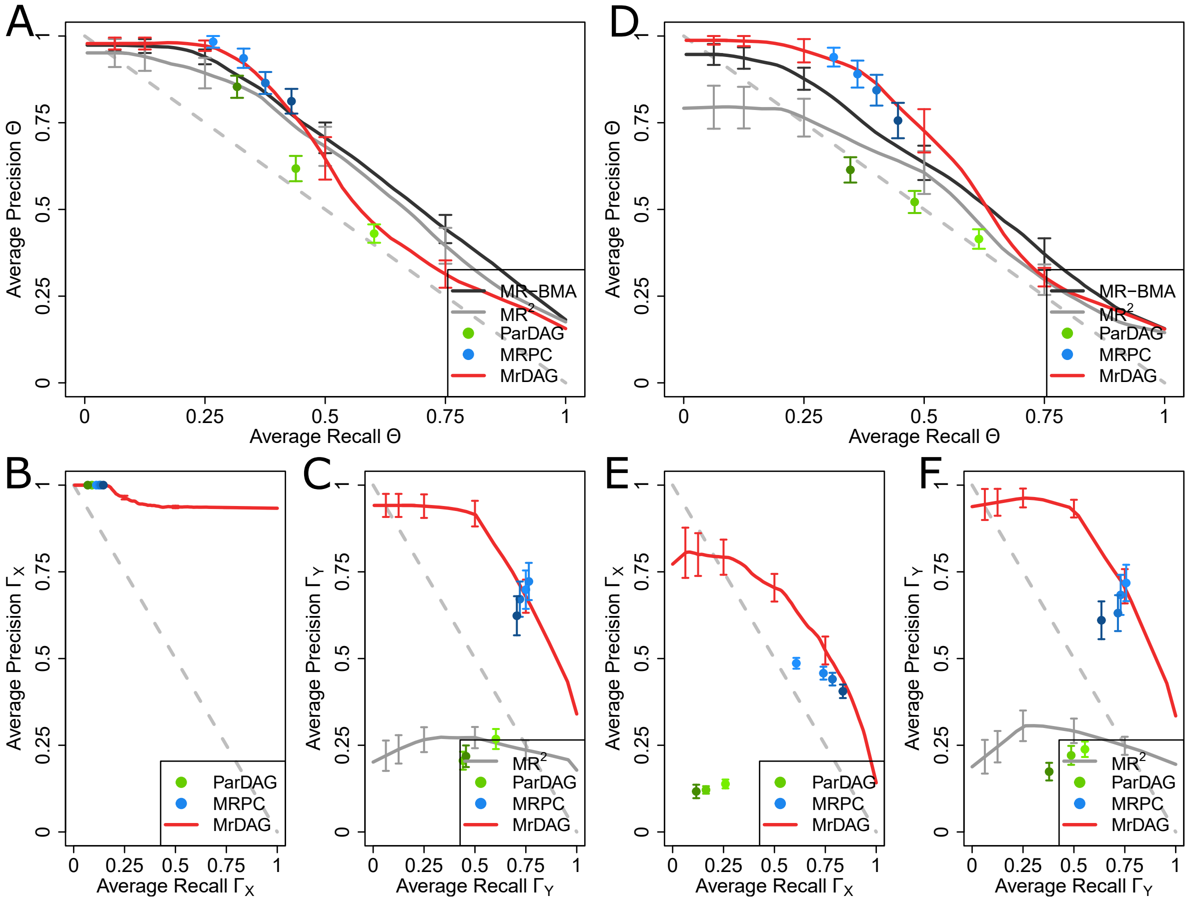

Figure S9. Precision-recall curves (PRCs) for all methods considered in the simulated scenarios UndG$_X$-DAG$_Y$ and DAG$_X$-DAG$_Y$ when the number of simulated individuals is decreased to $\boldsymbol{N} = \mathbf{20{,}000}$ to mimic noisy genetic association estimates, equally split into $N_Y = N_X = 10,000$, show recall (= sensitivity = TP/(TP + FN)) in the x-axis and precision (= positive predictive value = TP/(TP + FP)) in the y-axis with TP = True Positive, FN = False Negative and FN = False Positive averaged over 25 replicates in each scenario. In scenario UndG$_X$-DAG$_Y$ (**A**-**C**), the strength of correlation between consecutive $\boldsymbol{X}$ is set at $r_X = 0.6$, and then it decreases exponentially for non-consecutive exposures, and the average level of the mediation parameters within $\boldsymbol{Y}$ is set at $m_Y = 1$, while in scenario DAG$_X$-DAG$_Y$ (**D**-**F**), the average level of the mediation parameters within $\boldsymbol{X}$ and $\boldsymbol{Y}$ is set at $r_X = 0.6$ and $m_Y = 1$, respectively. For details, see in the main text Appendix. In both scenarios, the results are presented separately for the simulated dependency structures from the exposures to the outcomes (A and D), within the exposures (B and E) and the outcomes (C and D), respectively. Vertical bars in each PRCs, at specific recall levels $0.0625, 0.125, 0.25, 0.50$ and 0.75, indicate standard error. Vertical bars in each PRC, at specific recall levels $0.0625, 0.125, 0.25, 0.50$ and 0.75, indicate standard error. For the MRPC algorithm, the type I error rate for the conditional independence test is set at $\boldsymbol{\alpha} = \{0.01, 0.05, 0.10, 0.20\}$ (from light- to dark-blue dots) and for the ParDAG algorithm we specify three different values for the Lasso penalisation $\boldsymbol{\lambda} = \{0.5, 0.7, 0.9\}$ (from light- to dark-green dots). See Section 'Simulation study and real data application' for details.

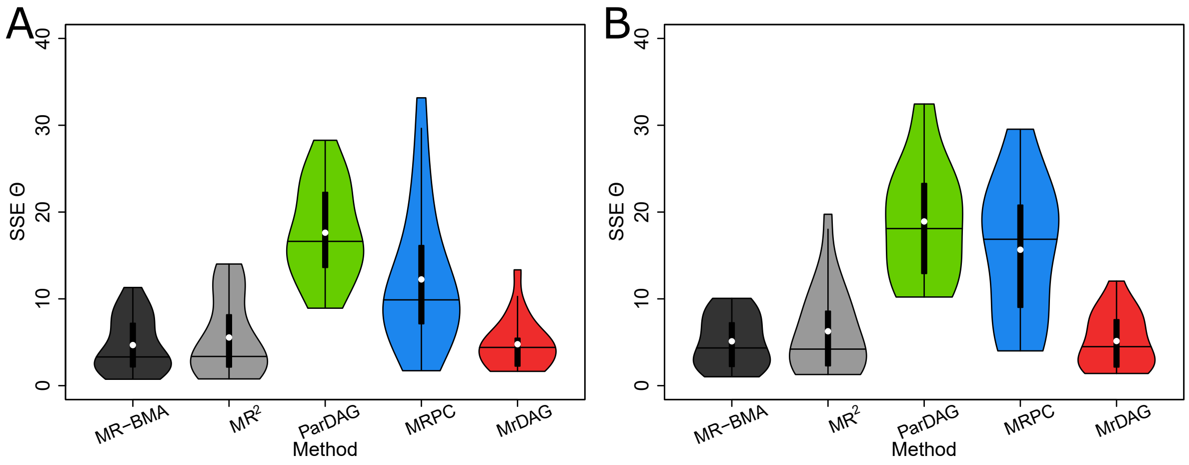

Figure S10. Violin plots of the Sum of Squares Error (SSE) of the causal effects Θ between the exposures and the outcomes for all methods considered in the simulated scenarios UndG$_X$-Med$_Y$ and UndG$_X$-Med$_Y$ and DAG$_X$-Med$_Y$ when the number of simulated individuals is decreased to $N = 20,000$ to mimic noisy genetic association estimates, equally split into $N_Y = N_X = 10,000$, across 25 replicates in each scenario. (**A**) In scenario UndG$_X$-Med$_Y$, the strength of correlation between consecutive $\boldsymbol{X}$ is set at $r_X = 0.6$, and then it decreases exponentially for non-consecutive exposures, and the level of complete mediation in $\boldsymbol{Y}$ is set at $m_Y = 1$. (**B**) In scenario DAG$_X$-Med$_Y$, the average level of the mediation parameters within $\boldsymbol{X}$ and the level of complete mediation in $\boldsymbol{Y}$ is set at $r_X = 0.6$ and $m_Y = 1$, respectively. For details, see in the main text Appendix. In each violin plot, the vertical black thick line displays the interquartile range, the black horizontal line denotes the median and the white dot the mean. For MRPC and ParDAG algorithms, the results are obtained at type I error rate for the conditional independence test $\alpha = 0.01$ and Lasso penalisation $\lambda = 0.9$, respectively.

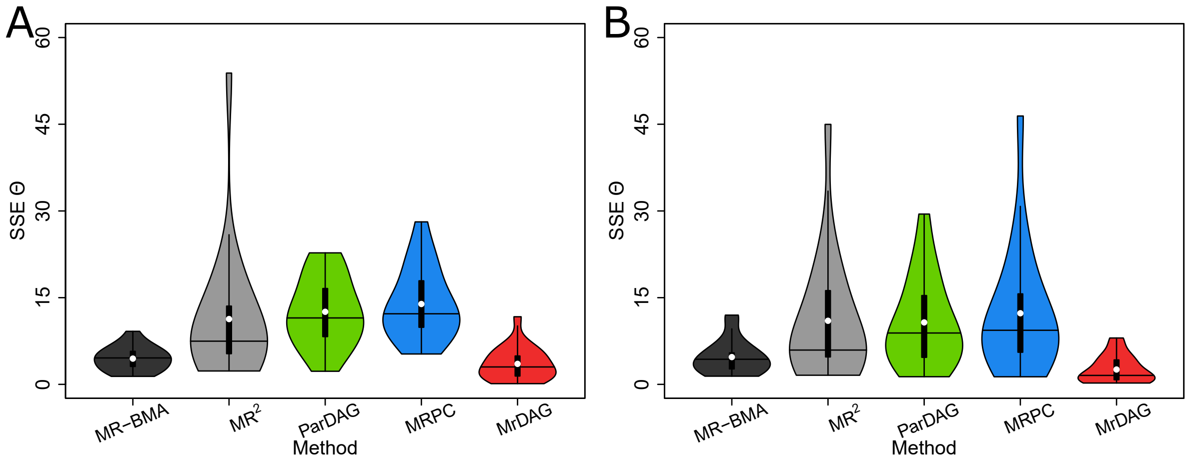

Figure S11. Violin plots of the Sum of Squares Error (SSE) of the causal effects Θ between the exposures and the outcomes for all methods considered in the simulated scenarios UndG$_X$-DAG$_Y$ and DAG$_X$-DAG$_Y$ when the number of simulated individuals is decreased to $N = 20,000$, equally split into $N_Y = N_X = 10,000$, across 25 replicates in each scenario. (**A**) In scenario UndG$_X$-DAG$_Y$, the strength of correlation between consecutive $\boldsymbol{X}$ is set at $r_X = 0.6$, and then it decreases exponentially for non-consecutive exposures, and the average level of the mediation parameters within $\boldsymbol{Y}$ is set at $m_Y = 1$. (**B**) In scenario DAG$_X$-DAG$_Y$, the average level of the mediation parameters within $\boldsymbol{X}$ and $\boldsymbol{Y}$ is set at $r_X = 0.6$ and $m_Y = 1$, respectively. For details, see Appendix. In each violin plot, the vertical black thick line displays the interquartile range, the black horizontal line denotes the median and the white dot the mean. For MRPC and ParDAG algorithms, the results are obtained at type I error rate for the conditional independence test $\alpha = 0.01$ and Lasso penalisation $\lambda = 0.9$, respectively.

Simulation study: Robustness to misspecification of the exposure/outcome groups' definition

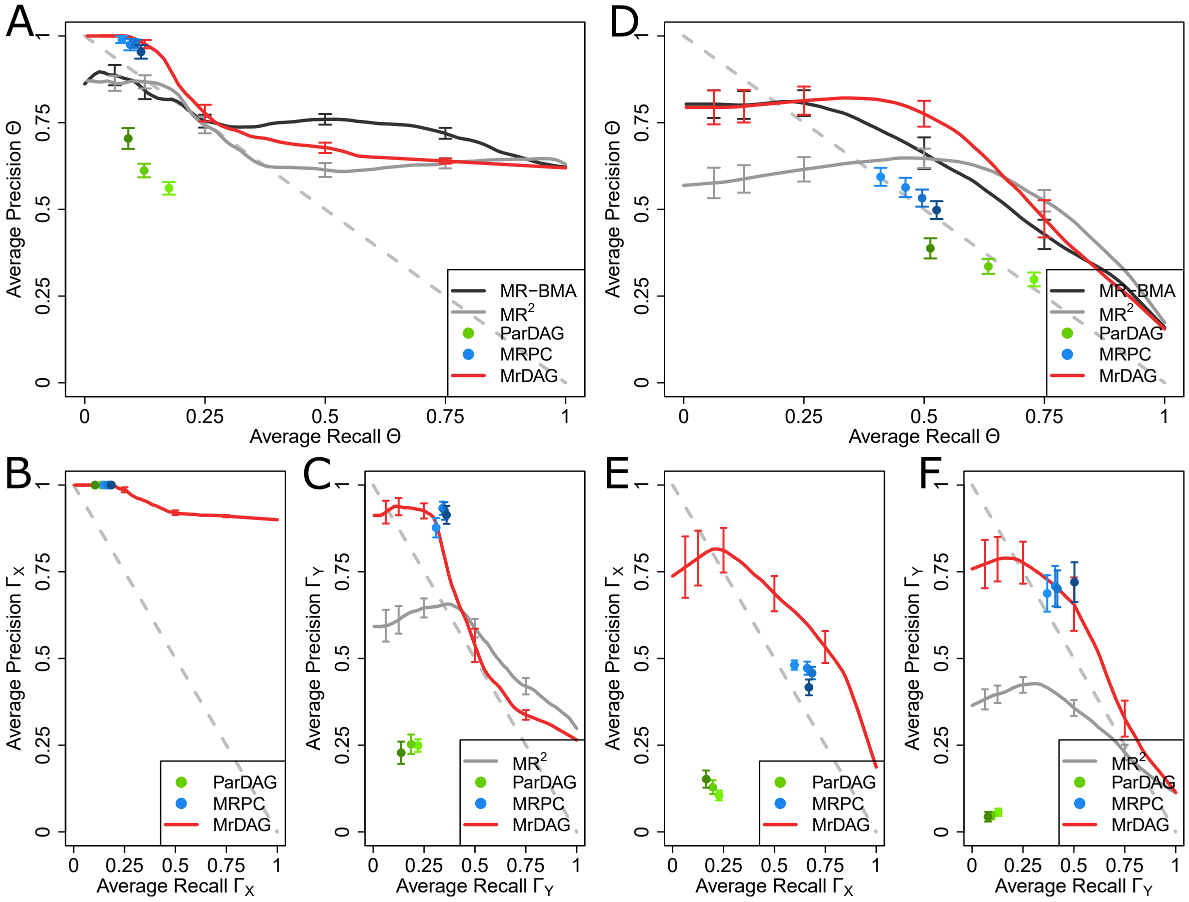

Figure S12. Precision-Recall Curves (PRCs) for all methods considered in the simulated scenarios UndG$_X$-Med$_Y$ and DAG$_X$-Med$_Y$ under misspecification of the exposure-outcome groups' definition, show recall (= sensitivity = TP/(TP + FN)) in the x-axis and precision (= positive predictive value = TP/(TP + FP)) in the y-axis with TP = True Positive, FN = False Negative and FN = False Positive averaged over 25 replicates in each scenario and for all methods considered. In scenario UndG$_X$-Med$_Y$ (**A**-**C**), the strength of the correlation between consecutive $\boldsymbol{X}$ is set at $r_X = 0.6$, and then it decreases exponentially for non-consecutive exposures, and the level of complete mediation in $\boldsymbol{Y}$ is set at $m_Y = 1$, while in scenario DAG$_X$-Med$_Y$ (**D**-**F**), the average level of the mediation parameters within $\boldsymbol{X}$ and the level of complete mediation in $\boldsymbol{Y}$ is set at $r_X = 0.6$ and $m_Y = 1$, respectively. For details, see in the main text Appendix. In both scenarios, the results are presented separately for the simulated dependency structures from the exposures to the outcomes (A and D), within the exposures (B and E) and the outcomes (C and D), respectively. Vertical bars in each PRCs, at specific recall levels $0.0625, 0.125, 0.25, 0.50$ and 0.75, indicate standard error. Vertical bars in each PRC, at specific recall levels $0.0625, 0.125, 0.25, 0.50$ and 0.75, indicate standard error. For the MRPC algorithm, the type I error rate for the conditional independence test is set at $\boldsymbol{\alpha} = \{0.01, 0.05, 0.10, 0.20\}$ (from light- to dark-blue dots) and for the ParDAG algorithm we specify three different values for the Lasso penalisation $\boldsymbol{\lambda} = \{0.5, 0.7, 0.9\}$ (from light- to dark-green dots). See Section 'Simulation study and real data application' for details.

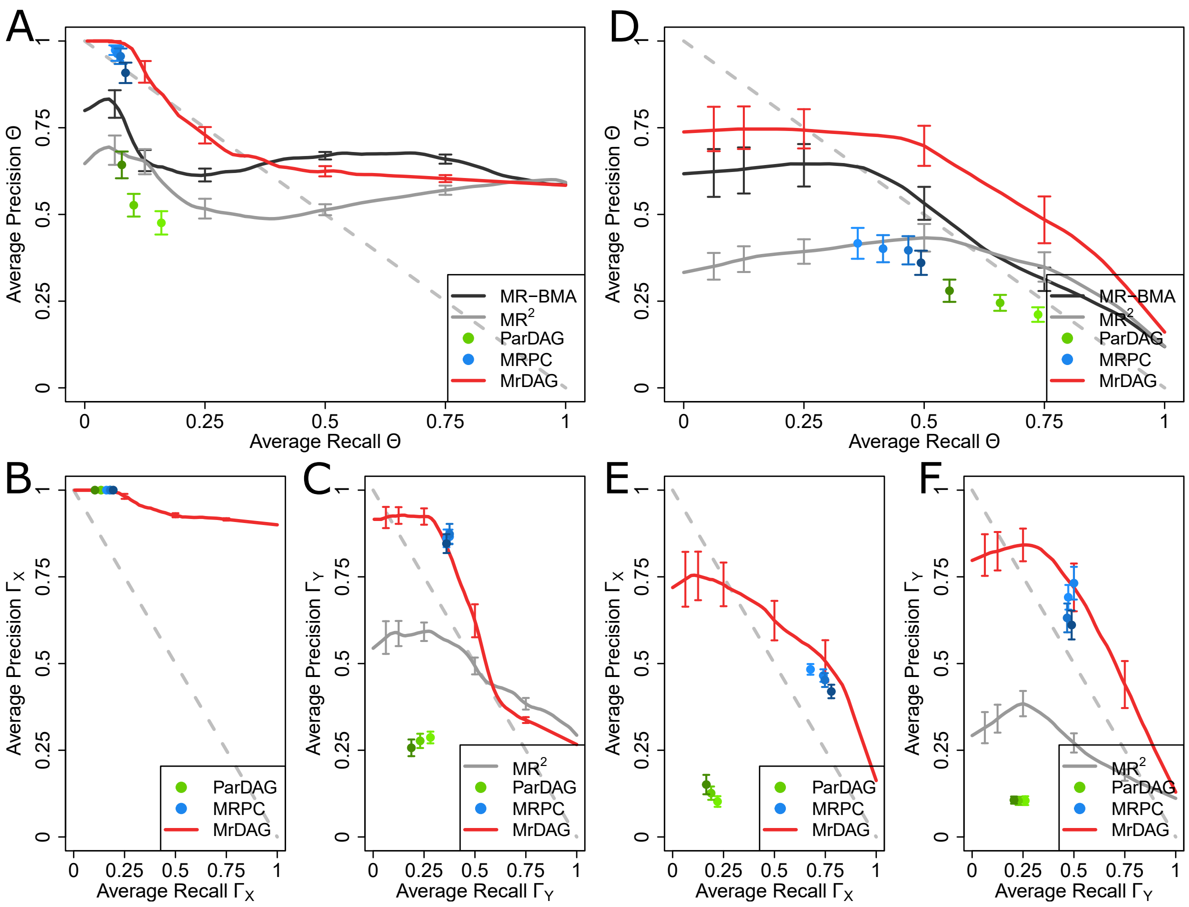

Figure S13. Precision-recall curves (PRCs) for all methods considered in the simulated scenarios UndG$_X$-DAG$_Y$ and DAG$_X$-DAG$_Y$ under misspecification of the exposure-outcome groups' definition, show recall (= sensitivity = TP/(TP + FN)) in the x-axis and precision (= positive predictive value = TP/(TP + FP)) in the y-axis with TP = True Positive, FN = False Negative and FN = False Positive averaged over 25 replicates in each scenario. In scenario UndG$_X$-DAG$_Y$ (**A**-**C**), the strength of correlation between consecutive $\boldsymbol{X}$ is set at $r_X = 0.6$, and then it decreases exponentially for non-consecutive exposures, and the average level of the mediation parameters within $\boldsymbol{Y}$ is set at $m_Y = 1$, while in scenario DAG$_X$-DAG$_Y$ (**D**-**F**), the average level of the mediation parameters within $\boldsymbol{X}$ and $\boldsymbol{Y}$ is set at $r_X = 0.6$ and $m_Y = 1$, respectively. For details, see in the main text Appendix. In both scenarios, the results are presented separately for the simulated dependency structures from the exposures to the outcomes (A and D), within the exposures (B and E) and the outcomes (C and D), respectively. Vertical bars in each PRCs, at specific recall levels $0.0625, 0.125, 0.25, 0.50$ and 0.75, indicate standard error. Vertical bars in each PRC, at specific recall levels $0.0625, 0.125, 0.25, 0.50$ and 0.75, indicate standard error. For the MRPC algorithm, the type I error rate for the conditional independence test is set at $\boldsymbol{\alpha} = \{0.01, 0.05, 0.10, 0.20\}$ (from light- to dark-blue dots) and for the ParDAG algorithm we specify three different values for the Lasso penalisation $\boldsymbol{\lambda} = \{0.5, 0.7, 0.9\}$ (from light- to dark-green dots). See Section 'Simulation study and real data application' for details.

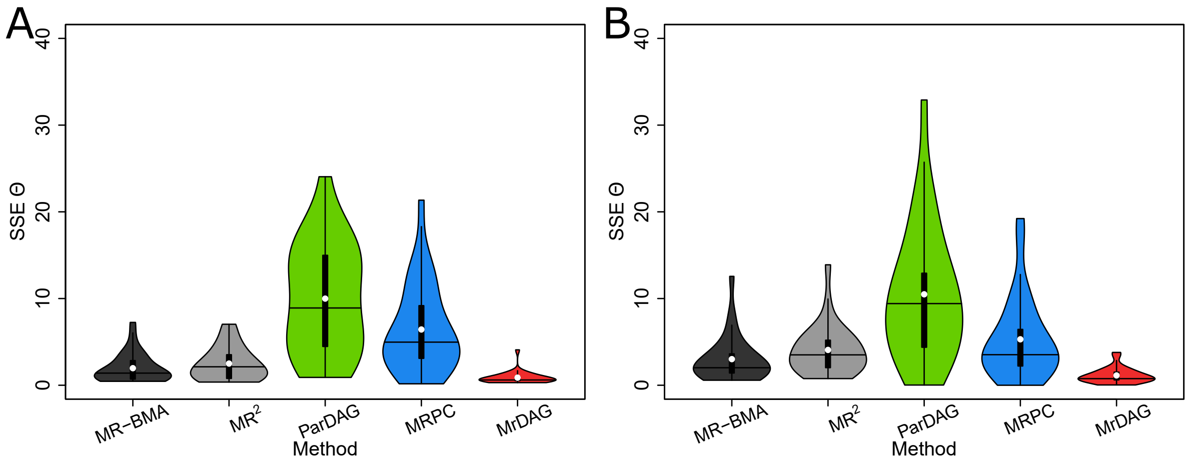

Figure S14. Violin plots of the Sum of Squares Error (SSE) of the causal effects Θ between the exposures and the outcomes for all methods considered in the simulated scenarios UndG$_X$-Med$_Y$ and DAG$_X$-Med$_Y$ under misspecification of the exposure-outcome groups' definition across 25 replicates in each scenario. (**A**) In scenario UndG$_X$-Med$_Y$, the strength of correlation between consecutive $\boldsymbol{X}$ is set at $r_X = 0.6$, and then it decreases exponentially for non-consecutive exposures, and the level of complete mediation in $\boldsymbol{Y}$ is set at $m_Y = 1$. (**B**) In scenario DAG$_X$-Med$_Y$, the average level of the mediation parameters within $\boldsymbol{X}$ and the level of complete mediation in $\boldsymbol{Y}$ is set at $r_X = 0.6$ and $m_Y = 1$, respectively. For details, see in the main text Appendix. In each violin plot, the vertical black thick line displays the interquartile range, the black horizontal line denotes the median and the white dot the mean. For MRPC and ParDAG algorithms, we only show the results obtained at type I error rate for the conditional independence test $\alpha = 0.01$ and Lasso penalisation $\lambda = 0.9$, respectively.

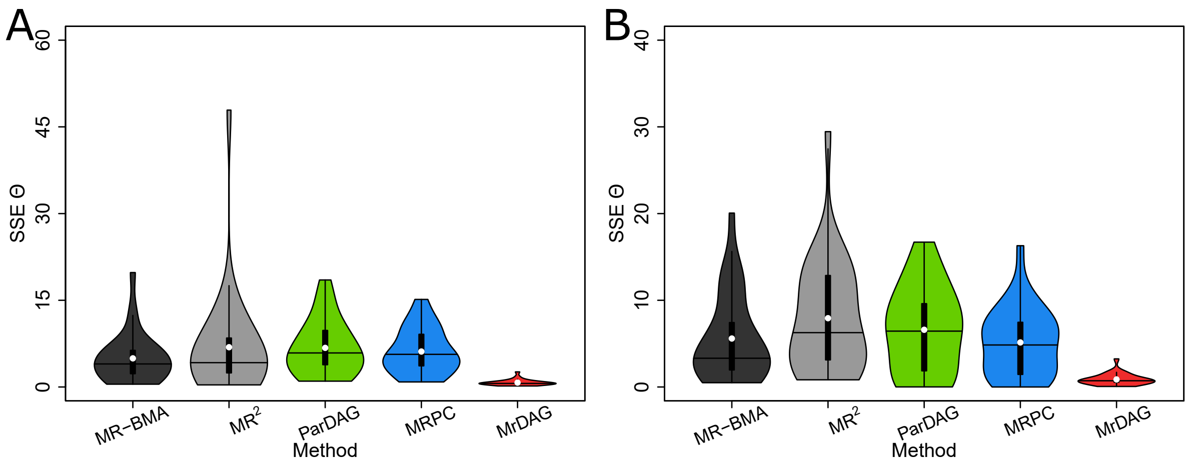

Figure S15. Violin plots of the Sum of Squares Error (SSE) of the causal effects Θ between the exposures and the outcomes for all methods considered in the simulated scenarios UndG$_X$-DAG$_Y$ and DAG$_X$-DAG$_Y$ under misspecification of the exposure-outcome groups' definition across 25 replicates in each scenario. (**A**) In scenario UndG$_X$-DAG$_Y$, the strength of correlation between consecutive $\boldsymbol{X}$ is set at $r_X = 0.6$, and then it decreases exponentially for non-consecutive exposures, and the average level of the mediation parameters within $\boldsymbol{Y}$ is set at $m_Y = 1$. (**B**) In scenario DAG$_X$-DAG$_Y$, the average level of the mediation parameters within $\boldsymbol{X}$ and $\boldsymbol{Y}$ is set at $r_X = 0.6$ and $m_Y = 1$, respectively. For details, see Appendix. In each violin plot, the vertical black thick line displays the interquartile range, the black horizontal line denotes the median and the white dot the mean. For MRPC and ParDAG algorithms, we only show the results obtained at type I error rate for the conditional independence test $\alpha = 0.01$ and Lasso penalisation $\lambda = 0.9$, respectively.

Real data application

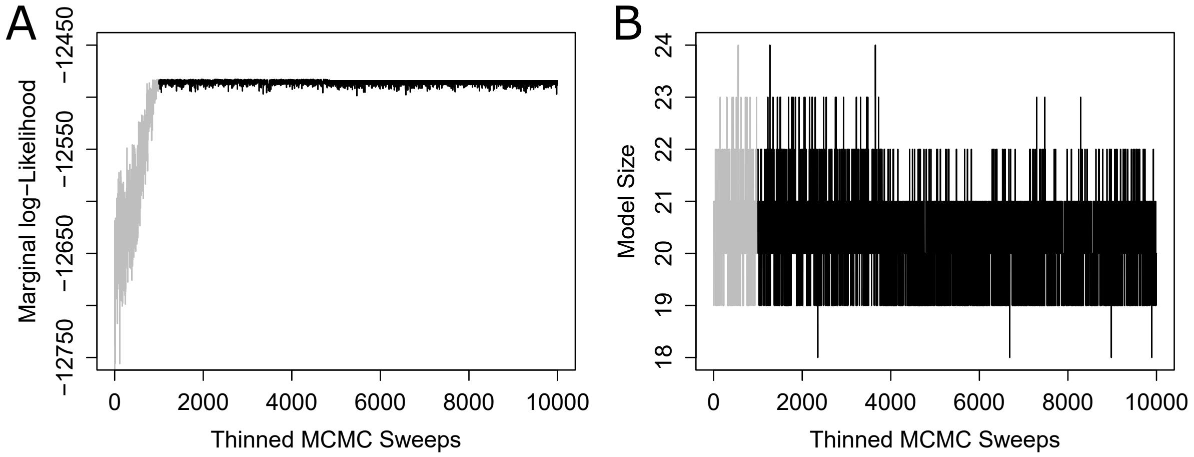

Figure S16. Convergence diagnostics of MrDAG algorithm to assess how lifestyle and behavioural exposures impact mental health outcomes. Grey colour denotes burn-in up to 10^5 MCMC sweeps, while black colour shows values after the burn-in. Quantities reported in the y-axis are recorded at every 100 MCMC sweeps, resulting in $10,000$ thinned recorded values (x-axis). (**A**) Trace plot of the log-marginal likelihood $m_{\mathcal{G}}(\text{data})$ during 10^6 MCMC sweeps. In the burn-in, the effect of the annealing parameter produces a larger excursion of the log-marginal likelihood, allowing the algorithm to escape from local maxima and thus favouring an efficient exploration of regions of high posterior mass. (**B**) Trace plot of the posterior model size, *i.e.*, the number of directed edges selected during the MCMC. The range of the model size is between 18 and 24 with an average of ≈ 21 selected edges. After burn-in, the posterior model size is much larger than the prior model size which is set at one edge for each of the $(q+p)$ nodes ($q = 7$ for the outcomes and $p = 6$ for the exposures) resulting in 13 nodes.

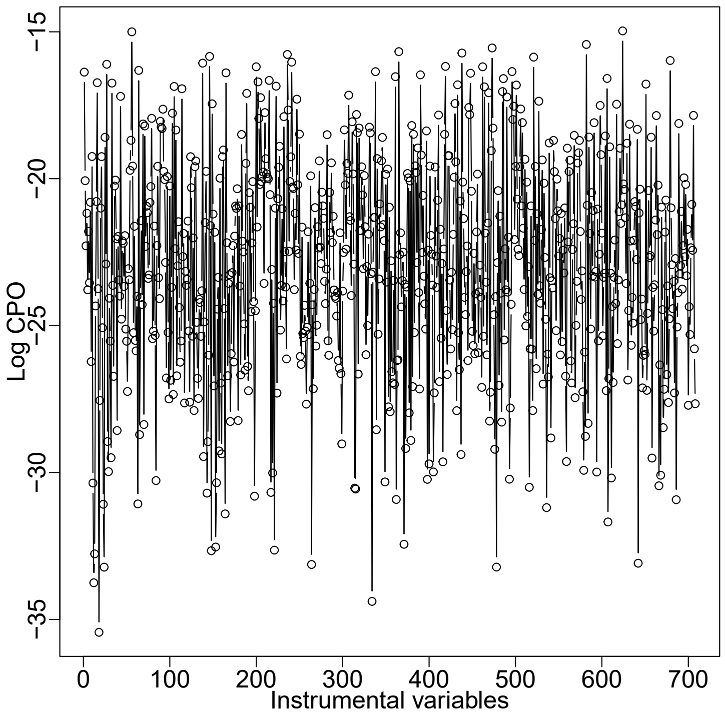

Figure S17. Invalid instrumental variables (IVs) detection by using MrDAG output in the real data application regarding how lifestyle and behavioural exposures impact mental health outcomes. Log-conditional predictive ordinate (CPO) (y-axis) is plotted against 708 IVs (x-axis). No IVs with scaled CPOs below 0.01 to be regarded as outliers are detected[38]. No IVs show log-inverse-CPOs larger than 40 (possible outliers) and higher than 70 (extreme values)[39].

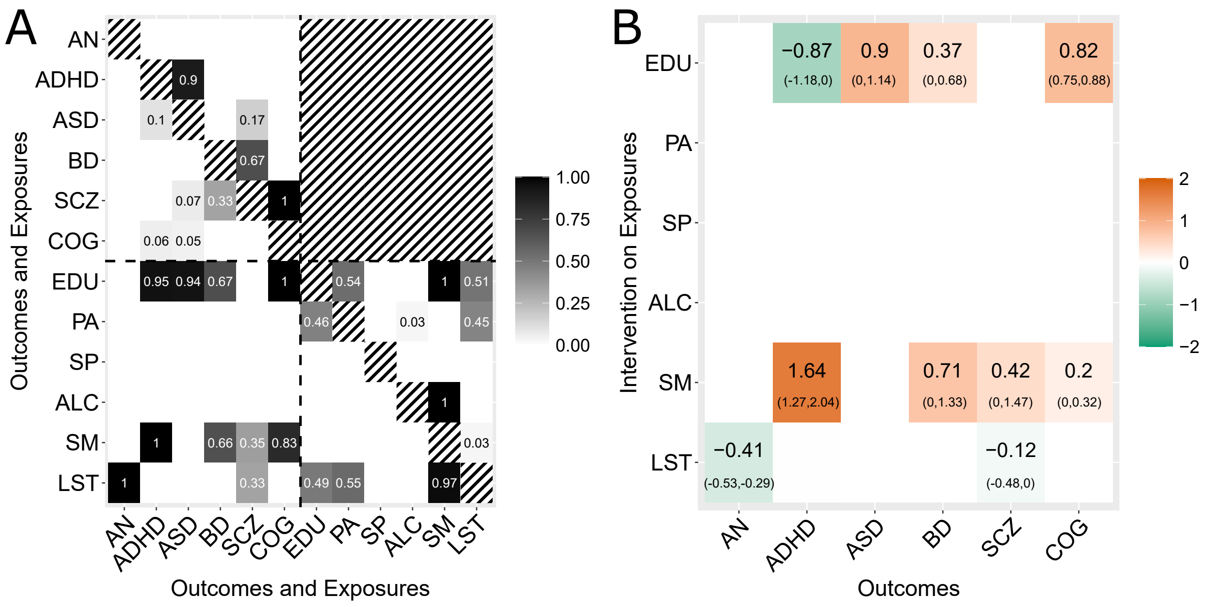

Figure S18. Results of MrDAG regarding how lifestyle and behavioural exposures impact mental health outcomes when major depressive disorder (MDD) is removed from the list of outcomes. (**A**) Posterior probability of edge inclusion (PPEI) for each combination of outcomes (mental health phenotypes) and exposures (lifestyle and behavioural traits) when MDD is removed from the list of outcomes. Horizontal and vertical dotted lines separate the exposures (bottom-right submatrix) from the outcomes (top-left submatrix). PPEIs between exposures and outcomes are depicted in the bottom-left submatrix. Neither reverse causation (top-right submatrix) nor phenotypic traits feedback loops (main diagonal) are allowed (black-white strips). (**B**) Posterior causal effects (95% credible intervals) on the outcomes (y-axis) under intervention on the exposures (x-axis) when MDD is excluded from the list of outcomes. The results are obtained by specifying the same prior probability of edge inclusion $\pi^{\text{edge}} = 0.16$ used in the main text Section 'Real data application: The impact of lifestyle and behavioural traits on mental health'.

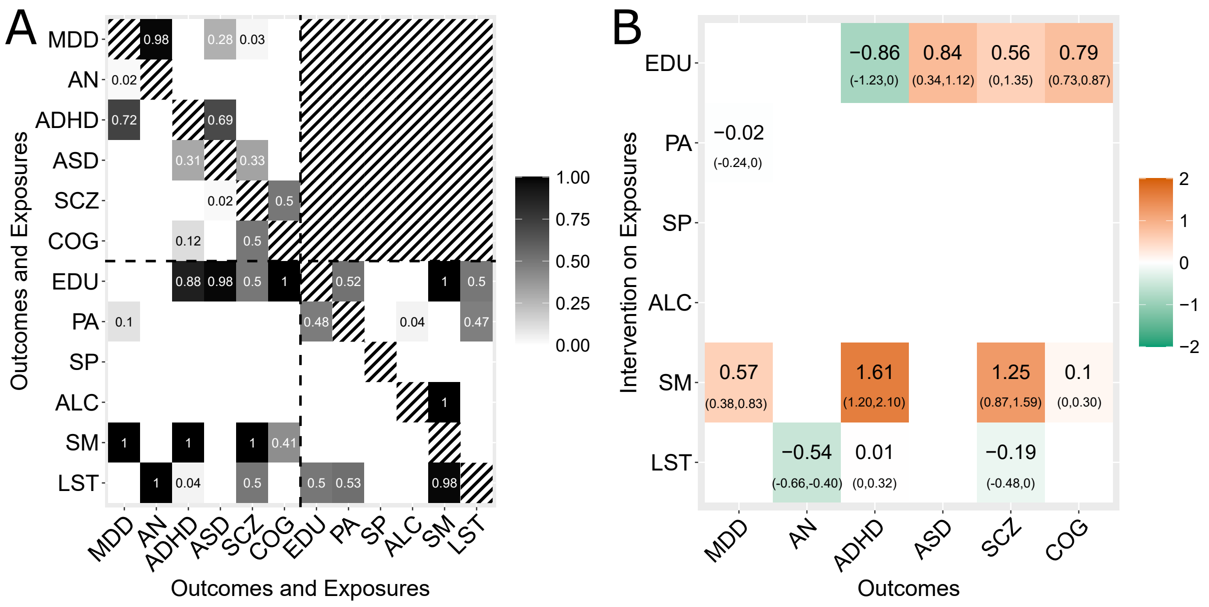

Figure S19. Results of MrDAG regarding how lifestyle and behavioural exposures impact mental health outcomes when major depressive disorder (BD) is removed from the list of outcomes. (**A**) Posterior probability of edge inclusion (PPEI) for each combination of outcomes (mental health phenotypes) and exposures (lifestyle and behavioural traits) when BD is removed from the list of outcomes. Horizontal and vertical dotted lines separate the exposures (bottom-right submatrix) from the outcomes (top-left submatrix). PPEIs between exposures and outcomes are depicted in the bottom-left submatrix. Neither reverse causation (top-right submatrix) nor phenotypic traits feedback loops (main diagonal) are allowed (black-white strips). (**B**) Posterior causal effects (95% credible intervals) on the outcomes (y-axis) under intervention on the exposures (x-axis) when BD is excluded from the list of outcomes. The results are obtained by specifying the same prior probability of edge inclusion $\pi^{\text{edge}} = 0.16$ used in the main text Section 'Real data application: The impact of lifestyle and behavioural traits on mental health'.

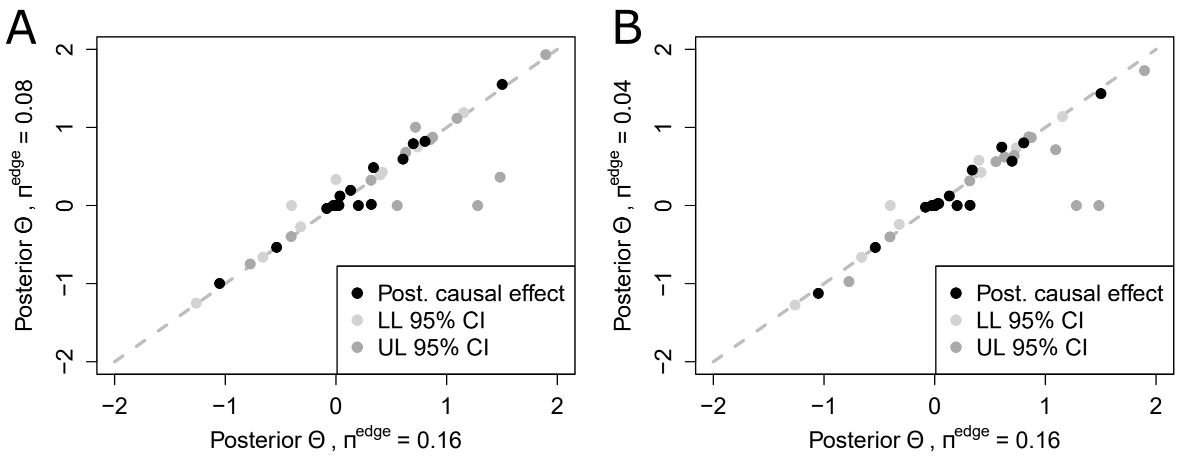

Figure S20. Sensitivity analysis of MrDAG results regarding how lifestyle and behavioural exposures impact mental health outcomes for different values of the *a priori* probability of edge inclusion. Posterior causal effects (black dots) and 95% credible intervals (Lower Limit - light-grey dots, Upper Limit - dark-grey dots) obtained by MrDAG algorithm for different levels of sparsity controlled by the hyper-parameter π^{edge}. In each panel, the x-axis reports the (Bayesian model-averaged) causal effects at $\pi^{\text{edge}} = 0.16$ used in the main text Section 'Real data application: The impact of lifestyle and behavioural traits on mental health' and in the y-axis the (Bayesian model-averaged) causal effects at (**A**) $\pi^{\text{edge}} = 0.08$ and (**B**) $\pi^{\text{edge}} = 0.04$. Overall, MrDAG algorithm is robust with only a handful of cases where the causal effects and the credible intervals do not agree at different levels of π^{edge}.

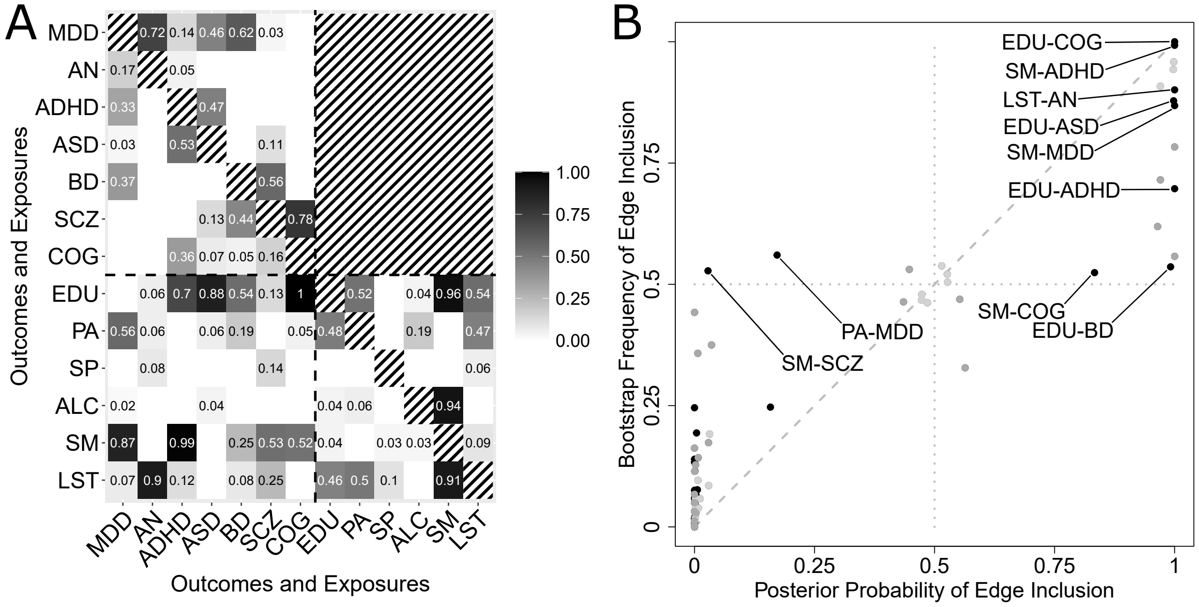

Figure S21. Robustness of MrDAG results regarding how lifestyle and behavioural exposures impact mental health outcomes. (**A**) Bootstrap frequency of edge inclusion is calculated as the average of the posterior probability of edge inclusion (PPEI) obtained by running MrDAG algorithm on each bootstrap summary-level statistics. Horizontal and vertical dotted lines separate the exposures (bottom-right submatrix) from the outcomes (top-left submatrix). Bootstrap frequencies between exposures and outcomes are depicted in the bottom-left submatrix. (**B**) Scatterplot of PPEI against the bootstrap frequency of edge inclusion. For values of the bootstrap frequency of edge inclusion (y-axis) ≥ 0.5 (horizontal dotted line), exposure-outcome pairs (black dots) are highlighted.

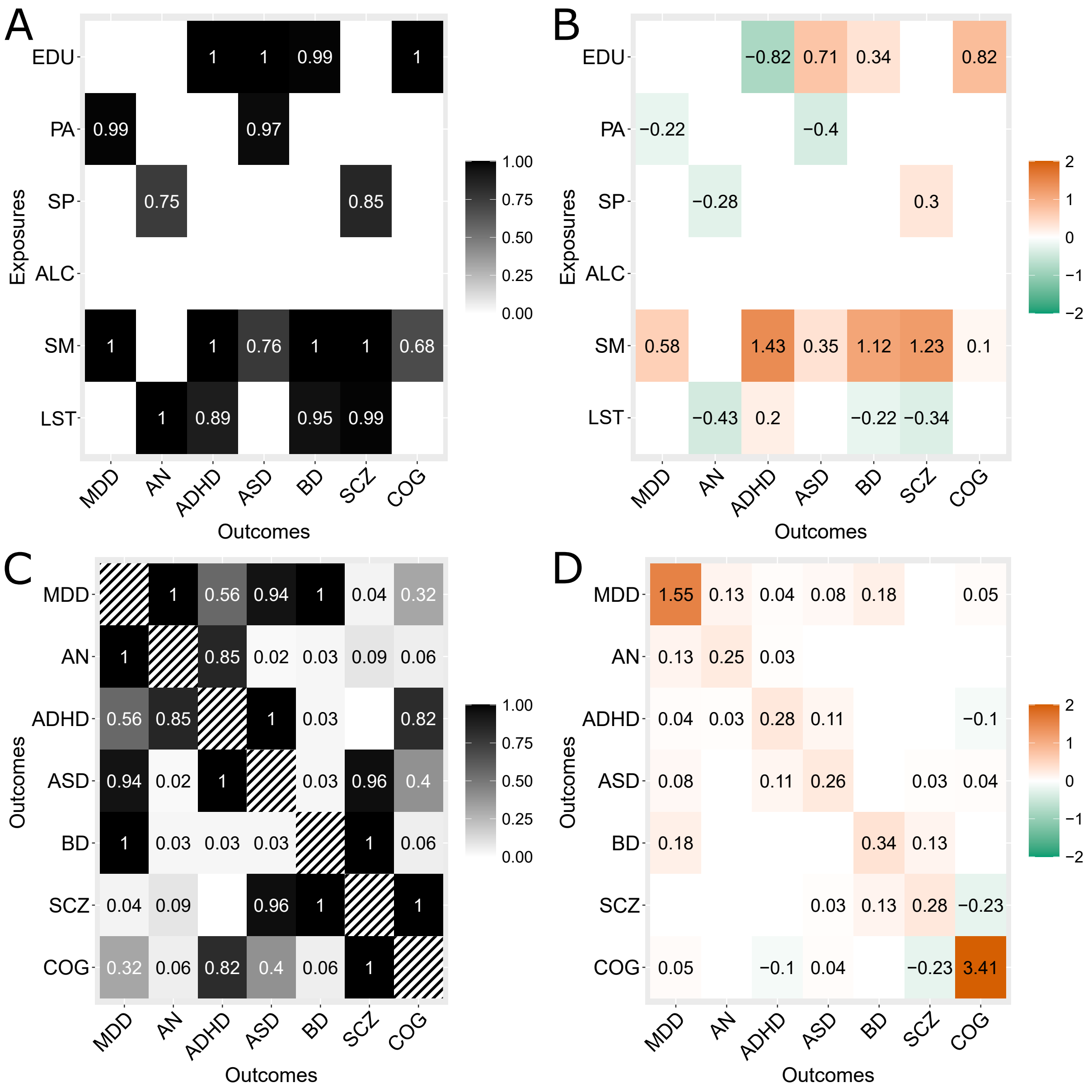

Figure S22. Results of the MR^2 algorithm regarding how lifestyle and behavioural exposures impact mental health outcomes. (**A**) Marginal posterior probability of inclusion (mPPI) thresholded at 5% FDR[3] measuring the strength of the association between the exposures (x-axis) on the outcomes (y-axis). (**B**) Posterior mean of the direct causal effects of the exposures (x-axis) on the outcomes (y-axis) at 5% FDR on the mPPIs. (**C**) Marginal posterior probability of edge inclusion of the decomposable graphical model between the responses. (**D**) Posterior mean of the precision (inverse of the covariance matrix) between the responses.

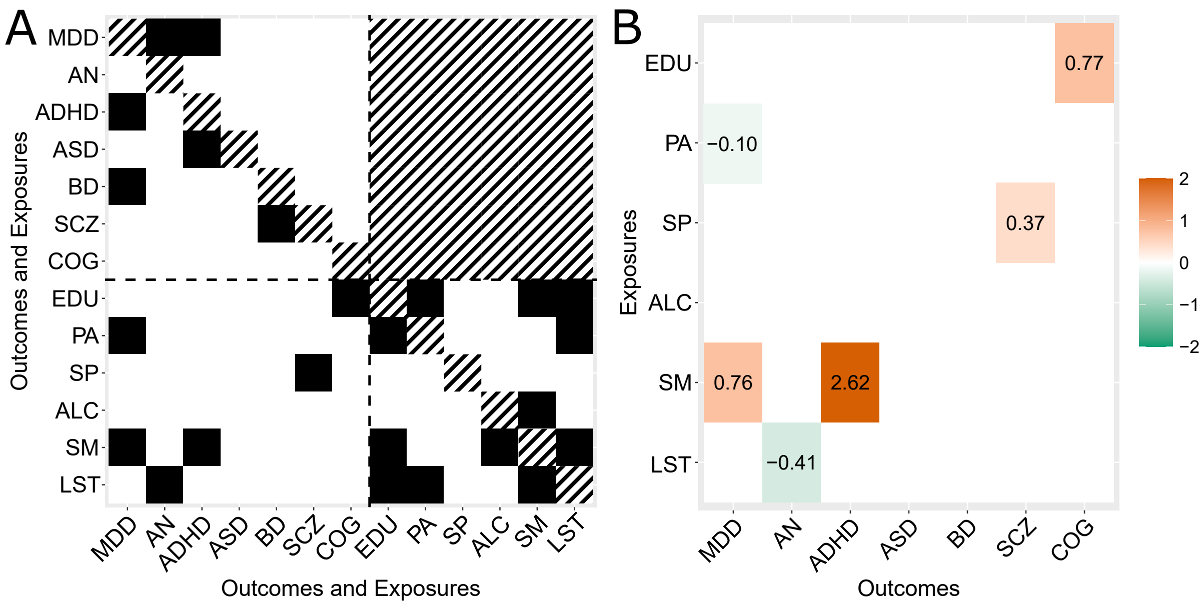

Figure S23. Results of the MRPC algorithm regarding how lifestyle and behavioural exposures impact mental health outcomes obtained at type I error rate for the conditional independence test $\alpha = 0.01$ which provides the best results in the simulation study. (**A**) Detected dependency relations between outcomes (mental health phenotypes) and exposures (lifestyle and behavioural traits). Horizontal and vertical dotted lines separate the exposures (bottom-right submatrix) from the outcomes (top-left submatrix). Detected causal associations between exposures and outcomes are depicted in the bottom-left submatrix. Neither reverse causation (top-right submatrix) nor phenotypic traits feedback loops (main diagonal) are allowed (black-white strips). (**B**) Estimated causal effects of the exposures (x-axis) on the outcomes (y-axis).

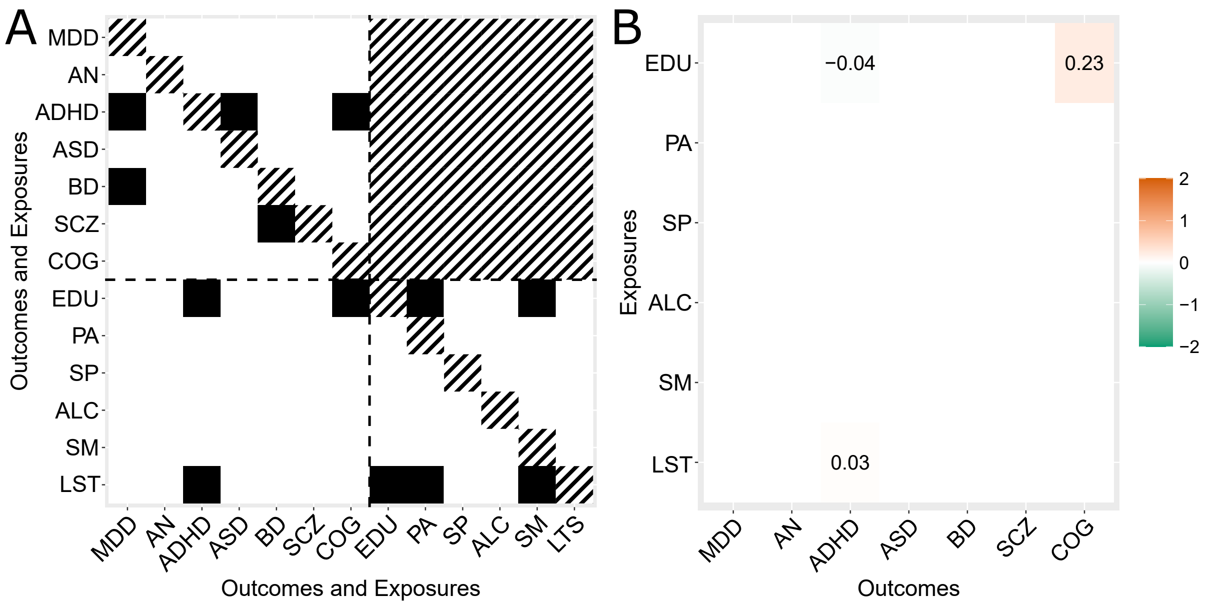

Figure S24. Results of the ParDAG algorithm regarding how lifestyle and behavioural exposures impact mental health outcomes obtained at Lasso penalisation $\lambda = 0.9$ which provides the best results in the simulation study. We also tried a stability selection approach to set the penalisation parameter as suggested in[12]. The estimated penalisation parameter $\widehat{\lambda} = 0.95$ is very close to the value used in the analysis with no appreciable differences regarding the estimated causal effects. (**A**) Detected dependency relations between outcomes (mental health phenotypes) and exposures (lifestyle and behavioural traits). Horizontal and vertical dotted lines separate the exposures (bottom-right submatrix) from the outcomes (top-left submatrix). Detected causal associations between exposures and outcomes are depicted in the bottom-left submatrix. Neither reverse causation (top-right submatrix) nor phenotypic traits feedback loops (main diagonal) are allowed (black-white strips). (**B**) Estimated causal effects of the exposures (x-axis) on the outcomes (y-axis).

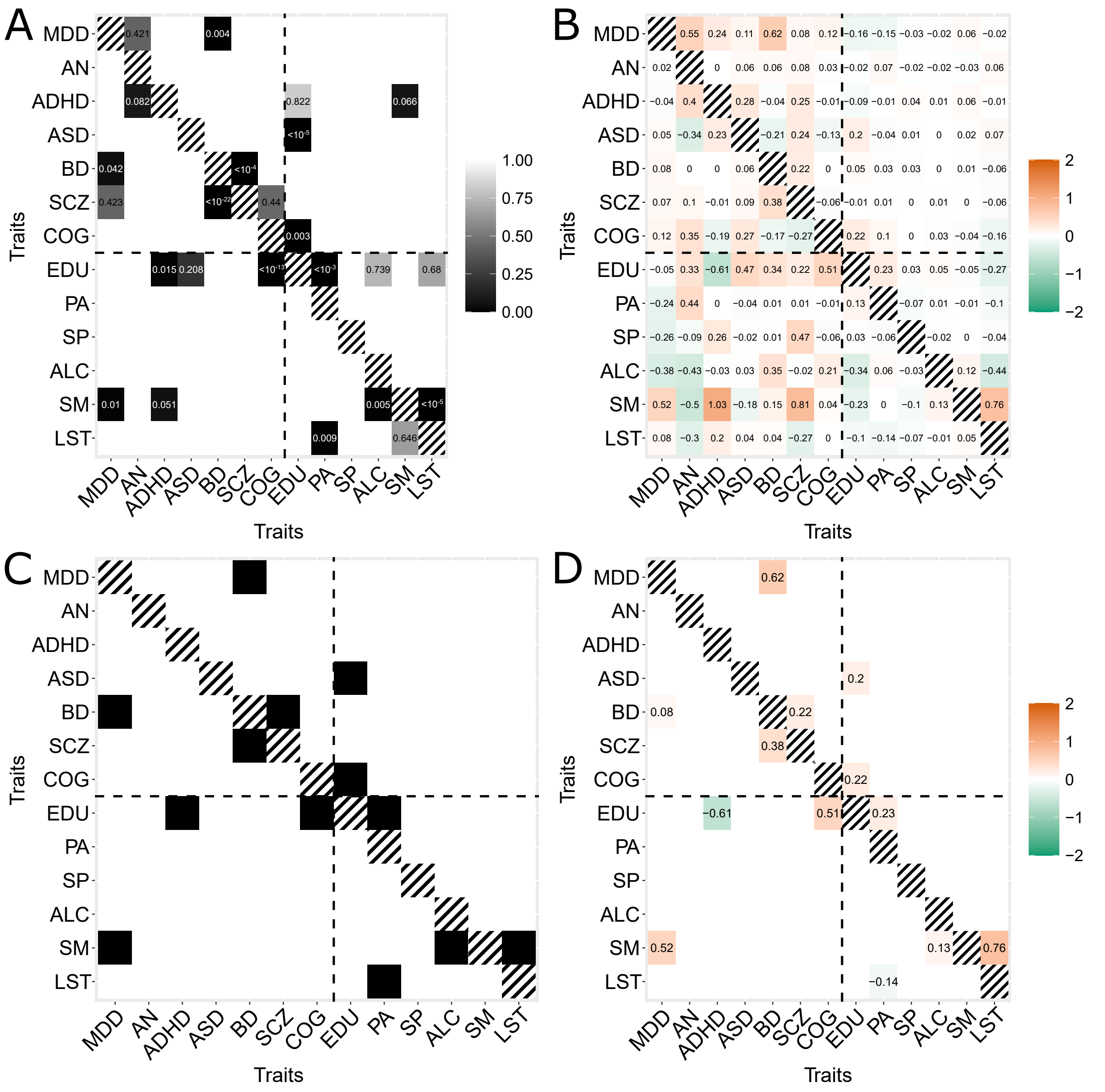

Figure S25. Results of the Graph-MRcML algorithm. Graph-MRcML treats all traits as equal and calculates bidirectional MR between all traits with no distinction between exposures and outcomes. To facilitate the comparisons with MrDAG and other "unidirectional" MR methods, horizontal and vertical dotted lines separate the traits into exposures and outcomes. (**A**) Bonferroni-adjusted p-values. Values equal to 1 not shown. (**B**) Direct causal effects among all the trait pairs. (**C**) Selected trait pairs at 5% Bonferroni-adjusted significance level. (**D**) Direct causal effects for selected trait pairs at 5% Bonferroni-adjusted significance level.

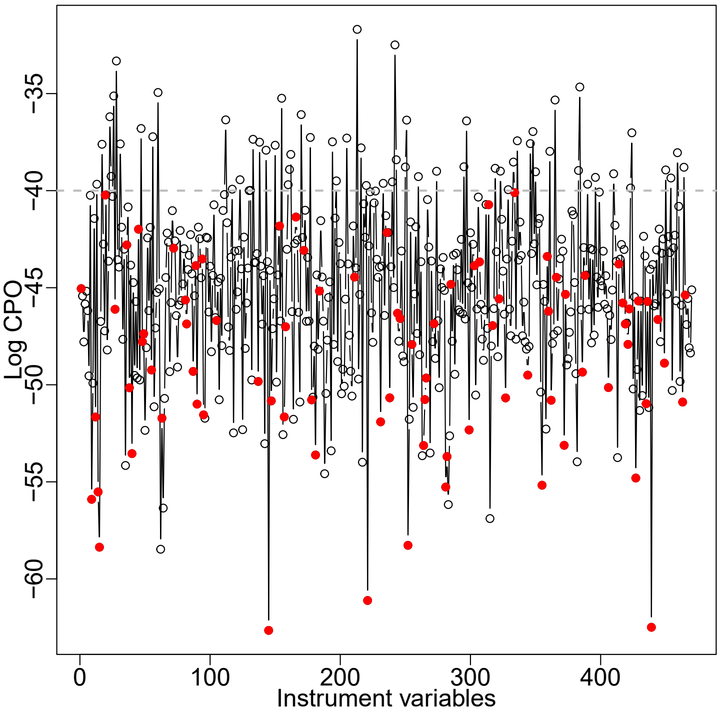

Figure S26. Invalid instrumental variables (IVs) detection by using MrDAG output in the real data application regarding how liability to mental health phenotypes affects lifestyle and behavioural traits. Log-conditional predictive ordinate (CPO) (y-axis) is plotted against 470 IVs (x-axis). 84 (16%) IVs with scaled CPOs below 0.01 to be regarded as outliers are detected (red dots)[38]. Although the large majority of IVs (86%) show log-inverse-CPOs larger than 40 (possible outliers) no one is higher than 70 (extreme values)[39].

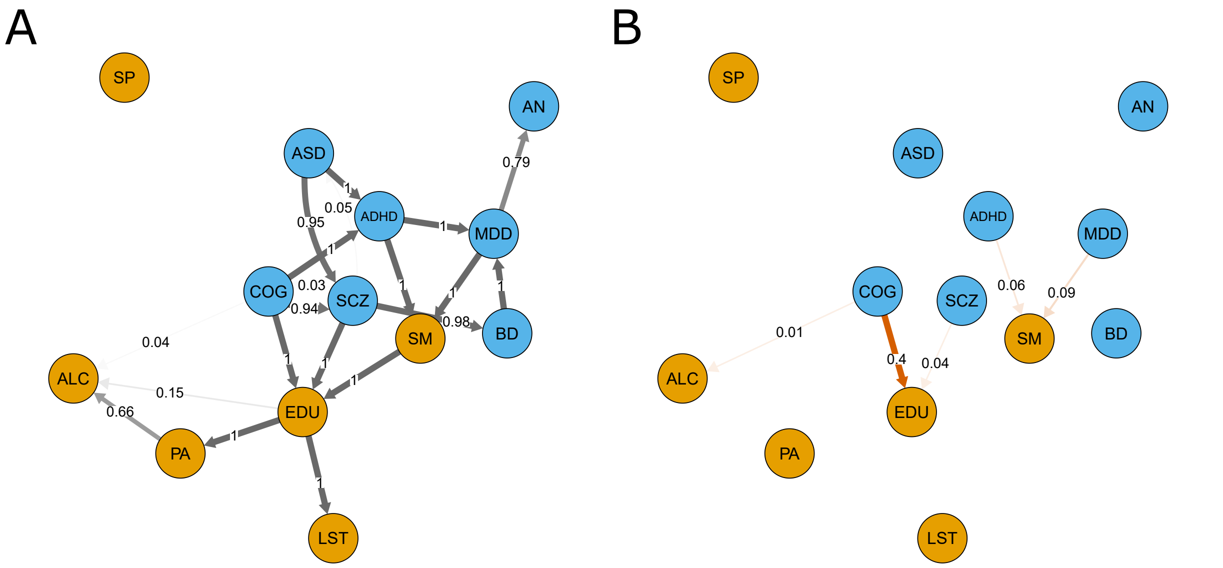

Figure S27. Partially DAGs (PDAGs) representation of MrDAG results regarding how liability to mental health phenotypes affects lifestyle and behavioural traits after removing invalid IVs. (**A**) PDAG of the posterior probability of edge inclusion (PPEI) (14) within the exposures (mental health phenotypes, blue nodes), the outcomes (lifestyle and behavioural traits, orange nodes) and between them. Undirected edges are represented as bidirectional edges, see, for instance, edges between SP (sleeping) and ALC (alcohol) or SCZ (schizophrenia) and BD (bipolar disorder). Neither reverse causation from the outcomes to the exposures nor phenotypic traits feedback loops are allowed. (**B**) (Bayesian model-averaged) causal effects on the outcomes (orange nodes) under intervention on the exposures (blue nodes). Red and green edges indicate positive and negative causal effects, respectively.

References

[1] Hernán, M. A. & Robins, J. M. Instruments for causal inference. An epidemiologist's dream? *Epidemiology* **17**, 360–372 (2006). URL https://doi.org/10.1097/01.ede.0000222409.00878.37.

[2] Didelez, V. & Sheehan, N. Mendelian randomization as an instrumental variable approach to causal inference. *Statistical Methods in Medical Research* **16**, 309–330 (2007). URL https://doi.org/10.1177/0962280206077743.

[3] Zuber, V. *et al.* Multi-response Mendelian randomization: Identification of shared and distinct exposures for multimorbidity and multiple related disease outcomes. *The American Journal of Human Genetics* **110**, 1177–1199 (2023). URL https://doi.org/10.1016/j.ajhg.2023.06.005.

[4] Pearl, J. [Bayesian analysis in expert systems]: Comment: Graphical models, causality and intervention. *Statistical Science* **8**, 266–269 (1993). URL https://doi.org/10.1214/ss/1177010894.

[5] Castelletti, F., Consonni, G., Vedova, M. L. D. & Peluso, S. Learning Markov equivalence classes of Directed Acyclic Graphs: An objective Bayes approach. *Bayesian Analysis* **13**, 1235–1260 (2018). URL https://doi.org/10.1214/18-ba1101.

[6] Castelletti, F. & Consonni, G. Bayesian inference of causal effects from observational data in Gaussian graphical models. *Biometrics* **77**, 136–149 (2021). URL https://doi.org/10.1111/biom.13281.

[7] Glymour, C., Zhang, K. & Spirtes, P. Review of causal discovery methods based on graphical models. *Frontiers in Genetics* **10** (2019). URL https://doi.org/10.3389/fgene.2019.00524.

[8] Zuber, V., Colijn, J. M., Klaver, C. & Burgess, S. Selecting likely causal risk factors from high-throughput experiments using multivariable Mendelian randomization. *Nature Communications* **11**, 29 (2020). URL https://doi.org/10.1038/s41467-019-13870-3.

[9] Badsha, M. B., Martin, E. A. & Fu, A. Q. MRPC: An R package for inference of causal graphs. *Frontiers in Genetics* **12** (2021). URL https://doi.org/10.3389/fgene.2021.651812.

[10] Badsha, M. B. & Fu, A. Q. Learning causal biological networks with the principle of Mendelian randomization. *Frontiers in Genetics* **10** (2019). URL https://doi.org/10.3389/fgene.2019.00460.

[11] Kalisch, M., Mächler, M., Colombo, D., Maathuis, M. H. & Bühlmann, P. Causal inference using graphical models with the R package pcalg. *Journal of Statistical Software* **47** (2012). URL https://doi.org/10.18637/jss.v047.i11.

[12] Rahman, S., Khare, K., Michailidis, G., Martínez, C. & Carulla, J. Estimation of Gaussian irected Acyclic Graphs using partial ordering information with applications to DREAM3 networks and dairy cattle data. *The Annals of Applied Statistics* **17**, 929–960 (2023). URL https://doi.org/10.1214/22-aoas1636.

[13] Rahman, S. PDAG (2021). URL https://github.com/shr264/PDAG. R package version 1.0.0.

[14] Lin, Z. Graph-MRcML (2023). URL https://github.com/ZhaotongL/GraphMRcML. R package version 1.0.0.

[15] Feizi, S., Marbach, D., Médard, M. & Kellis, M. Network deconvolution as a general method to distinguish direct dependencies in networks. *Nature Biotechnology* **31**, 726–733 (2013). URL https://doi.org/10.1038/nbt.2635.

[16] Xue, H., Shen, X. & Pan, W. Constrained maximum likelihood-based Mendelian randomization robust to both correlated and uncorrelated pleiotropic effects. *The American Journal of Human Genetics* **108**, 1251–1269 (2021). URL https://doi.org/10.1016/j.ajhg.2021.05.014.

[17] Kalisch, M. & Bühlmann, P. Estimating high-dimensional Directed Acyclic Graphs with the PC-algorithm. *Journal of Machine Learning Research* **8**, 613–636 (2007). URL https://jmlr.csail.mit.edu/papers/v8/kalisch07a.html.

[18] Hauser, A. & Bühlmann, P. Characterization and greedy learning of interventional Markov equivalence classes of Directed Acyclic Graphs. *Journal of Machine Learning Research* **13**, 2409–2464 (2012). URL https://jmlr.org/papers/v13/hauser12a.html.

[19] Andersson, S. A., Madigan, D. & Perlman, M. D. A characterization of Markov equivalence classes for acyclic digraphs. *Annals of Statistics* **25**, 505–541 (1997). URL https://doi.org/10.1214/aos/1031833662.

[20] Lee, J. J. *et al.* Gene discovery and polygenic prediction from a genome-wide association study of educational attainment in 1.1 million individuals. *Nature Genetics* **50**, 1112–1121 (2018). URL https://doi.org/10.1038/s41588-018-0147-3.

[21] Wang, Z. *et al.* Genome-wide association analyses of physical activity and sedentary behavior provide insights into underlying mechanisms and roles in disease prevention. *Nature Genetics* **54**, 1332–1344 (2022). URL https://doi.org/10.1038/s41588-022-01165-1.

[22] Dashti, H. S. *et al.* Genome-wide association study identifies genetic loci for self-reported habitual sleep duration supported by accelerometer-derived estimates. *Nature Communications* **10**, 1100 (2019). URL https://doi.org/10.1038/s41467-019-08917-4.

[23] Evangelou, E. *et al.* New alcohol-related genes suggest shared genetic mechanisms with neuropsychiatric disorders. *Nature Human Behaviour* **3**, 950–961 (2019). URL https://doi.org/10.1038/s41562-019-0653-z.

[24] Wootton, R. E. *et al.* Evidence for causal effects of lifetime smoking on risk for depression and schizophrenia: A Mendelian randomisation study. *Psychological Medicine* **50**, 2435–2443 (2020). URL https://doi.org/10.1017/S0033291719002678.

[25] Howard, D. M. *et al.* Genome-wide meta-analysis of depression identifies 102 independent variants and highlights the importance of the prefrontal brain regions. *Nature*

Neuroscience **22**, 343–352 (2019). URL https://doi.org/10.1038/s41593-018-0326-7.

[26] Watson, H. J. *et al.* Genome-wide association study identifies eight risk loci and implicates metabo-psychiatric origins for anorexia nervosa. *Nature Genetics* **51**, 1207–1214 (2019). URL https://doi.org/110.1038/s41588-019-0439-2.

[27] Demontis, D. *et al.* Discovery of the first genome-wide significant risk loci for attention deficit/hyperactivity disorder. *Nature Genetics* **51**, 63–75 (2019). URL https://doi.org/10.1038/s41588-018-0269-7.

[28] Mullins, N. *et al.* Genome-wide association study of more than 40,000 bipolar disorder cases provides new insights into the underlying biology. *Nature Genetics* **53**, 817–829 (2021). URL https://doi.org/10.1038/s41588-021-00857-4.

[29] Grove, J. *et al.* Identification of common genetic risk variants for autism spectrum disorder. *Nature Genetics* **51**, 431–444 (2019). URL https://doi.org/10.1038/s41588-019-0344-8.

[30] Trubetskoy, V. *et al.* Mapping genomic loci implicates genes and synaptic biology in schizophrenia. *Nature* **604**, 502–508 (2022). https://doi.org/10.1038/s41586-022-04434-5.

[31] Burgess, S., Foley, C. N. & Zuber, V. Inferring causal relationships between risk factors and outcomes from genome-wide association study data. *Annual Review of Genomics and Human Genetics* **19**, 303–327 (2018). URL https://doi.org/10.1146/annurev-genom-083117-021731.

[32] Benjamini, Y. & Hochberg, Y. Controlling the false discovery rate: a practical and powerful approach to multiple testing. *Journal of the Royal Statistical Society: Series B (Methodological)* **57**, 289–300 (1995). URL https://doi.org/10.1111/j.2517-6161.1995.tb02031.x.

[33] Zuber, V. *et al.* High-throughput multivariable Mendelian randomization analysis prioritizes apolipoprotein B as key lipid risk factor for coronary artery disease. *International Journal of Epidemiology* **50**, 893–901 (2021). URL https://doi.org/10.1093/ije/dyaa216.

[34] Didelez, V. Causal concepts and graphical models. In Maathuis, M., Drton, M., Lauritzen, S. & Wainwright, M. (eds.) *Handbook of Graphical Models*, 353–376 (Chapman and Hall/CRC, Boca Raton, FL, 2018). URL https://doi.org//10.1201/9780429463976-15.

[35] Perković, E., Textor, J., Kalisch, M. & Maathuis, M. H. A complete generalized adjustment criterion. In Meila, M. & Heskes, T. (eds.) *Proceedings UAI*, 682–691 (AUAI Press, 2015). URL http://auai.org/uai2015/proceedings/papers/155.pdf.

[36] Sing, T., Sander, O., Beerenwinkel, N. & Lengauer, T. ROCR: Visualizing classifier performance in R. *Bioinformatics* **21**, 3940–3941 (2005). URL https://doi.org/10.1093/bioinformatics/bti623.

[37] Sing, T. *et al.* ROCR (2020). URL `https://ipa-tys.github.io/ROCR/`. R package version 1.0.11.

[38] Congdon, P. *Bayesian Models for Categorical Data* (John Wiley & Sons, Chichester, 2005).

[39] Ntzoufras, I. *Bayesian Modeling Using WinBUGS* (Wiley, Hoboken, N.J., 2009). URL `https://doi.org/10.1002/9780470434567`.
